# Unveiling forensically relevant biogeographic, phenotype and Y-chromosome SNP variation in Pakistani ethnic groups using a customized hybridisation enrichment forensic intelligence panel

**DOI:** 10.1371/journal.pone.0264125

**Published:** 2022-02-17

**Authors:** Sobiah Rauf, Jeremy J. Austin, Denice Higgins, Muhammad Ramzan Khan

**Affiliations:** 1 Genome Editing & Sequencing Lab, National Center for Bioinformatics, Quaid-i-Azam University, Islamabad, Pakistan; 2 Australian Centre for Ancient DNA (ACAD), School of Biological Sciences, The University of Adelaide, Adelaide, South Australia, Australia; 3 School of Dentistry, Health and Medical Sciences, The University of Adelaide, Adelaide, South Australia, Australia; Banaras Hindu University, INDIA

## Abstract

Massively parallel sequencing following hybridisation enrichment provides new opportunities to obtain genetic data for various types of forensic testing and has proven successful on modern as well as degraded and ancient DNA. A customisable forensic intelligence panel that targeted 124 SNP markers (67 ancestry informative markers, 23 phenotype markers from the HIrisplex panel, and 35 Y-chromosome SNPs) was used to examine biogeographic ancestry, phenotype and sex and Y-lineage in samples from different ethnic populations of Pakistan including Pothwari, Gilgit, Baloach, Pathan, Kashmiri and Siraiki. Targeted sequencing and computational data analysis pipeline allowed filtering of variants across the targeted loci. Study samples showed an admixture between East Asian and European ancestry. Eye colour was predicted accurately based on the highest p-value giving overall prediction accuracy of 92.8%. Predictions were consistent with reported hair colour for all samples, using the combined highest p-value approach and step-wise model incorporating probability thresholds for light or dark shade. Y-SNPs were successfully recovered only from male samples which indicates the ability of this method to identify biological sex and allow inference of Y-haplogroup. Our results demonstrate practicality of using hybridisation enrichment and MPS to aid in human intelligence gathering and will open many insights into forensic research in South Asia.

## Introduction

In forensic investigations, massively parallel sequencing (MPS) with the ability to genotype multiple markers in various biological samples in a single assay with small DNA amount, delivers the potential to enhance human identification and forensic intelligence gathering. It also provides benefits in a number of areas such as admixture analysis, solving complex paternal/maternal cases. leading to an increase in the performance and cost/time-effectiveness of sensitive legal cases [1]. Identification of a person and relatedness between individuals are two of the leading matters in forensic analysis.

The potential of single nucleotide polymorphisms (SNPs) to be utilized as genetic markers has made them enormously popular especially in the field of forensic DNA analysis because of various qualities they possess such as automation ability, small fragment length and frequency in the genome [2]. SNPs are more stable genetic markers in most of the sensitive situations such as ancestry cases like inheritance/kinship, provides investigative lead value in cases having no genetic profile match in DNA databases or with no suspect, and in family reconstructions in case of missing individuals and unknown human remains (where the DNA is significantly fragmented). This is because of the fact that they have comparatively low mutation rates [3]. SNP variation in pigmentation genes can also be useful for inferring visible phenotypic traits for example hair, skin and eye colour [4].

For forensic identification purposes targeted enrichment combined with massively parallel sequencing has been explored recently which targets mtDNA and nuclear SNPs [5, 6]. Commercial MPS panels using standard PCR-based target enrichment have been developed to genotype many forensically relevant markers [7–9]. Hybridisation enrichment, an alternative approach to PCR-based target enrichment prior to sequencing, uses biotinylated probes (complementary to target regions in a DNA sample) to bind to target DNA and has proven successful on modern as well as degraded and ancient DNA [10]. This strategy can enrich for SNP loci prior to sequencing without the need for an initial PCR. Streptavidin beads magnetize to probes bound to target DNA, while unbound DNA and impurities are eliminated through a series of stringency washes. Hybridization enrichment can eliminate some issues with PCR-based approaches, particularly for primer design, and as a result much shorter fragment lengths of DNA can be captured without the need for intact PCR primer binding sites [11]. There is no requirement for complex PCR primer multiplex design for large numbers of markers and thus no limit on how many loci can be examined in a single assay [12].

The aim of the present study is to explore the implementation of emerging target enrichment and massively parallel sequencing technologies to genotype forensically relevant SNPs in samples from different ethnic populations of Pakistan. We used a customized 124-SNP forensic intelligence panel that offered a combined suite of phenotype, biogeographic ancestry and Y-chromosome (Y-chr) SNPs for comprehensive biological profiling.

## Materials and methods

**Fig 1:**
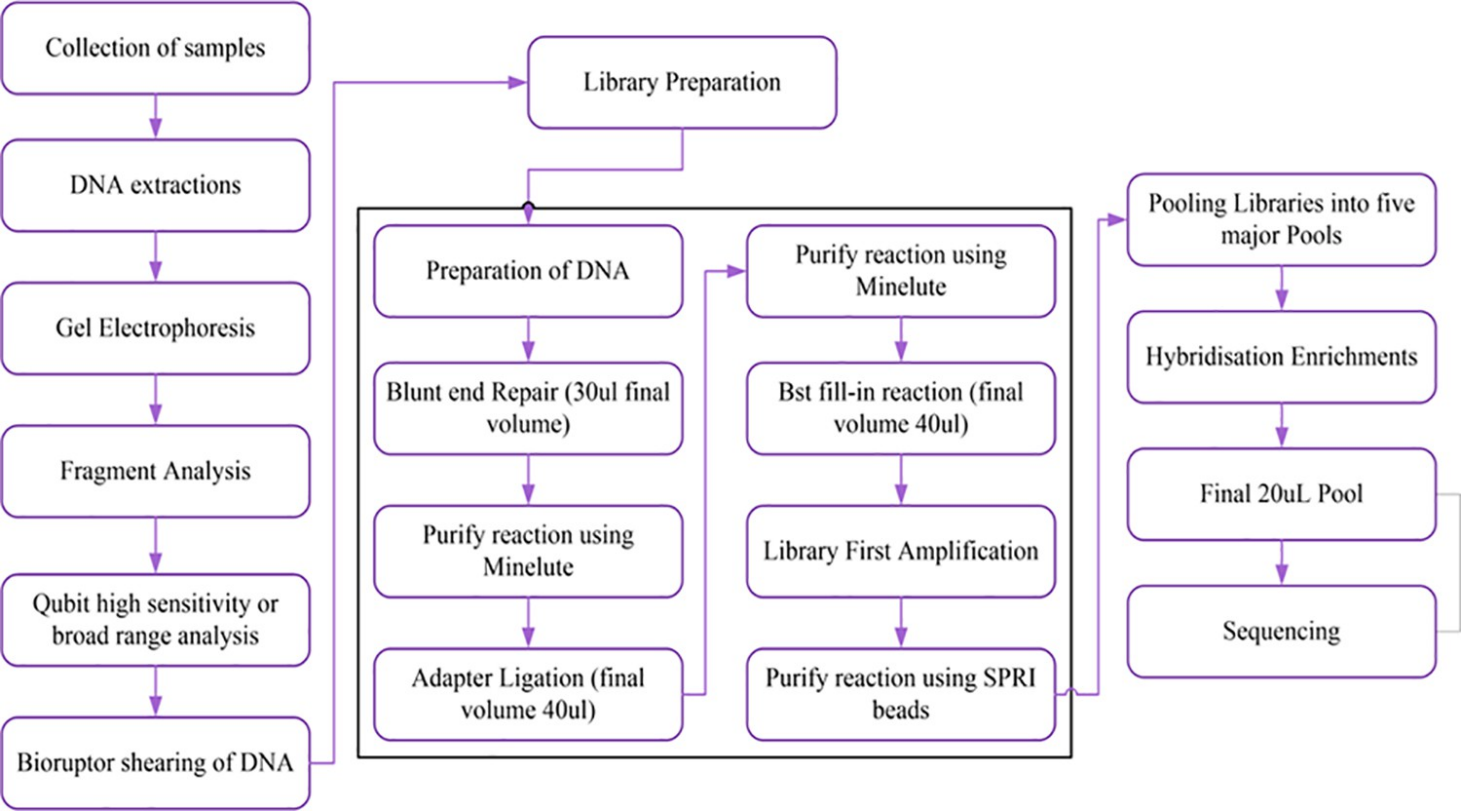
Summary of steps in experimental lab workflow.

A step by step workflow for the experimental lab work is presented in Fig 1.

### Collection of study samples and DNA extraction

Blood samples were collected from twenty-eight unrelated healthy male and female individuals belonging to different ethnic populations (Pothwari, Pathan, Baloach, Kashmiri, Gilgit and Siraiki) of Pakistan. Written acceptance was obtained from all donors with approval from the Institutional Bioethics Committee (IBC) No. #BEC-FBS-QAU2018-4. Donors had a self-declared ancestry, sex, and different combinations of eye and hair colour. DNA was extracted using a PureLinkTM Genomic DNA kit (Thermo Fisher Scientific Inc., Waltham, Massachusetts, USA) following the manufacturer’s protocol.

### Library preparation and hybridisation enrichment

Genomic DNA was sheared, converted into truncated Illumina libraries and enriched (via hybridisation to 5’ biotinylated 120-mer DNA oligonucleotides (xGen Lockdown Probes) as described by Bardan (2019) [13]. A total of 125 nuclear SNPs (67 ancestry-informative; Table 1, 23 phenotypic; Table 2, and 35 Y-chromosome; Table 3, with one SNP shared between ancestry and phenotype) were include in the bait set. The 124 SNPs provide broad categorisation of continental biogeographic ancestry (African, European, Asian, Native American and Oceanian), major Y-chromosome haplogroups and hair/eye colour prediction, and were developed as a customisable panel for forensic intelligence gathering (Bardan 2019).

**Table 1:** Detailed information of the 67 biogeographic ancestry SNPs included in the hybridisation enrichment panel.

rs Number	Chr. No.	Position (GRCh37/hg19)	Ancestry Group	Reference
rs2139931	1	84590527	OCE	[14, 15]
rs2814778	1	159174683	AFR	[14]
rs4657449	1	165465281	EAS	[14]
rs12142199	1	1249187	EUR	[14]
rs12402499	1	101528954	AMR	[14]
rs647325	1	18170886	AMR	[16]
rs2184030	1	206667441	Tri-allelic	[15]
rs16830500	2	152814129	OCE	[17]
rs3827760	2	109513601	EAS	[14, 15]
rs10183022	2	237481969	OCE	[15]
rs820371	3	123404711	EUR	[17]
rs6437783	3	108172817	EAS	[14]
rs9809818	3	71480566	OCE	[14, 15]
rs12498138	3	121459589	AMR	[14]
rs4683510	3	140285115	EAS	[15]
rs7623065	3	22385375	OCE	[15]
rs10012227	4	18637315	AMR	[17]
rs1229984	4	100239319	EAS	[14]
rs4540055	4	38803255	Tri-allelic	[14]
rs1509524	4	125455038	OCE	[15]
rs6875659	5	175158653	AFR	[17]
rs16891982	5	33951693	EUR	[14]
rs4704322	5	75822474	EAS	[15]
rs6886019	5	170245846	OCE	[15]
rs10455681	6	69802502	OCE	[15]
rs2080161	7	13331150	AMR	[14]
rs798949	7	120765954	OCE	[15]
rs1871534	8	145639681	AFR	[14]
rs2409722	8	11039816	OCE	[15]
rs7832008	8	98358246	OCE	[15]
rs2789823	9	136769888	AFR	[14]
rs10811102	9	1911291	OCE	[15]
rs10970986	9	32453278	OCE	[15]
rs16913918	9	3074359	EUR	[18]
rs7084970	10	119750413	EUR	[17]
rs4749305	10	28391596	EUR	[14]
rs2274636	10	27443012	OCE	[15]
rs174570	11	61597212	AMR	[17]
rs3751050	11	9091244	OCE	[14, 15]
rs5030240	11	32424389	Tri-allelic	[14]
rs1924381	13	72321856	EUR	[17]
rs9522149	13	111827167	EUR	[14]
rs721367	13	95546650	EAS	[15]
rs730570	14	101142890	EUR	[17]
rs7151991	14	32635572	AMR	[17]
rs10483251	14	21671277	AMR	[14]
rs12434466	14	97324289	EAS	[15]
rs1834640	15	48392165	EUR	[19]
rs12594144	15	64161351	EAS	[14]
rs1426654	15	48426484	EUR	[14]
rs3784651	15	94925273	OCE	[15]
rs6494411	15	63835861	EAS	[15]
rs881929	16	31079371	EAS	[17]
rs17822931	16	48258198	EAS	[14]
rs16946159	16	48459558	OCE	[15]
rs4792928	17	42105174	AMR	[14]
rs8072587	17	19211073	EUR	[14]
rs9908046	17	53563782	OCE	[14, 15]
rs1369290	18	67691520	AFR	[17]
rs310644	20	62159504	AFR	[17]
rs2069945	20	33761837	Tri-allelic	[14, 15]
rs6054465	20	6673018	OCE	[14, 15]
rs715605	22	30640308	OCE	[14]
rs1557553	22	44760984	AMR	[14]
rs8137373	22	41729216	AMR	[14]
rs4892491	X	73422412	EAS	[15]
rs11156577	X	153660041	OCE	[15]

EUR informative SNP at rs16891982 is also included in the phenotype SNPs. Ancestry groups are: East Asian (EAS), African (AFR), EUR-European, Native American (AMR) and Oceanian (OCE). Tri-allelic SNPs are ancestry informative but also serve to monitor for contamination from more than 1 DNA donor.

**Table 2:** Details of the 23 phenotype (hair and eye colour) SNPs included in the hybridisation enrichment panel. SNP rs16891982 is also included in the ancestry SNPs.

rs Number	Chr. No.	Position (GRCh37/hg19)	Reference
rs16891982	5	33951693	[14, 4]
rs28777	5	33958959	[4]
rs4959270	6	457748	[4]
rs12203592	6	396321	[4]
rs683	9	12709305	[4]
rs1042602	11	88911696	[4]
rs1393350	11	89011046	[4]
rs12821256	12	89328335	[4]
rs2402130	14	92801203	[4]
rs12896399	14	92773663	[4]
rs12913832	15	28365618	[4]
rs1800407	15	28230318	[4]
rs1805005	16	89985844	[4]
rs1805006	16	89985918	[4]
rs2228479	16	89985940	[4]
rs11547464	16	89986091	[4]
rs1805007	16	89986117	[4]
rs201326893	16	89986122	[4]
rs1110400	16	89986130	[4]
rs1805008	16	89986144	[4]
rs885479	16	89986154	[4]
rs1805009	16	89986546	[4]
rs2378249	20	33218090	[4]

**Table 3:** Details of the 35 Y-chromosome SNPs included in the hybridisation enrichment panel.

rs Number (mutation name)	Position (GRCh37/hg19)	Y-chr haplogroup	Reference
rs2032595 (M168)	14813991	CDEF	[20]
rs3848982 (M145)	21717208	DE	[20]
rs2032602 (M174)	14954280	D	[21]
rs371443469 (V36)	6814246	E1b1b1a1b1a*	[14]
rs9306841 (M96)	21778998	E	[22]
rs9786025 (P170)	15021522	E	[20]
rs2032666 (M216)	15437564	C	[22]
rs35284970 (M130)	2734854	C	[20]
rs2032668 (M217)	15437333	C2*	[23]
rs868363758 (M347)	2877479	C1b3b*	[24]
rs9786706 (U13)	14698928	G2a2b2a1a1a1*	[14]
rs2032636 (M201)	15027529	G	[20]
rs13447371 (M282)	21764431	H2*	[22]
rs2032673 (M69)	21894058	H1a*	[20]
rs17250163 (P126)	21225770	IJ	[20]
rs9341301 (M258)	15023364	I	[20]
rs13447352 (M304)	22749853	J	[22]
rs9341313 (M267)	22741818	J1*	[14]
rs3900 (M9)	21730257	KLT	[20]
rs3902 (M11)	21730647	L	[20]
rs9341308 (M272)	22738775	T	[20]
rs2033003 (M526)	23550924	K	[22]
n/a (P308)	15409573	S	[25]
n/a (P256)	8685230	M	[26]
rs2032631 (M45)	21867787	QR	[20]
rs8179021 (M242)	15018582	Q	[20]
rs2032658 (M207)	15581983	R	[20]
rs17250535 (M420)	23473201	R1a	[14]
rs9786184 (M343)	2887824	R1b	[14]
rs9786153 (M269)	22739367	R1b1a1b*	[14]
rs9786140 (M412)	8502236	R1b1a1b1a*	[22]
rs9341278 (M231)	15469724	N	[20]
rs13447361 (M324)	2821786	O2a*	[27]
rs11575897 (M176)	2655180	O1b2*	[27]
rs13447354 (M307)	22750951	O1a1a*	[28]

*sub-haplogroup nomenclature taken from ISOGG 2018 version 13.256.

Enriched DNA for all 28 samples were combined into a single pool at 5nM concentration prior to paired end sequencing using Illumina MiSeq V2 with read length 2x150 base-pairs (300 cycles).

### Sequencing data analysis

**Fig 2:**
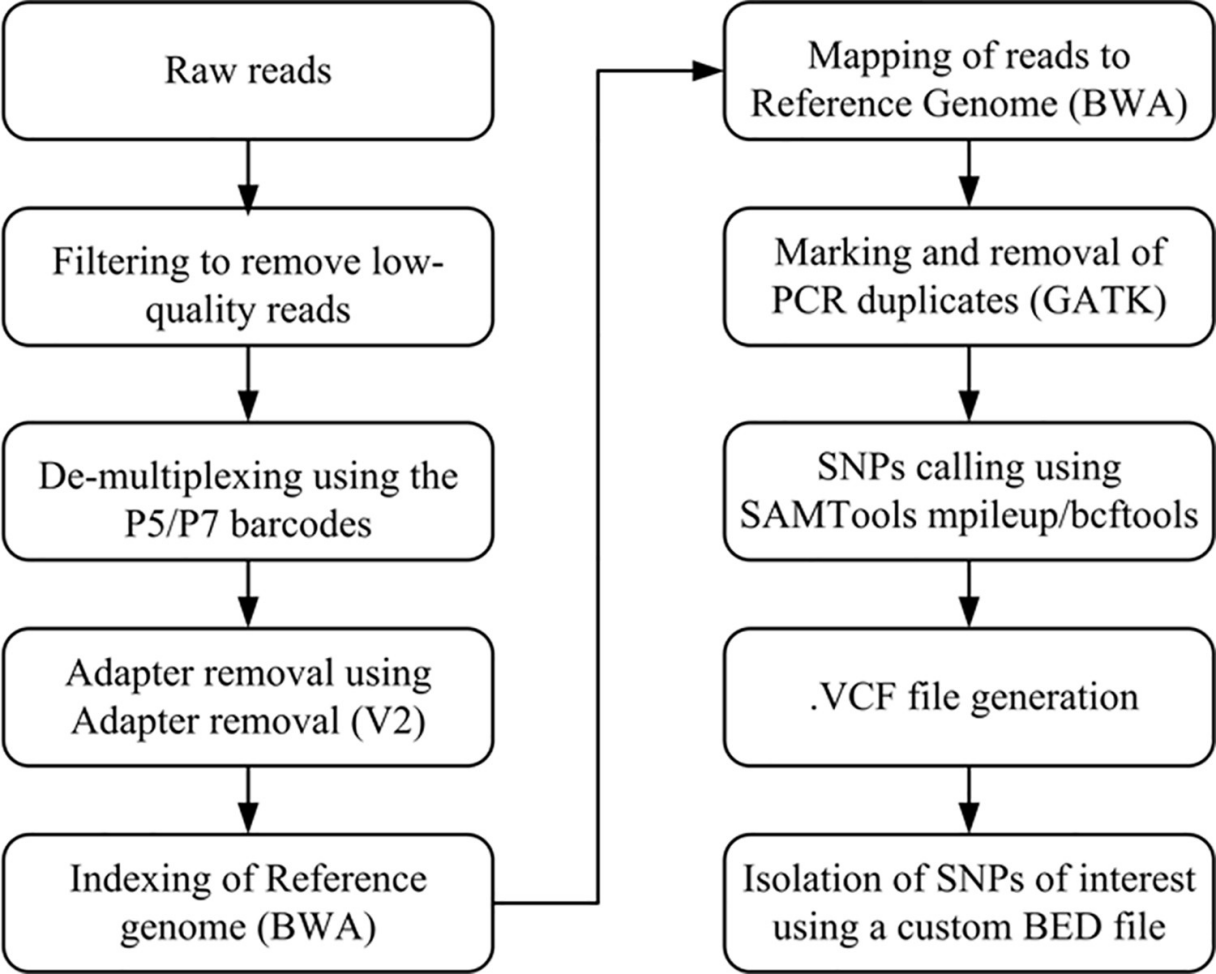
Workflow highlighting main steps of sequencing data analysis pipeline.

After sequencing of samples, reads were filtered according to the standard Illumina protocol at AGRF (Australian Genome Research Facility, Adelaide, Australia) to remove low-quality clusters, and de-multiplex by index. The raw Illumina reads were refined using the PaleoMix v1.0.1 pipeline of Schubert et al. (2014) [29]. Dual internal, P5 and P7 barcodes were used to de-multiplex sequences to each sample. To trim adapters, Adapter removal V2 [30] was used, paired reads were merged and all reads shorter than 25 base-pairs in length were eliminated. Collapsed reads were mapped to the Human Reference Genome hg19 (GRCh37) using version 0.6.2 of BWA (Burrows-Wheeler Aligner) [31]. Seeding option was disabled and a minimum mapping quality of 25 was set. PCR duplicates were eliminated so that only unique reads for genotype calling were retained. To obtain a variant calling (.vcf) file SNPs were called using SAMTools [32] mpileup/bcftools. Genotypes for the targeted SNPs of interest were then isolated by examining against a custom BED file which contains information about genomic coordinates of targeted SNP loci. A workflow summarizing key points of sequencing data analysis process is presented in Fig 2.

### DNA phenotyping

For prediction of hair and eye colour in the study samples, 23 SNPs were analyzed using the prediction model from the HIrisPlex [4] DNA Phenotyping web tool. Genotypic data as per the tool’s format was prepared in an Excel file and was input into the interface in order to generate probabilities that samples belong to a particular phenotypic class of hair and eye colour. For eye colour, the current prediction framework given by [4] says that the most likely eye colour is indicated by highest (probability) p-value. For hair colour, current interpretation guidelines combine two parameters i.e. highest p-value and shade probability values (either light or dark) to infer the most probable hair colour.

### Sex determination and inference of Y-chr haplogroup

A SNP profile was generated for each individual against thirty-five Y-chr SNPs to identify biological sex. For males the Y haplogroup was defined according to diagnostic ancestral and derived SNPs in PhyloTreeY described by Van Oven et al, 2014 [33]: http://www.phylotree.org/Y). Geographical affiliation was assigned based on the classifications and frequencies defined in previous studies [20–22, 25, 33, 34].

### BGA prediction

For biogeographic ancestry (BGA) assignment of each target sample, 67 ancestry informative SNPs from each sample genotype were compared to a reference population data set consisting of genotypes from 368 individuals belonging to different regions i.e. 99 individuals from African population (AFR), 89 from East Asian population (EAS), 88 European (EUR), 64 Native American (AMR), and 28 from Oceanian (OCE) populations. Genotypes of reference population were collected from the 1000 Genomes Project Consortium and Stanford University HGDP-CEPH [35] datasets, and were carefully selected from populations that show minimal admixture. Ancestries were assigned to each sample using Snipper [36] tool (Ancestry Information Markers classification of multiple individuals), with application of Hardy-Weinberg principle. A file prepared for the Snipper tool containing genotype information for all 67 SNPs for each reference sample and target samples under study has been provided as a table in supporting information. For estimation of ancestry, likelihood ratios (LR) for ancestry classifications were used, and principle component analysis (PCA) was performed to visualize the genetic similarities as well as differences of the target sample genotypes with the reference populations [37].

## Results

DNA was successfully extracted from the samples and after fragmentation, DNA libraries were constructed for each sample prior to hybridisation enrichment and MPS. All 125 SNP markers of the custom enrichment panel were retrieved from twenty-eight samples without recovering any SNP data for negative controls. This SNP dataset is deposited in repository Figshare and can be found at https://doi.org/10.25909/17469443.v1 [38].

### Sex determination and inference of Y-chromosome haplogroup

**Fig 3:**
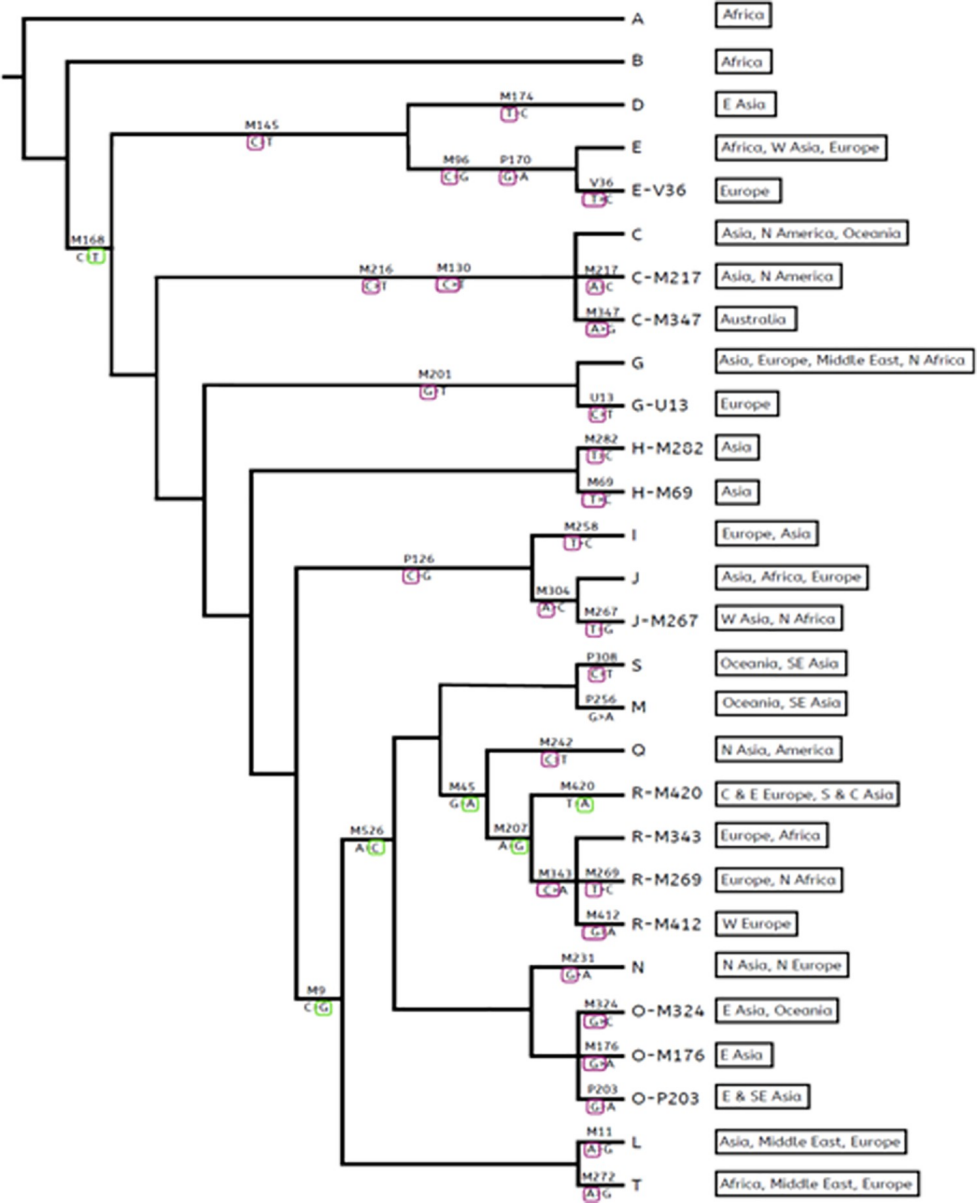
Y-chromosome haplogroup assignment on PhyloTree for sample R1. Haplogroup assigned is R-M420. Derived SNPs: M168(C>T) →M9(C>G) →M526(A>C) →M45(G>A) →M207(A>G) → M420(T>A). Purple and green colour circles show ancestral and derived SNPs, respectively. Names on branches and leaves of tree represents SNP identifiers and haplogroup names, respectively.

All the 35 Y-chromosome SNPs were recovered from all twenty-one male samples. No Y-chr SNPs were called for any of the female samples. Genotype data for all samples has been provided in S1 File. Based on the presence versus absence of Y-chr SNPs all twenty-eight samples were predicted accurately as male or female. Y haplogroup was defined by analyzing SNP data for each sample in which diagnostic ancestral and derived SNPs were observed and assigned in PhyloTree. The output for R1 sample is shown in Fig 3 as an example of the results. In this way haplogroups were assigned to all male samples. Inferred Y-haplogroups reconciled against self-declared lineage for all male samples and results have been summarized in Table 4.

**Table 4:** Summary of Y-chromosome haplogroup results from twenty-one male samples.

Sample	Sex	Self-declared ancestry	Region	Inferred Y-chr haplogroup	Continental Affiliation
**B2**	Male	Asian	Asia	J-M267	West Asia, North Africa
**B4**	Male	Asian	Asia	H-M69	South Asia
**B5**	Male	Asian	Asia	R-M420	C & E Europe, S & C-Asia
**B6**	Male	Asian	Asia	J-M267	West Asia, North Africa
**K4**	Male	Asian	Asia	H-M69	South Asia
**P9**	Male	Asian	Asia	Q	N Asia, America
**P11**	Male	Asian	Asia	H-M69	South Asia
**P12**	Male	Asian	Asia	J and J-M267	Asia, Europe and West Asia
**P14**	Male	Asian	Asia	L	Asia, Middle East, Europe
**PT32**	Male	Asian	Asia	R-M420	C & E Europe, S & C-Asia
**PT34**	Male	Asian	Asia	R-M420	C & E Europe, S & C-Asia
**PT39**	Male	Asian	Asia	R-M420	C & E Europe, S & C-Asia
**PT45**	Male	Asian	Asia	R	C & E Europe, S & C-Asia
**PT50**	Male	Asian	Asia	L	Asia, Middle East, Europe
**G9**	Male	Asian	Asia	R-M420	C & E Europe, S & C-Asia
**Gil9**	Male	Asian	Asia	R-M420	C & E Europe, S & C-Asia
**Gil11**	Male	Asian	Asia	J	Asia
**R1**	Male	Asian	Asia	R-M420	C & E Europe, S & C-Asia
**R2**	Male	Asian	Asia	R	C & E Europe, S & C-Asia
**R3**	Male	Asian	Asia	R-M420	C & E Europe, S & C-Asia
**R5**	Male	Asian	Asia	Q	N-Asia, America

### Estimation of externally visible characteristics

From each of the twenty-eight DNA samples, all phenotype SNPs were obtained successfully. The HIrisPlex correctly predicted eye colour for reported blue and brown eye colours as summarized in Table 5. This data shows highest P-values out of all predicted values for colour of eye and hair and for hair shade. Most probable hair colour is the result of combined information of hair colour and shade probability values. Eye colour was predicted accurately for all of the samples based on the highest p-value except R7 and PT32 for which eye colour predicted as blue instead of brown (actual eye colour observed) giving an overall prediction accuracy of 92.8%. Predictions were consistent with reported hair colour for all samples, using the combined highest p-value approach and step-wise model incorporating probability thresholds for light or dark shade.

**Table 5:** Inferred eye colour and most probable hair colour associated probabilities in terms of P-value for twenty-eight samples with known hair and eye colour using the HIrisplex SNPs in the custom enrichment panel.

Sample	SELF-DECLARED		INFERRED PREDICTIONS			
	Eye Colour	Hair Colour	Eye Colour (P-Value)	Hair Colour (P-Value)	Hair Shade (P-Value)	Most Probable Hair Colour
B2	Brown / Hazel	Brown	Brown (0.547)	Brown (0.629)	Dark (0.826)	Dark Brown
B4	Blue	Brown	Blue (0.510)	Brown (0.720)	Dark (0.720)	Dark Brown
B5	Blue/ Grey	Brown	Blue (0.783)	Brown (0.545)	Light (0.693)	Light Brown
B6	Blue	Brown	Blue (0.649)	Brown (0.560)	Light (0.625)	Light Brown
G9	Blue	Brown	Blue (0.510)	Brown (0.639)	Dark (0.77)	Dark Brown
Gil7	Brown/ Hazel	Brown	Brown (0.547)	Brown (0.741)	Light (0.630)	Light Brown
Gil8	Blue/ Grey	Brown	Blue (0.783)	Brown (0.545)	Light (0.693)	Light Brown
Gil9	Blue	Brown	Blue (0.510)	Brown (0.639)	Dark (0.778)	Dark Brown
Gil11	Blue	Brown	Blue (0.649)	Brown (0.545)	Light (0.693)	Light Brown
K1	Blue	Brown	Blue (0.458)	Brown (0.566)	Dark (0.908)	Dark Brown
K3	Brown	Brown	Brown (0.547)	Brown (0.639)	Dark (0.827)	Dark Brown
K4	Brown	Brown	Brown (0.547)	Brown (0.741)	Light (0.630)	Light Brown
K7	Blue/Grey	Brown	Blue (0.510)	Brown (0.629)	Dark (0.826)	Dark Brown
K8	Blue	Brown	Blue (0.510)	Brown (0.629)	Dark (0.826)	Dark Brown
P9	Blue	Brown	Blue (0.783)	Brown (0.545)	Light (0.693)	Light Brown
P11	Blue/Grey	Brown	Blue (0.783)	Brown (0.483)	Light (0.665)	Light Brown
P12	Blue	Brown	Blue (0.510)	Brown (0.639)	Dark (0.778)	Dark Brown
P14	Blue	Brown	Blue (0.649)	Brown (0.545)	Light (0.693)	Light Brown
PT32	Brown/ Black	Brown	Blue (0.510)	Brown (0.569)	Dark (0.683)	Dark Brown
PT45	Brown	Brown	Brown (0.547)	Brown (0.639)	Dark (0.778)	Dark Brown
PT34	Brown	Brown	Brown (0.547)	Brown (0.569)	Dark (0.683)	Dark Brown
PT39	Brown/ Black	Brown	Brown (0.547)	Brown (0.547)	Dark (0.635)	Dark Brown
PT50	Brown	Brown	Brown (0.547)	Brown (0.629)	Dark (0.826)	Dark Brown
R1	Brown	Brown	Brown (0.547)	Brown (0.629)	Dark (0.826)	Dark Brown
R2	Brown	Brown	Brown (0.547)	Brown (0.629)	Dark (0.826)	Dark Brown
R3	Brown/ Hazel	Brown	Brown (0.547)	Brown (0.629)	Dark (0.826)	Dark Brown
R5	Brown	Brown	Brown (0.547)	Brown (0.629)	Dark (0.826)	Dark Brown
R7	Brown	Brown	Blue (0.510)	Brown (0.660)	Dark (0.613)	Dark Brown

### Assignment of biogeography ancestry

**Fig 4:**
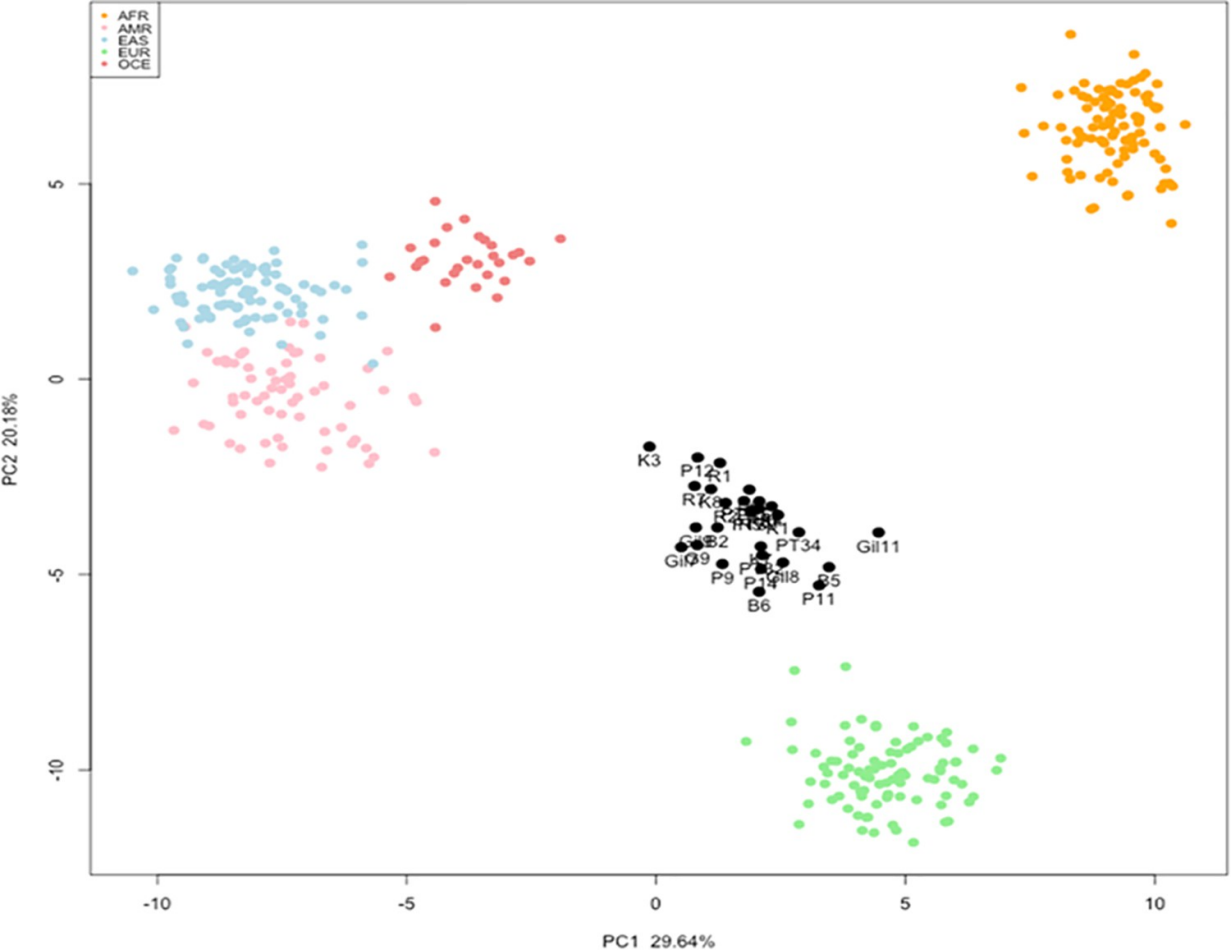
PCA plot for 67 biogeographic ancestry SNPs from 28 Pakistani samples and four global reference populations. Black points represent study samples, also indicated using sample names. Continental reference population samples are shown in yellow (AFR), blue (EAS), green (EUR), pink (AMR), red (OCE) and green (EUR).

From each of the twenty-eight samples, all 67 biogeographic ancestry SNPs were obtained successfully. All likelihood ratios were at least 1 billion times more likely EUR one population over any of the other four populations, with the exception of K3 and P12 (Table 6). In PCA analysis the first PC1 and second PC2 components respectively observed as 29.64% and 20.18% of the total variance. All four reference population samples form separate clusters, although EAS, AMR and OCE are less clearly separated (Fig 4). The 28 Pakistani samples sit intermediate between the EUR and EAS/AMR/OCE clusters in the PCA (Fig 4) but there is no clear separation between samples from different ethnic groups. Biogeographic ancestry predictions are inconsistent with self-declared ancestry as per Snipper results due to limitation in accurately accounting for admixture by the tool and the absence of SNPs in the panel that can distinguish South Asian ancestry from European or East Asian. Therefore, use of some additional SNPs especially for differentiation of South Asian populations from those to the west and east will help in differentiating between these populations. Moreover, the reference dataset used for comparison included 89 individuals from EAS population which were JPT: Japanese in Tokyo, hence it is the only representation for EAS group. Inclusion of distinct individual’s genotype data from various countries and ethnic groups of Asia especially Pakistan and neighbouring countries for representation of EAS population group in reference dataset can also improve final predicted results and clear biogeography-ancestry estimation.

**Table 6:** Inferred biogeographic ancestry using snipper for male and female samples under study.

Sample	Self-declared ancestry	Region	Lowest and Highest Likelihood from Snipper
**B2**	Asian	Asia	10E+9 times more likely to be EUR than EAS and 10E+9 times more likely to be EUR than AMR
**B4**	Asian	Asia	10E+9 times more likely to be EUR than EAS and 10E+9 times more likely to be EUR than AFR
**B5**	Asian	Asia	10E+9 times more likely to be EUR than AFR and 10E+9 times more likely to be EUR than EAS
**B6**	Asian	Asia	10E+9 times more likely to be EUR than EAS and 10E+9 times more likely to be EUR than AMR
**G9**	Asian	Asia	10E+9 times more likely to be EUR than AMR and 10E+9 times more likely to be EUR than EAS
**Gil7**	Asian	Asia	10E+9 times more likely to be EUR than AMR and 10E+9 times more likely to be EUR than EAS
**Gil8**	Asian	Asia	10E+9 times more likely to be EUR than AMR and 10E+9 times more likely to be EUR than EAS
**Gil9**	Asian	Asia	10E+9 times more likely to be EUR than AMR and 10E+9 times more likely to be EUR than EAS
**Gil11**	Asian	Asia	10E+9 times more likely to be EUR than AFR and 10E+9 times more likely to be EUR than EAS
**K1**	Asian	Asia	10E+9 times more likely to be EUR than EAS and 10E+9 times more likely to be EUR than AFR
**K3**	Asian	Asia	2,236 times more likely to be EUR than EAS and 10E+9 times more likely to be EUR than AMR
**K4**	Asian	Asia	10E+9 times more likely to be EUR than EAS and 10E+9 times more likely to be EUR than AMR
**K7**	Asian	Asia	10E+9 times more likely to be EUR than EAS and 10E+9 times more likely to be EUR than AMR
**K8**	Asian	Asia	10E+9 times more likely to be EUR than EAS and 10E+9 times more likely to be EUR than AMR
**P9**	Asian	Asia	10E+9 times more likely to be EUR than EAS and 10E+9 times more likely to be EUR than AMR
**P11**	Asian	Asia	10E+9 times more likely to be EUR than AMR and 10E+9 times more likely to be EUR than EAS
**P12**	Asian	Asia	831 times more likely to be EUR than AMR, and 70,915,529 times more likely to be EUR than EAS
**P14**	Asian	Asia	10E+9 times more likely to be EUR than AMR and 10E+9 times more likely to be EUR than EAS
**PT32**	Asian	Asia	10E+9 times more likely to be EUR than EAS and 10E+9 times more likely to be EUR than AMR
**PT34**	Asian	Asia	10E+9 times more likely to be EUR than AFR and 10E+9 times more likely to be EUR than EAS
**PT39**	Asian	Asia	10E+9 times more likely to be EUR than AMR and 10E+9 times more likely to be EUR than EAS
**PT45**	Asian	Asia	10E+9 times more likely to be EUR than EAS and 10E+9 times more likely to be EUR than AMR
**PT50**	Asian	Asia	10E+9 times more likely to be EUR than EAS and 10E+9 times more likely to be EUR than AMR
**R1**	Asian	Asia	10E+9 times more likely to be EUR than EAS and 10E+9 times more likely to be EUR than AMR
**R2**	Asian	Asia	10E+9 times more likely to be EUR than EAS and 10E+9 times more likely to be EUR than AMR
**R3**	Asian	Asia	10E+9 times more likely to be EUR than EAS and 10E+9 times more likely to be EUR than AMR
**R5**	Asian	Asia	10E+9 times more likely to be EUR than EAS and 10E+9 times more likely to be EUR than AMR
**R7**	Asian	Asia	10E+9 times more likely to be EUR than EAS and 10E+9 times more likely to be EUR than AMR

## Discussion

Human identification is a complex process that is important for social and legal reasons. In forensic investigations, MPS can enhance the potential of human identification and help resolve mixture complexities [1]. For SNP typing of samples in forensic investigations, there are many recent MPS approaches that show promise for generating information for multiple markers in a single process [8, 9]. The latest hybridisation enrichment strategies for MPS analysis of DNA samples have enhanced opportunities to obtain volumes of genetic data for forensic intelligence and identification purposes [5, 6, 39].

Predicting physical characteristics from DNA as a biological source termed as forensic DNA phenotyping has gained popularity within forensics due to the potential intelligence information it can provide [40, 41]. This facilitates sensitive investigations in which conventional DNA profiling fails or does not provides useful outcomes. There are already developed and forensically authenticated systems consisting of specific markers designated for specific tasks. One example is the IrisPlex system which is a dedicated DNA test for eye colour prediction [42]. Likewise, HIrisPlex as used in the present study combines the SNPs for both eye and hair colour prediction in its system [43]. We analysed samples from different ethnic groups of Pakistan to represent different hair and eye colours. It has been investigated this way that the inclusion of the phenotype SNPs with the ancestry and Y-chr SNPs using a hybridisation enrichment technology gives results that are consistent with known phenotype. Brown and blue eye colours were predicted accurately in all cases in research by [4], however intermediate eye colours remained problematic to predict, giving an overall 83% prediction accuracy of the SNPs to infer eye colour. Interestingly, when excluding the intermediate eye colour category (sometimes explored due to the potential inaccuracies in predicting intermediate eye colour against observed eye colour) [4], the prediction accuracy increases to 92% when grouping individuals into ‘brown’ and ‘not brown’ eye colour categories. Given that pigmentation in eye colour is a complex trait which can be subjective to report [44], and that intermediate eye colour has demonstrated a lower prediction accuracy than other eye colours in previous studies [4, 45, 46], this result is not unexpected. For samples in the present study, a 100% prediction accuracy was achieved across the twenty- eight samples for hair colour. Predictions were consistent with reported hair colour for all samples, using the combined highest p-value approach and step-wise model incorporating probability thresholds for light or dark shade. Eye colour predicted accurately for all of the samples based on the highest p-value except R7 and PT32 for which eye colour predicted as blue instead of brown (actual eye colour) giving prediction accuracy of 92.8%. Again, previous studies have documented inaccuracies with predicting hair colour phenotypes (down to a 73% prediction accuracy on average), particularly with blond and brown categories [4, 46]. For both hair and eye colour, the prediction accuracy shown in this study is consistent with previous error rates established in earlier studies of the HIrisPlex SNP panel [4, 43]. Since the design and execution of the panel used in the present research, a latest HIrisPlex panel has been published, called HIrisPlex-S assay which includes additional 17 SNP markers in pigmentation genes which provides additional facilitation of inferring skin colour [47, 48]. As a further consideration, these SNPs could easily be incorporated in to the customized enrichment panel as per needs which can serve as a further intelligence tool. Nonetheless, this study has demonstrated the successful use of the HIrisPlex panel in a hybridisation enrichment approach for forensic analysis and may help to further support ancestry estimations when used in conjunction with the ancestry informative SNPs in the custom panel. Currently, the HIrisPlex model includes test data only from European populations [4]. Understanding how different populations may influence the prediction model and therefore the success rate could be improved by including reference samples from multiple non-European populations as from present study.

All 67 biogeographic-ancestry SNPs were successfully retrieved from all twenty-eight samples under study. Comparative study for the target sample’s SNP genotype data versus available reference population data showed that all likelihood ratios were at least 1 billion times more likely one population over any of the other four populations, with the exception of samples K3 and P12. Use of some additional SNPs especially enlightening for pairwise differentiation of east and south Asia’s populations will boost the ability of the panel to differentiate between these populations. Moreover, the reference dataset used for comparison included 89 individuals from EAS population which were JPT: Japanese in Tokyo, hence it is the only representation for EAS group. Inclusion of population genotype data from various countries and ethnic groups of Asia especially Pakistan and neighbouring countries for representation of east and south Asian population groups in the reference dataset could improve final predicted results and clearer biogeography-ancestry estimation. Snipper has also limitation in accurately accounting for admixture, hence it can be concluded that samples under study showed an admixture between EAS and EUR ancestry.

Research has been dedicated for many years on the human Y- chromosome and its variation analysis especially targeting YSNPs. This effort resulted in establishing a well-defined Y chromosome phylogeny. The rise of MPS approaches in recent times is facilitating the discovery of new YSNPs which are in turn increasing resolution to discriminate between closely related Y-haplotypes. The Y-chromosome being haploid and largely non-recombining in nature, is widely used as a marker in many disciplines including forensics research [49, 50], exploring structure of Y chromosome [51], and population based studies [52, 53]. The Y-SNPs in the custom enrichment panel were able to predict Y-chr haplogroups for all male samples with no conflicting haplogroup classifications. No Y-chr SNP data was recovered from any of the female samples, which also indicates the capability for this method as an indication of sex. For all twenty-one male samples under study, haplogroup classifications and their associated most likely geographic affiliations were reconciled with reported self-declared ancestry. Self-declared ancestry and region of samples under study have been affiliated well with inferred one i.e. Asian as all samples belong to local ethnic populations of Pakistan. The panel has successfully determined informative Y-chr haplogroups and sub-haplogroups and can be considered a suitable tool for exploring the paternal lineage of male samples.

Whole genome sequencing is the only method that allows the simultaneous detection of all types of variations within a genome. In a single assay, a wide range of applications can be examined with the downstream analysis providing information about targets that need close examination. But as reads with bad quality were dropped prior to analysis, and whole genome approach yields less coverage in comparison to targeted approach which sequence only loci of interests. Targeted approach identifies those variants that get skipped as a result of whole genome sequencing [54]. It eliminates redundant and unnecessary genetic variations that can lead to distraction from direct interpretation. It is cost and time effective option, especially when a large number of target samples are under study like present research [54].

## Conclusion

In an attempt to analyze various marker types together in one analytical workflow for forensic human intelligence information, a novel customisable hybridisation enrichment forensic intelligence panel has been used in the present research which provided new avenues and opened many insights to forensic human identification. This panel facilitates a technical approach that permits the possibility of using customisable SNP marker sets relevant to the question under study for hybridisation enrichment prior to MPS. The panel has distinguished biogeographic ancestry of each study sample between five major continental populations by successfully targeting 67 ancestry informative markers. Y-chr SNP analysis helped in sex determination and assigning haplogroups. Retrieval and analysis of externally visible characteristics (EVCs) such as eyes and hair colour has been achieved by targeting genomes with 23 phenotype markers and HIrisPlex phenotyping tool results match well with previously established success rates. SNPs that are helpful for prediction of more external physical traits, SNPs for biogeography lineage prediction or any additional SNPs that can facilitate in forensic research can be used as individual or in combined customisable panel to facilitate advanced outcomes. An example is recent introduction of HIrisPlex-S system that covers additional 17 SNPs in its panel that facilitates prediction of skin colour along with hair and eyes. The overarching objective of the present research was to explore and use the latest techniques to increase the likelihood of drawing inferences regarding phenotype and lineage from modern human DNA for forensic investigations in Pakistan.

## Supporting information

S1 File Summary of 35 Y-chromosome SNP genotype data for all male samples. (DOCX)

S2 File HIrisPlex input file data of 23 phenotypic marker’s genotype for samples under study. (DOCX)

S3 File Input file in.xlsx format for Snipper tool. Dataset shows genotypes of study and reference samples for 67 biogeographic SNPs. First row indicates number of samples, Total number of SNPs, number of populations, rs-IDs for SNPs, respectively column wise.
(DOCX)
