# Supplementary material for: Unveiling forensically relevant biogeographic, phenotype and Y-chromosome SNP variation in Pakistani ethnic groups using a customized hybridisation enrichment forensic intelligence panel
Source: PLoS One. 2022 Feb 17;17(2):e0264125. doi: 10.1371/journal.pone.0264125 (PMC8853543; doi:10.1371/journal.pone.0264125)
Supplement: S1 File — (DOCX) [file pone.0264125.s001.docx]

| **Hg19 location** | **rs ID#** | **Y-SNP code** | **Ancestral SNP** | **Derived SNP** | **Inferred haplogroup for derived SNP** | **B2** | **B4** | **B5** | **B6** | **K4** | **P9** | **P11** | **P12** | **P14** | **PT32** |
| --- | --- | --- | --- | --- | --- | --- | --- | --- | --- | --- | --- | --- | --- | --- | --- |
| 2655180 | rs11575897 | M176 | **G** | A | O-M176 | G | G | G | G | G | G | G | G | G | G |
| 2734854 | rs35284970 | M130 | **C** | T | C | C | C | C | C | C | C | C | C | C | C |
| 2821786 | rs13447361 | M324 | **G** | C | O-M324 | G | G | G | G | G | G | G | G | G | G |
| 2877479 | rs868363758 | M347 | **A** | G | C-M347 | A | A | A | A | A | A | A | A | A | A |
| 2887824 | rs9786184 | M343 | **C** | A | R | C | C | C | C | C | C | C | C | C | C |
| 6814246 | rs371443469 | V36 | **T** | C | E-36 | T | T | T | T | T | T | T | T | T | T |
| 8502236 | rs9786140 | M412 | **G** | A | R-M412 | G | G | G | G | G | G | G | G | G | G |
| 8685230 | n/a | P256 | **G** | A | M>S | G | G | G | G | G | G | G | G | G | G |
| 14698928 | rs9786706 | U13 | **C** | T | G-U13 | C | C | C | C | C | C | C | C | C | C |
| 14813991 | rs2032595 | M168 | **C** | T | not AB | T | T | T | T | T | T | T | T | T | T |
| 14954280 | rs2032602 | M174 | **T** | C | D | T | T | T | T | T | T | T | T | T | T |
| 15018582 | rs8179021 | M242 | **C** | T | Q | C | C | C | C | C | T | C | C | C | C |
| 15021522 | rs9786025 | P170 | **G** | A | E | G | G | G | G | G | G | G | G | G | G |
| 15023364 | rs9341301 | M258 | **T** | C | I | T | T | T | T | T | T | T | T | T | T |
| 15027529 | rs2032636 | M201 | **G** | T | G | G | G | G | G | G | G | G | G | G | G |
| 15409573 | n/a | P308 | **C** | T | S | C | C | C | C | C | C | C | C | C | C |
| 15437333 | rs2032668 | M217 | **A** | C | C-M217 | A | A | A | A | A | A | A | A | A | A |
| 15437564 | rs2032666 | M216 | **C** | T | C | C | C | C | C | C | C | C | C | C | C |
| 15469724 | rs9341278 | M231 | **G** | A | N | G | G | G | G | G | G | G | G | G | G |
| 15581983 | rs2032658 | M207 | **A** | G | R | A | A | G | A | A | A | A | A | A | G |
| 21225770 | rs17250163 | P126 | **C** | G | IJ | C | C | C | G | C | C | C | G | C | C |
| 21717208 | rs3848982 | M145 | **C** | T | DE | C | C | C | C | C | C | C | C | C | C |
| 21730257 | rs3900 | M9 | **C** | G | L>T | C | C | G | G | C | G | C | C | G | G |
| 21730647 | rs3902 | M11 | **A** | G | L | A | A | A | A | A | A | A | A | G | A |
| 21764431 | rs13447371 | M282 | **T** | C | H-M282 | T | T | T | T | T | T | T | T | T | T |
| 21778998 | rs9306841 | M96 | **C** | G | E | C | C | C | C | C | C | C | C | C | C |
| 21867787 | rs2032631 | M45 | **G** | A | QR | G | G | A | G | G | A | G | G | G | A |
| 21894058 | rs2032673 | M69 | **T** | C | H-M69 | T | C | T | T | C | T | C | T | T | T |
| 22738775 | rs9341308 | M272 | **A** | G | T | A | A | A | A | A | A | A | A | A | A |
| 22739367 | rs9786153 | M269 | **T** | C | R-M269 | T | T | T | T | T | T | T | T | T | T |
| 22741818 | rs9341313 | M267 | **T** | G | J-M267 | G | T | T | G | T | T | T | T | T | T |
| 22749853 | rs13447352 | M304 | **A** | C | J | C | A | A | C | A | A | A | C | A | A |
| 22750951 | rs13447354 | P203 | **G** | A | O-P203 | G | G | G | G | G | G | G | G | G | G |
| 23473201 | rs17250535 | M420 | **T** | A | R-M420 | T | T | A | T | T | T | T | T | T | A |
| 23550924 | rs2033003 | M526 | **A** | C | M>S | A | A | C | A | A | C | A | A | A | C |
|  |  |  |  |  |  |  |  |  |  |  |  |  |  |  |  |
|  |  |  |  |  | Haplogroup>>> | J-M267 | H-M69 | R-M420 | J-M267 | H-M69 | Q | H-M69 | J and J-M267 | L | R-M420 |

| **Hg19 location** | **rs ID#** | **Y-SNP code** | **Ancestral** | **Derived** | **Inferred haplogroup for derived SNP** | **PT34** | **PT39** | **PT45** | **PT50** | **G9** | **Gil9** | **Gil11** | **R1** | **R2** | **R3** | **R5** |
| --- | --- | --- | --- | --- | --- | --- | --- | --- | --- | --- | --- | --- | --- | --- | --- | --- |
| 2655180 | rs11575897 | M176 | **G** | A | O-M176 | G | G | G | G | G | G | G | G | G | G | G |
| 2734854 | rs35284970 | M130 | **C** | T | C | C |  | C | C | C | C | C | C | C | C | C |
| 2821786 | rs13447361 | M324 | **G** | C | O-M324 | G | G | G | G | G | G | G | G | G | G | G |
| 2877479 | rs868363758 | M347 | **A** | G | C-M347 | A | A | A | A | A | A | A | A | A | A | A |
| 2887824 | rs9786184 | M343 | **C** | A | R | C | C | C | C | C | C | C | C | C | C | C |
| 6814246 | rs371443469 | V36 | **T** | C | E-36 | T | T | T | T | T | T | T | T | T | T | T |
| 8502236 | rs9786140 | M412 | **G** | A | R-M412 | G |  | G | G | G | G | G | G | G | G | G |
| 8685230 | n/a | P256 | **G** | A | M>S | G | G | G | G | G | G | G | G | G | G | G |
| 14698928 | rs9786706 | U13 | **C** | T | G-U13 | C | C | C | C | C | C | C | C | C | C | C |
| 14813991 | rs2032595 | M168 | **C** | T | not AB | T | T | T | T | T | T | T | T | T | T | T |
| 14954280 | rs2032602 | M174 | **T** | C | D | T | T | T | T | T | T | T | T | T | T | T |
| 15018582 | rs8179021 | M242 | **C** | T | Q | C | C | C | C | C | C | C | C | C | C | T |
| 15021522 | rs9786025 | P170 | **G** | A | E | G | G | G | G | G | G | G | G | G | G | G |
| 15023364 | rs9341301 | M258 | **T** | C | I | T | T | T | T | T | T | T | T | T |  | T |
| 15027529 | rs2032636 | M201 | **G** | T | G | G |  | G | G | G | G | G | G | G | G | G |
| 15409573 | n/a | P308 | **C** | T | S | C | C | C | C | C | C | C | C | C | C | C |
| 15437333 | rs2032668 | M217 | **A** | C | C-M217 | A | A | A | A | A | A | A | A | A | A | A |
| 15437564 | rs2032666 | M216 | **C** | T | C | C | C | C | C | C | C | C | C | C | T | C |
| 15469724 | rs9341278 | M231 | **G** | A | N | G |  | G | G | G | G | G | G | G | G | G |
| 15581983 | rs2032658 | M207 | **A** | G | R | G | G | G | A | G | G | A | G | G | G |  |
| 21225770 | rs17250163 | P126 | **C** | G | IJ | C | C | C | C | C | C | G | C | C | C | C |
| 21717208 | rs3848982 | M145 | **C** | T | DE | C | C | C | C | C | C | C | C | C | C | C |
| 21730257 | rs3900 | M9 | **C** | G | L>T | G |  | G | G | G | G | G | G | G | G | G |
| 21730647 | rs3902 | M11 | **A** | G | L | A | A | A | G | A | A | A | A | A | A | A |
| 21764431 | rs13447371 | M282 | **T** | C | H-M282 | T | T | T | T | T | T | T | T | T | T | T |
| 21778998 | rs9306841 | M96 | **C** | G | E | C |  | C | C | C | C | C | C | C | C | C |
| 21867787 | rs2032631 | M45 | **G** | A | QR | A |  |  | G | A | A | G | A | A | G | A |
| 21894058 | rs2032673 | M69 | **T** | C | H-M69 | T | T | T | T | T | T | T | T | T | T | T |
| 22738775 | rs9341308 | M272 | **A** | G | T | A | A | A | A | A | A | A | A | A | A | A |
| 22739367 | rs9786153 | M269 | **T** | C | R-M269 | T |  | T | T | T | T | T | T | T |  | T |
| 22741818 | rs9341313 | M267 | **T** | G | J-M267 | T | T | T | T | T | T | T | T | T | T | T |
| 22749853 | rs13447352 | M304 | **A** | C | J | A |  |  | A | A | A | C | A | A |  | A |
| 22750951 | rs13447354 | P203 | **G** | A | O-P203 | G | G | G | G | G | G | G | G | G | G | G |
| 23473201 | rs17250535 | M420 | **T** | A | R-M420 | A | A | T | T | A | A | T | A | T | A | T |
| 23550924 | rs2033003 | M526 | **A** | C | M>S | C |  | C | A | C | C | A | C | C | A | C |
|  |  |  |  |  |  |  |  |  |  |  |  |  |  |  |  |  |
|  |  |  |  |  | Haplogroup>>> | R-M420 | R-M420 | R | L | R-M420 | R-M420 | J | R-M420 | R | R-M420 | Q |
