# Supplementary material for: Unveiling forensically relevant biogeographic, phenotype and Y-chromosome SNP variation in Pakistani ethnic groups using a customized hybridisation enrichment forensic intelligence panel
Source: PLoS One. 2022 Feb 17;17(2):e0264125. doi: 10.1371/journal.pone.0264125 (PMC8853543; doi:10.1371/journal.pone.0264125)
Supplement: S2 File — (DOCX) [file pone.0264125.s002.docx]

| **sampleid** | **rs16891982_C** | **rs28777_C** | **rs12203592_T** | **rs4959270_A** | **rs683_G** | **rs1042602_T** | **rs1393350_T** | **rs12821256_G** |
| --- | --- | --- | --- | --- | --- | --- | --- | --- |
| B2 | 2 | 2 | 0 | 0 | 0 | 0 | 0 | 0 |
| B4 | 2 | 2 | 0 | 1 | 0 | 0 | 0 | 0 |
| B5 | 1 | 1 | 0 | 1 | 0 | 0 | 0 | 0 |
| B6 | 1 | 1 | 0 | 0 | 0 | 0 | 0 | 0 |
| G9 | 2 | 2 | 0 | 1 | 0 | 0 | 0 | 0 |
| Gil7 | 2 | 2 | 0 | 0 | 0 | 0 | 0 | 0 |
| Gil8 | 1 | 1 | 0 | 1 | 0 | 0 | 0 | 0 |
| K7 | 2 | 2 | 0 | 0 | 0 | 0 | 0 | 0 |
| P14 | 1 | 1 | 0 | 1 | 0 | 0 | 0 | 0 |
| PT32 | 2 | 1 | 0 | 0 | 0 | 0 | 0 | 0 |
| PT50 | 2 | 2 | 0 | 0 | 0 | 0 | 0 | 0 |
| Gil11 | 1 | 1 | 0 | 1 | 0 | 0 | 0 | 0 |
| Gil9 | 2 | 2 | 0 | 1 | 0 | 0 | 0 | 0 |
| P9 | 1 | 1 | 0 | 1 | 0 | 0 | 0 | 0 |
| R5 | 2 | 2 | 0 | 0 | 0 | 0 | 0 | 0 |
| R7 | 2 | 1 | 0 | 0 | 0 | 0 | 0 | 0 |
| K1 | 2 | 1 | 1 | 1 | 0 | 0 | 0 | 0 |
| K3 | 2 | 2 | 0 | NA | NA | 0 | 0 | 0 |
| K4 | 2 | 2 | 0 | 0 | 0 | 0 | 0 | 0 |
| K7 | 2 | 2 | 0 | 0 | 0 | 0 | 0 | 0 |
| P11 | 1 | 1 | 0 | 1 | 0 | 0 | 0 | 0 |
| P12 | 2 | 2 | 0 | 1 | 0 | 0 | 0 | 0 |
| PT34 | 2 | 1 | 0 | 0 | 0 | 0 | 0 | 0 |
| PT39 | 2 | 1 | 0 | 0 | 0 | 0 | 0 | 0 |
| R1 | 2 | 2 | 0 | 0 | 0 | 0 | 0 | 0 |
| R2 | 2 | 2 | 0 | 0 | 0 | 0 | 0 | 0 |
| K8 | 2 | 2 | 0 | 0 | 0 | 0 | 0 | 0 |
| PT45 | 2 | 2 | 0 | 1 | 0 | 0 | 0 | 0 |
| R3 | 2 | 2 | 0 | 0 | 0 | 0 | 0 | 0 |

| **sampleid** | **rs12896399_T** | **rs2402130_G** | **rs1800407_A** | **rs12913832_T** | **rs1805005_T** | **rs1805006_A** | **rs885479_T** | **rs11547464_A** |
| --- | --- | --- | --- | --- | --- | --- | --- | --- |
| B2 | 0 | 1 | 0 | 0 | 0 | 0 | 0 | 0 |
| B4 | 1 | 1 | 0 | 0 | 1 | 0 | 0 | 0 |
| B5 | 1 | 1 | 0 | 0 | 0 | 0 | 0 | 0 |
| B6 | 0 | 1 | 0 | 0 | 0 | 0 | 0 | 0 |
| G9 | 1 | 1 | 0 | 0 | 0 | 0 | 0 | 0 |
| Gil7 | 0 | 1 | 0 | 0 | 0 | 0 | 0 | 0 |
| Gil8 | 1 | 1 | 0 | 0 | 0 | 0 | 0 | 0 |
| K7 | 1 | 1 | 0 | 0 | 0 | 0 | 0 | 0 |
| P14 | 0 | 1 | 0 | 0 | 0 | 0 | 0 | 0 |
| PT32 | 1 | 1 | 0 | 0 | 0 | 0 | 0 | 0 |
| PT50 | 0 | 1 | 0 | 0 | 0 | 0 | 0 | 0 |
| Gil11 | 0 | 1 | 0 | 0 | 0 | 0 | 0 | 0 |
| Gil9 | 1 | 1 | 0 | 0 | 0 | 0 | 0 | 0 |
| P9 | 1 | 1 | 0 | 0 | 0 | 0 | 0 | 0 |
| R5 | 0 | 1 | 0 | 0 | 0 | 0 | 0 | 0 |
| R7 | 1 | 1 | 0 | 0 | 1 | 0 | 0 | 0 |
| K1 | 0 | 1 | 0 | 0 | 0 | 0 | 0 | 0 |
| K3 | 0 | 1 | 0 | 0 | 0 | 0 | 0 | 0 |
| K4 | 0 | 1 | 0 | 0 | 0 | 0 | 0 | 0 |
| K7 | 1 | 1 | 0 | 0 | 0 | 0 | 0 | 0 |
| P11 | 1 | 1 | 0 | 0 | 0 | 0 | 0 | 0 |
| P12 | 1 | 1 | 0 | 0 | 0 | 0 | 0 | 0 |
| PT34 | 0 | 1 | 0 | 0 | 0 | 0 | 0 | 0 |
| PT39 | 0 | NA | 0 | 0 | 0 | 0 | 0 | 0 |
| R1 | 0 | 1 | 0 | 0 | 0 | 0 | 0 | 0 |
| R2 | 0 | 1 | 0 | 0 | 0 | 0 | 0 | 0 |
| K8 | 1 | 1 | 0 | 0 | 0 | 0 | 0 | 0 |
| PT45 | 0 | 1 | 0 | 0 | 0 | 0 | 0 | 0 |
| R3 | 0 | 1 | 0 | 0 | 0 | 0 | 0 | 0 |

| **sampleid** | **rs1805007_T** | **rs201326893_A** | **rs1110400_C** | **rs1805008_T** | **rs2228479_A** | **rs1805009_C** | **rs2378249_C** |
| --- | --- | --- | --- | --- | --- | --- | --- |
| B2 | 0 | 0 | 0 | 0 | 0 | 0 | 0 |
| B4 | 0 | 0 | 0 | 0 | 0 | 0 | 0 |
| B5 | 0 | 0 | 0 | 0 | 0 | 0 | 0 |
| B6 | 0 | 0 | 0 | 0 | 0 | 0 | 0 |
| G9 | 0 | 0 | 0 | 0 | 0 | 0 | 0 |
| Gil7 | 1 | 0 | 0 | 0 | 0 | 0 | 0 |
| Gil8 | 0 | 0 | 0 | 0 | 0 | 0 | 0 |
| K7 | 0 | 0 | 0 | 0 | 0 | 0 | 0 |
| P14 | 0 | 0 | 0 | 0 | 0 | 0 | 0 |
| PT32 | 0 | 0 | 0 | 0 | 0 | 0 | 0 |
| PT50 | 0 | 0 | 0 | 0 | 0 | 0 | 0 |
| Gil11 | 0 | 0 | 0 | 0 | 0 | 0 | 0 |
| Gil9 | 0 | 0 | 0 | 0 | 0 | 0 | 0 |
| P9 | 0 | 0 | 0 | 0 | 0 | 0 | 0 |
| R5 | 0 | 0 | 0 | 0 | 0 | 0 | 0 |
| R7 | 0 | 0 | 0 | 0 | 0 | 0 | 0 |
| K1 | 0 | 0 | 0 | 0 | 0 | 0 | 0 |
| K3 | 0 | 0 | 0 | 0 | 0 | 0 | NA |
| K4 | 1 | 0 | 0 | 0 | 0 | 0 | 0 |
| K7 | 0 | 0 | 0 | 0 | 0 | 0 | 0 |
| P11 | 0 | 0 | 0 | 0 | 1 | 0 | 0 |
| P12 | 0 | 0 | 0 | 0 | 0 | 0 | 0 |
| PT34 | 0 | 0 | 0 | 0 | 0 | 0 | 0 |
| PT39 | 0 | 0 | 0 | 0 | NA | 0 | 0 |
| R1 | 0 | 0 | 0 | 0 | 0 | 0 | 0 |
| R2 | 0 | 0 | 0 | 0 | 0 | 0 | 0 |
| K8 | 0 | 0 | 0 | 0 | 0 | 0 | 0 |
| PT45 | 0 | 0 | 0 | 0 | 0 | 0 | 0 |
| R3 | 0 | 0 | 0 | 0 | 0 | 0 | 0 |

0: If input allele is not present, 1: one allele is present, heterozygote, 2: homozygote i.e. the only allele present is the input allele, NA: missing SNP
