# Supplementary material for: Unveiling forensically relevant biogeographic, phenotype and Y-chromosome SNP variation in Pakistani ethnic groups using a customized hybridisation enrichment forensic intelligence panel
Source: PLoS One. 2022 Feb 17;17(2):e0264125. doi: 10.1371/journal.pone.0264125 (PMC8853543; doi:10.1371/journal.pone.0264125)
Supplement: S3 File — Dataset shows genotypes of study and reference samples for 67 biogeographic SNPs. First row indicates number of samples, Total number of SNPs, number of populations, rs-IDs for SNPs, respectively column wise. (DOCX) [file pone.0264125.s003.docx]

| **396** | **67** | **11** | **rs12142199** | **rs647325** | **rs2139931** | **rs12402499** | **rs2814778** | **rs4657449** | **rs2184030** |
| --- | --- | --- | --- | --- | --- | --- | --- | --- | --- |
| 1 | AFR | HG02922 | GG | GG | AA | GG | CC | GG | GA |
| 2 | AFR | HG02923 | GG | GA | AA | GG | CC | GG | GG |
| 3 | AFR | HG02938 | GG | GA | AA | GG | CC | GG | GC |
| 4 | AFR | HG02941 | GG | GG | AA | GG | CC | GG | CC |
| 5 | AFR | HG02943 | GG | GG | AA | GG | CC | GG | GA |
| 6 | AFR | HG02944 | GG | GG | AA | GG | CC | GG | GG |
| 7 | AFR | HG02946 | AG | GG | AA | GG | CC | GG | GG |
| 8 | AFR | HG02947 | GG | AG | AG | GG | CC | GG | CG |
| 9 | AFR | HG02952 | GG | GG | AA | GG | CC | GG | GC |
| 10 | AFR | HG02953 | GG | GG | AA | GG | CC | GG | AC |
| 11 | AFR | HG02968 | GG | GG | AA | GG | CC | GG | GG |
| 12 | AFR | HG02970 | GG | GG | AA | GG | CC | GG | CG |
| 13 | AFR | HG02971 | GG | AA | AA | GG | CC | GG | GC |
| 14 | AFR | HG02973 | GG | GG | AA | GG | CC | GG | GA |
| 15 | AFR | HG02974 | GG | AG | AA | GG | CC | GG | GG |
| 16 | AFR | HG02976 | GG | GG | AA | GG | CC | AG | GG |
| 17 | AFR | HG02977 | GG | AG | AA | GG | CC | GG | GG |
| 18 | AFR | HG02979 | GG | GG | AG | GG | CC | GG | GG |
| 19 | AFR | HG02981 | GG | AA | AA | GG | CC | GG | GA |
| 20 | AFR | HG03099 | GG | GG | AA | GG | CC | GG | GC |
| 21 | AFR | HG03100 | GG | AA | AA | GG | CC | GG | AA |
| 22 | AFR | HG03103 | GG | AA | AA | GG | CC | GG | GA |
| 23 | AFR | HG03105 | AG | GA | AA | GG | CC | GG | GG |
| 24 | AFR | HG03108 | GG | AG | AA | GG | CC | GG | GG |
| 25 | AFR | HG03109 | GG | GG | AA | GG | CC | GG | GG |
| 26 | AFR | HG03111 | GG | GG | AA | GG | CC | GG | AC |
| 27 | AFR | HG03112 | GG | GA | AA | GG | CC | AG | GG |
| 28 | AFR | HG03114 | GG | GG | AA | GG | CC | GG | GC |
| 29 | AFR | HG03115 | GG | GG | AA | GG | CC | GG | GG |
| 30 | AFR | HG03117 | GG | AG | AA | GG | CC | GG | AG |
| 31 | AFR | HG03118 | GG | AG | AA | GG | CC | GG | GG |
| 32 | AFR | HG03120 | GG | AG | AA | GG | CC | GG | GA |
| 33 | AFR | HG03121 | GG | AA | AA | GG | CC | AG | GC |
| 34 | AFR | HG03123 | GG | AG | AA | GG | CC | GG | AG |
| 35 | AFR | HG03124 | GG | GG | AA | GG | CC | GG | CA |
| 36 | AFR | HG03126 | GG | GG | AA | GG | CC | GG | CG |
| 37 | AFR | HG03127 | GG | GA | AA | GG | CC | GG | CG |
| 38 | AFR | HG03129 | GG | AG | AA | GG | CC | AG | AG |
| 39 | AFR | HG03130 | GG | GG | AA | GG | CC | AG | AG |
| 40 | AFR | HG03132 | GG | GA | AA | GG | CC | GG | CC |
| 41 | AFR | HG03133 | GG | GA | AA | GG | CC | GG | GG |
| 42 | AFR | HG03135 | AG | AG | AA | GG | CC | GG | GA |
| 43 | AFR | HG03136 | GG | GA | AA | GG | CC | AG | GG |
| 44 | AFR | HG03139 | GG | AG | AA | GG | CC | GG | CG |
| 45 | AFR | HG03157 | GG | AA | AA | GG | CC | GG | CG |
| 46 | AFR | HG03159 | GG | GG | AG | GG | CC | AG | GG |
| 47 | AFR | HG03160 | GG | GG | AA | GG | CC | GG | CG |
| 48 | AFR | HG03162 | GG | GG | AA | GG | CC | GG | GG |
| 49 | AFR | HG03163 | GG | GG | AA | GG | CC | GG | GG |
| 50 | AFR | HG03166 | GG | GG | AA | GG | CC | GG | AG |
| 51 | AFR | HG03168 | GG | GG | AA | GG | CC | GG | GC |
| 52 | AFR | HG03169 | GG | GA | AG | GG | CC | AG | GC |
| 53 | AFR | HG03172 | GG | GA | AA | GG | CC | AG | GG |
| 54 | AFR | HG03175 | GG | GG | AA | GG | CC | GG | CA |
| 55 | AFR | HG03189 | GG | AA | AA | GG | CC | GG | GG |
| 56 | AFR | HG03190 | GG | GG | AA | GG | CC | GG | GG |
| 57 | AFR | HG03193 | GG | GG | AA | GG | CC | GG | GG |
| 58 | AFR | HG03195 | GG | AA | AA | GG | CC | GG | GC |
| 59 | AFR | HG03196 | GG | AA | AG | GG | CC | GG | GA |
| 60 | AFR | HG03198 | GG | GA | AG | GG | CC | GG | GG |
| 61 | AFR | HG03199 | GG | GA | AG | GG | CC | GG | CA |
| 62 | AFR | HG03202 | GG | GG | AA | GG | CC | GG | GC |
| 63 | AFR | HG03265 | GG | GG | AA | GG | CC | GG | CG |
| 64 | AFR | HG03267 | GG | GA | AA | GG | CC | GG | GG |
| 65 | AFR | HG03268 | GG | GG | AA | GG | CC | GG | GA |
| 66 | AFR | HG03270 | GG | GG | AA | GG | CC | GG | AG |
| 67 | AFR | HG03271 | AG | AA | AA | GG | CC | GG | GG |
| 68 | AFR | HG03279 | GG | AG | AA | GG | CC | AG | GC |
| 69 | AFR | HG03280 | GG | GG | AA | GG | CC | GG | CG |
| 70 | AFR | HG03291 | GG | GG | AA | GG | CC | GG | AG |
| 71 | AFR | HG03294 | GG | AA | AA | GG | CC | GG | AC |
| 72 | AFR | HG03295 | GG | AG | AA | GG | CC | AG | GC |
| 73 | AFR | HG03297 | AG | GG | AG | GG | CC | GG | GG |
| 74 | AFR | HG03298 | GG | GG | AA | GG | CC | GG | GG |
| 75 | AFR | HG03300 | GG | GA | AA | GG | CC | AG | GG |
| 76 | AFR | HG03301 | GG | GG | AA | GG | CC | GG | CC |
| 77 | AFR | HG03303 | GG | GG | AA | GG | CC | GG | GA |
| 78 | AFR | HG03304 | GG | GA | AA | GG | CC | GG | GC |
| 79 | AFR | HG03311 | GG | GA | AG | GG | CC | GG | GC |
| 80 | AFR | HG03313 | GG | GG | AA | GG | CC | GG | CG |
| 81 | AFR | HG03342 | GG | GG | AA | GG | CC | GG | GG |
| 82 | AFR | HG03343 | GG | GA | AG | GG | CC | GG | AG |
| 83 | AFR | HG03351 | GG | GA | AA | GG | CC | AG | AA |
| 84 | AFR | HG03352 | GG | GG | AA | GG | CC | GG | GC |
| 85 | AFR | HG03354 | GG | GA | AA | GG | CC | GG | AG |
| 86 | AFR | HG03363 | GG | GA | AA | GG | CC | GG | GA |
| 87 | AFR | HG03366 | GG | GG | AA | GG | CC | GG | AC |
| 88 | AFR | HG03367 | GG | GG | AG | GG | CC | GG | GA |
| 89 | AFR | HG03369 | GG | GA | AA | GG | CC | AG | GG |
| 90 | AFR | HG03370 | GG | GG | AA | GG | CC | GG | GA |
| 91 | AFR | HG03372 | GG | AA | AA | GG | CC | GG | GA |
| 92 | AFR | HG03499 | GG | GG | AA | GG | CC | GG | CG |
| 93 | AFR | HG03511 | GG | AA | AA | GG | CC | GG | GC |
| 94 | AFR | HG03514 | GG | AA | AA | GG | CC | GG | CG |
| 95 | AFR | HG03515 | GG | GA | AA | GG | CC | GG | CG |
| 96 | AFR | HG03517 | GG | AA | AA | GG | CC | GG | CG |
| 97 | AFR | HG03518 | AG | GA | AA | GG | CC | GG | CG |
| 98 | AFR | HG03520 | GG | AA | AA | GG | CC | AG | GC |
| 99 | AFR | HG03521 | GG | GA | AA | GG | CC | GG | GC |
| 100 | AMR | HGDP00702 | GG | GG | AA | AA | TT | AG | AG |
| 101 | AMR | HGDP00703 | GG | AG | AA | AA | TT | AA | GG |
| 102 | AMR | HGDP00704 | GG | GG | AA | AG | TT | AA | AG |
| 103 | AMR | HGDP00706 | GG | GG | AA | AA | TT | AA | AG |
| 104 | AMR | HGDP00708 | GG | GG | AA | AA | TT | AA | AG |
| 105 | AMR | HGDP00710 | GG | GG | AA | AG | TT | AA | AG |
| 106 | AMR | HGDP00832 | GG | GG | GG | AA | TT | AA | AG |
| 107 | AMR | HGDP00837 | GG | GG | GA | AA | TT | AG | AG |
| 108 | AMR | HGDP00838 | GG | GG | AA | AA | TT | AA | AG |
| 109 | AMR | HGDP00843 | GG | GG | AA | AA | TT | AA | AG |
| 110 | AMR | HGDP00845 | GG | GG | AA | AA | TT | AA | AG |
| 111 | AMR | HGDP00846 | GG | GG | AA | AA | TT | AA | AG |
| 112 | AMR | HGDP00849 | GG | GG | GA | AA | TT | AA | GG |
| 113 | AMR | HGDP00852 | GG | GG | GA | AG | TT | AA | AA |
| 114 | AMR | HGDP00854 | GG | GG | GA | AA | TT | AA | AG |
| 115 | AMR | HGDP00855 | GG | GG | AA | AG | TT | AG | AA |
| 116 | AMR | HGDP00856 | GG | GG | AA | GG | TT | AA | AG |
| 117 | AMR | HGDP00857 | GG | GG | AA | AG | TT | AG | AG |
| 118 | AMR | HGDP00858 | GG | GG | GA | AA | TT | AA | AG |
| 119 | AMR | HGDP00859 | GG | GG | AA | AG | TT | AA | AA |
| 120 | AMR | HGDP00860 | GG | AG | GA | AA | TT | AG | AA |
| 121 | AMR | HGDP00861 | GG | GG | GA | AG | TT | AA | GG |
| 122 | AMR | HGDP00862 | GG | GG | AA | AA | TT | AA | AA |
| 123 | AMR | HGDP00863 | GG | GG | AA | AA | TT | AA | AA |
| 124 | AMR | HGDP00864 | GG | GG | GA | AG | TT | AA | AA |
| 125 | AMR | HGDP00865 | GG | GG | AA | GG | TT | AA | AA |
| 126 | AMR | HGDP00868 | GG | GG | AA | GG | TT | AA | AA |
| 127 | AMR | HGDP00869 | GG | GG | AA | GG | TT | AA | AA |
| 128 | AMR | HGDP00870 | GG | GG | AA | AG | TT | AA | AG |
| 129 | AMR | HGDP00871 | GG | AA | AA | AG | TT | AG | GG |
| 130 | AMR | HGDP00872 | GG | GG | AA | GG | CT | GG | GG |
| 131 | AMR | HGDP00873 | GG | GG | AA | AA | TT | AA | AG |
| 132 | AMR | HGDP00875 | GG | GG | AA | AG | TT | AG | AA |
| 133 | AMR | HGDP00876 | GG | GG | GA | GG | TT | GG | AG |
| 134 | AMR | HGDP00877 | GG | GG | AA | AG | TT | AA | AG |
| 135 | AMR | HGDP00970 | GG | GG | AA | AA | TT | AA | AG |
| 136 | AMR | HGDP00995 | GG | GG | AA | AA | TT | AA | GG |
| 137 | AMR | HGDP00998 | GG | GG | AA | AA | TT | AA | AA |
| 138 | AMR | HGDP00999 | GG | GG | AA | AA | TT | AA | AA |
| 139 | AMR | HGDP01001 | GG | GG | AA | AA | TT | AA | GG |
| 140 | AMR | HGDP01003 | GG | GG | AA | AA | TT | AA | AA |
| 141 | AMR | HGDP01006 | GG | GG | AA | AA | TT | AA | GG |
| 142 | AMR | HGDP01009 | GG | GG | AA | AA | TT | AA | AG |
| 143 | AMR | HGDP01010 | GG | GG | AA | AA | TT | AA | AA |
| 144 | AMR | HGDP01012 | GG | GG | AA | AA | TT | AA | AG |
| 145 | AMR | HGDP01013 | GG | GG | AA | AA | TT | AA | AG |
| 146 | AMR | HGDP01014 | GG | GG | AA | AG | TT | AA | GG |
| 147 | AMR | HGDP01015 | GG | GG | AA | AA | TT | AA | AG |
| 148 | AMR | HGDP01018 | GG | GG | AA | AA | TT | AA | GG |
| 149 | AMR | HGDP01019 | GG | GG | AA | AA | TT | AA | GG |
| 150 | AMR | HGDP01037 | GG | AG | GA | AG | TT | AA | AG |
| 151 | AMR | HGDP01041 | GG | GG | AA | AG | TT | AA | AG |
| 152 | AMR | HGDP01043 | GG | GG | AA | AA | TT | AA | AG |
| 153 | AMR | HGDP01044 | GG | GG | AA | AA | TT | AA | AA |
| 154 | AMR | HGDP01047 | GG | GG | AA | AG | TT | AA | GG |
| 155 | AMR | HGDP01050 | GG | GG | AA | AA | TT | AA | AG |
| 156 | AMR | HGDP01051 | GG | GG | AA | AA | TT | AA | AG |
| 157 | AMR | HGDP01053 | GG | GG | AA | AA | TT | AG | GG |
| 158 | AMR | HGDP01055 | GG | AA | AA | GG | TT | AG | NN |
| 159 | AMR | HGDP01056 | GG | GG | AA | AA | TT | AA | AG |
| 160 | AMR | HGDP01057 | GG | GG | AA | AG | TT | AA | AG |
| 161 | AMR | HGDP01058 | GG | GG | GA | AG | TT | AG | GG |
| 162 | AMR | HGDP01059 | GG | GG | AA | AA | TT | AG | AA |
| 163 | AMR | HGDP01060 | GG | GG | AA | AG | TT | AA | AG |
| 164 | EAS | NA18939 | GG | AG | AA | GG | TT | AG | GG |
| 165 | EAS | NA18940 | GG | AA | AA | GG | TT | AA | AG |
| 166 | EAS | NA18941 | GG | GG | AA | GG | TT | AA | GG |
| 167 | EAS | NA18942 | GG | GG | AA | GG | TT | AA | GG |
| 168 | EAS | NA18943 | GG | AA | AA | GG | TT | AG | GG |
| 169 | EAS | NA18944 | GG | AG | AA | GG | TT | AA | GG |
| 170 | EAS | NA18945 | GG | AG | AA | GG | TT | AA | AG |
| 171 | EAS | NA18946 | GG | AA | AG | GG | TT | AA | AG |
| 172 | EAS | NA18947 | GG | GG | AA | GG | TT | AA | GG |
| 173 | EAS | NA18948 | GG | AG | AA | GG | TT | AA | AA |
| 174 | EAS | NA18949 | GG | GG | AA | GG | TT | GG | GG |
| 175 | EAS | NA18950 | GG | GG | AA | GG | TT | AA | GG |
| 176 | EAS | NA18951 | GG | AG | AA | GG | TT | AA | AG |
| 177 | EAS | NA18952 | GG | AG | AA | GG | TT | AA | AG |
| 178 | EAS | NA18953 | GG | AA | AA | GG | TT | AA | AA |
| 179 | EAS | NA18954 | GG | AG | AA | GG | TT | AG | AG |
| 180 | EAS | NA18956 | GG | AG | AA | GG | TT | AA | GG |
| 181 | EAS | NA18957 | GG | AA | AA | GG | TT | AA | AG |
| 182 | EAS | NA18959 | GG | AA | AA | GG | TT | AG | GG |
| 183 | EAS | NA18960 | GG | AG | AA | GG | TT | AG | AG |
| 184 | EAS | NA18961 | GG | AG | AA | GG | TT | AA | AG |
| 185 | EAS | NA18962 | GG | GG | AA | GG | TT | AA | AG |
| 186 | EAS | NA18963 | GG | GG | AA | GG | TT | AG | AG |
| 187 | EAS | NA18964 | GG | AG | AA | GG | TT | AA | AG |
| 188 | EAS | NA18965 | GG | AG | AG | GG | TT | AA | AA |
| 189 | EAS | NA18966 | GG | GG | AA | GG | TT | AA | AG |
| 190 | EAS | NA18968 | GG | AG | AA | GG | TT | AA | AG |
| 191 | EAS | NA18971 | GG | AG | AA | GG | TT | AA | GG |
| 192 | EAS | NA18973 | GG | AA | AA | GG | TT | AG | AG |
| 193 | EAS | NA18974 | GG | AG | AA | GG | TT | AG | GG |
| 194 | EAS | NA18975 | GG | GG | AA | GG | TT | AA | AG |
| 195 | EAS | NA18976 | GG | AG | AA | GG | TT | AA | GG |
| 196 | EAS | NA18977 | GG | AG | AA | GG | TT | AA | AA |
| 197 | EAS | NA18978 | GG | GG | AA | GG | TT | AA | GG |
| 198 | EAS | NA18980 | GG | AA | AA | GG | TT | AA | GG |
| 199 | EAS | NA18981 | GG | GG | AG | GG | TT | AG | AG |
| 200 | EAS | NA18982 | GG | GG | AA | GG | TT | AG | GG |
| 201 | EAS | NA18983 | GG | GG | AA | GG | TT | AG | AG |
| 202 | EAS | NA18984 | GG | AG | AA | GG | TT | AA | AG |
| 203 | EAS | NA18985 | GG | GG | AA | GG | TT | AG | GG |
| 204 | EAS | NA18986 | GG | AG | AA | GG | TT | AA | AG |
| 205 | EAS | NA18987 | GG | AA | AA | GG | TT | AG | AG |
| 206 | EAS | NA18988 | GG | GG | AA | GG | TT | GG | GG |
| 207 | EAS | NA18989 | GG | AG | AA | GG | TT | AA | AG |
| 208 | EAS | NA18990 | GG | AA | AA | GG | TT | AA | AG |
| 209 | EAS | NA18992 | GG | GG | AG | GG | TT | AA | GG |
| 210 | EAS | NA18994 | GG | AG | AA | GG | TT | AA | AG |
| 211 | EAS | NA18995 | GG | GG | AA | GG | TT | AA | AG |
| 212 | EAS | NA18998 | GG | AG | AA | GG | TT | AA | GG |
| 213 | EAS | NA18999 | GG | GG | AA | GG | TT | AA | AA |
| 214 | EAS | NA19000 | GG | AA | AA | GG | TT | AG | AG |
| 215 | EAS | NA19002 | GG | AG | AA | GG | TT | AA | AG |
| 216 | EAS | NA19003 | GG | GG | AA | GG | TT | AA | AG |
| 217 | EAS | NA19004 | GG | AA | AA | GG | TT | AA | GG |
| 218 | EAS | NA19005 | GG | GG | AA | GG | TT | AA | AA |
| 219 | EAS | NA19007 | GG | GG | AA | GG | TT | AG | GG |
| 220 | EAS | NA19009 | GG | GG | AA | GG | TT | AG | AG |
| 221 | EAS | NA19010 | GG | AG | AA | GG | TT | AA | GG |
| 222 | EAS | NA19012 | GG | AA | AA | GG | TT | AG | GG |
| 223 | EAS | NA19054 | GG | AG | AA | GG | TT | AA | GG |
| 224 | EAS | NA19055 | GG | AG | AA | GG | TT | AA | AA |
| 225 | EAS | NA19056 | GG | AG | AA | GG | TT | AA | AG |
| 226 | EAS | NA19057 | GG | GG | AG | GG | TT | AA | AG |
| 227 | EAS | NA19058 | GG | AA | AA | GG | TT | AA | AA |
| 228 | EAS | NA19059 | GG | GG | AA | GG | TT | AA | GG |
| 229 | EAS | NA19060 | GG | GG | AA | GG | TT | AA | GG |
| 230 | EAS | NA19062 | GG | AA | AA | GG | TT | AA | AG |
| 231 | EAS | NA19063 | GG | AG | AA | GG | TT | AA | AG |
| 232 | EAS | NA19064 | GG | AG | AA | GG | TT | AG | GG |
| 233 | EAS | NA19065 | GG | AG | AA | GG | TT | AA | AG |
| 234 | EAS | NA19066 | GG | AG | AG | GG | TT | AG | GG |
| 235 | EAS | NA19067 | GG | GG | AA | GG | TT | AA | GG |
| 236 | EAS | NA19068 | GG | GG | AA | GG | TT | AA | GG |
| 237 | EAS | NA19070 | GG | AG | GG | GG | TT | AG | AG |
| 238 | EAS | NA19072 | GG | AG | AA | GG | TT | AA | GG |
| 239 | EAS | NA19074 | GG | GG | AA | GG | TT | AA | AA |
| 240 | EAS | NA19075 | GG | GG | AA | GG | TT | AA | AG |
| 241 | EAS | NA19076 | GG | GG | AA | GG | TT | AG | AA |
| 242 | EAS | NA19077 | GG | AA | AA | GG | TT | AA | GG |
| 243 | EAS | NA19078 | GG | GG | AG | GG | TT | AA | AA |
| 244 | EAS | NA19079 | GG | AG | AA | GG | TT | AA | AG |
| 245 | EAS | NA19080 | GG | AA | AA | GG | TT | AA | AG |
| 246 | EAS | NA19081 | GG | GG | AA | GG | TT | AG | GG |
| 247 | EAS | NA19082 | GG | GG | AG | GG | TT | AA | AA |
| 248 | EAS | NA19083 | GG | AG | AA | GG | TT | AA | GG |
| 249 | EAS | NA19084 | GG | AG | AG | GG | TT | AA | AG |
| 250 | EAS | NA19085 | GG | AG | AA | GG | TT | AA | AG |
| 251 | EAS | NA19087 | GG | AG | AA | GG | TT | AA | GG |
| 252 | EAS | NA19088 | GG | AA | AG | GG | TT | AA | AG |
| 253 | EUR | HG00096 | AG | AA | AA | GG | TT | GG | GG |
| 254 | EUR | HG00097 | AA | AA | GA | GG | TT | GG | AA |
| 255 | EUR | HG00099 | AA | GG | AA | GA | TT | GG | GA |
| 256 | EUR | HG00100 | AA | AA | AG | GG | TT | GG | GG |
| 257 | EUR | HG00101 | AA | AG | AA | GG | TT | GG | GG |
| 258 | EUR | HG00102 | AA | AA | AG | GG | TT | GG | GA |
| 259 | EUR | HG00103 | GA | AA | AA | GG | TT | GG | AG |
| 260 | EUR | HG00104 | AA | GA | GA | GG | TT | GG | GG |
| 261 | EUR | HG00106 | AA | AA | AA | GG | TT | AG | GA |
| 262 | EUR | HG00108 | AA | AA | AA | GG | TT | GG | AG |
| 263 | EUR | HG00109 | AA | AA | GA | GG | TT | GG | AA |
| 264 | EUR | HG00110 | AA | AA | GA | GG | TT | AG | AG |
| 265 | EUR | HG00111 | GA | AA | AA | GG | TT | GG | AG |
| 266 | EUR | HG00112 | AA | AA | GG | GG | TT | AG | GG |
| 267 | EUR | HG00113 | AA | AA | GA | GG | TT | GG | GG |
| 268 | EUR | HG00114 | AA | GA | GA | GG | TT | AA | GA |
| 269 | EUR | HG00116 | AA | AG | AA | GG | TT | GG | GG |
| 270 | EUR | HG00117 | AA | AG | AA | GG | TT | GG | AG |
| 271 | EUR | HG00118 | GA | AA | AA | GG | TT | AG | AA |
| 272 | EUR | HG00119 | AG | AA | AA | AG | TT | GG | AG |
| 273 | EUR | HG00120 | AG | GG | AA | GG | TT | GG | GG |
| 274 | EUR | HG00121 | AA | AA | AA | GG | TT | GG | AG |
| 275 | EUR | HG00122 | AA | AA | AG | GG | TT | GA | AG |
| 276 | EUR | HG00123 | AG | AG | AG | GA | TT | GA | AG |
| 277 | EUR | HG00124 | AA | AA | AA | GG | TT | GG | GG |
| 278 | EUR | HG00125 | AA | AG | GA | GG | TT | GG | AG |
| 279 | EUR | HG00126 | AG | AA | AA | GG | TT | GG | AA |
| 280 | EUR | HG00127 | AA | AG | AA | GG | TT | GA | GG |
| 281 | EUR | HG00128 | AG | AA | AA | GG | TT | GG | GG |
| 282 | EUR | HG00129 | AA | AA | AA | GG | TT | GG | AG |
| 283 | EUR | HG00130 | AG | AA | GA | GG | TT | GG | AA |
| 284 | EUR | HG00131 | AA | AG | GA | GA | TT | GG | GG |
| 285 | EUR | HG00133 | AA | AA | AA | GG | TT | GG | GA |
| 286 | EUR | HG00134 | AG | AG | GA | GG | TT | GG | AA |
| 287 | EUR | HG00135 | AA | AA | GG | GG | TT | GG | AA |
| 288 | EUR | HG00136 | GA | AA | AG | GG | TT | GA | GA |
| 289 | EUR | HG00137 | AA | AA | AA | GG | TT | GG | GA |
| 290 | EUR | HG00138 | AA | GA | AA | GG | TT | GG | GA |
| 291 | EUR | HG00139 | AG | AG | AA | GG | TT | GG | AG |
| 292 | EUR | HG00140 | GA | GA | AG | AA | TT | GG | AG |
| 293 | EUR | HG00141 | AG | AA | AG | GG | TT | GA | AA |
| 294 | EUR | HG00142 | AG | GA | AA | GG | TT | GG | AA |
| 295 | EUR | HG00143 | AA | AA | GA | GG | TT | AG | GG |
| 296 | EUR | HG00146 | AG | AA | AA | GG | TT | GG | GA |
| 297 | EUR | HG00148 | AA | AA | AA | GA | TT | GG | AA |
| 298 | EUR | HG00149 | AA | AG | AA | GG | TT | GG | AA |
| 299 | EUR | HG00150 | AA | AA | AA | GG | TT | GG | GG |
| 300 | EUR | HG00151 | GA | AG | AA | GG | TT | GA | AA |
| 301 | EUR | HG00152 | AG | GA | AA | AG | TT | GG | AA |
| 302 | EUR | HG00154 | GA | GG | GA | GG | TT | GG | AG |
| 303 | EUR | HG00156 | AA | AA | AG | GG | TT | GG | AG |
| 304 | EUR | HG00158 | AA | AA | AA | GG | TT | GG | AA |
| 305 | EUR | HG00159 | AA | AA | AA | AG | TT | GG | AA |
| 306 | EUR | HG00160 | AA | AA | GA | GG | TT | AG | AA |
| 307 | EUR | HG00231 | AA | AA | AG | AG | TT | GG | GA |
| 308 | EUR | HG00232 | AA | AA | GA | GG | TT | GG | AG |
| 309 | EUR | HG00233 | AA | GA | AA | GG | TT | AG | GA |
| 310 | EUR | HG00234 | AA | GA | AG | GG | TT | GG | GA |
| 311 | EUR | HG00235 | AG | AA | AG | GA | TT | GG | AA |
| 312 | EUR | HG00236 | AA | AG | GA | GG | TT | GG | GA |
| 313 | EUR | HG00237 | GA | AA | AA | GG | TT | AG | GG |
| 314 | EUR | HG00238 | GG | AA | AA | GG | TT | GG | AA |
| 315 | EUR | HG00239 | AA | AA | GG | GG | TT | GG | GA |
| 316 | EUR | HG00240 | GA | AA | AA | GG | TT | GG | AA |
| 317 | EUR | HG00242 | AA | GA | AA | GG | TT | GG | GG |
| 318 | EUR | HG00243 | AA | GG | AG | GG | TT | GG | GG |
| 319 | EUR | HG00244 | AA | AA | GA | GG | TT | GG | AG |
| 320 | EUR | HG00245 | AA | AA | AA | GG | TT | GG | AG |
| 321 | EUR | HG00246 | AA | AA | AA | GG | TT | GG | AA |
| 322 | EUR | HG00247 | AA | GA | AA | GG | TT | GG | AA |
| 323 | EUR | HG00249 | GA | AA | AA | GA | TT | GG | GA |
| 324 | EUR | HG00250 | AG | AA | AA | GG | TT | GG | AA |
| 325 | EUR | HG00251 | GA | AG | AA | GG | TT | GG | GA |
| 326 | EUR | HG00252 | AA | AA | AA | GG | TT | GG | AA |
| 327 | EUR | HG00253 | AA | AG | GA | GG | TT | AG | GG |
| 328 | EUR | HG00254 | AA | AA | AA | GG | TT | GG | GG |
| 329 | EUR | HG00255 | AA | GA | AA | GG | TT | GG | GG |
| 330 | EUR | HG00256 | AA | AG | AA | GG | TT | AG | GG |
| 331 | EUR | HG00257 | GG | AA | AG | GG | TT | AG | GG |
| 332 | EUR | HG00258 | AA | AA | GA | AG | TT | GG | AA |
| 333 | EUR | HG00259 | AG | AA | AG | GA | TT | AG | GG |
| 334 | EUR | HG00260 | AG | AA | AA | GG | TT | GG | AA |
| 335 | EUR | HG00261 | GG | GG | AA | GG | TT | GG | GA |
| 336 | EUR | HG00262 | AA | GA | AA | GA | TT | GG | AA |
| 337 | EUR | HG00263 | AA | AA | AA | GG | TT | GG | AG |
| 338 | EUR | HG00264 | AA | AA | AA | GG | TT | GG | AG |
| 339 | EUR | HG00265 | AG | AA | AA | GG | TT | GG | AA |
| 340 | EUR | HG01334 | GA | AA | AG | AA | TT | GG | GG |
| 341 | OCE | HGDP00491 | GG | AG | GG | GG | TT | AA | GG |
| 342 | OCE | HGDP00540 | GG | AA | GG | GG | TT | AA | CC |
| 343 | OCE | HGDP00541 | GG | AA | GG | GG | TT | AA | GG |
| 344 | OCE | HGDP00542 | GG | AG | GG | GG | TT | AA | GG |
| 345 | OCE | HGDP00543 | GG | AA | GG | GG | TT | AA | GG |
| 346 | OCE | HGDP00544 | GG | GG | GG | GG | TT | AA | GG |
| 347 | OCE | HGDP00545 | GG | AA | GG | GG | TT | AA | GG |
| 348 | OCE | HGDP00546 | GG | AA | GG | GG | TT | AA | CG |
| 349 | OCE | HGDP00547 | GG | AG | GG | GG | TT | AA | GG |
| 350 | OCE | HGDP00548 | GG | AG | GG | GG | TT | AA | GG |
| 351 | OCE | HGDP00549 | GG | AG | GG | GG | TT | AA | NN |
| 352 | OCE | HGDP00550 | GG | AA | GG | GG | TT | AA | GG |
| 353 | OCE | HGDP00551 | GG | AG | GG | GG | TT | AA | CG |
| 354 | OCE | HGDP00552 | GG | AA | GG | GG | TT | AA | GG |
| 355 | OCE | HGDP00553 | GG | GG | GG | GG | TT | AA | GG |
| 356 | OCE | HGDP00554 | GG | GG | GG | GG | TT | AA | GG |
| 357 | OCE | HGDP00555 | GG | AG | GG | GG | TT | AA | GG |
| 358 | OCE | HGDP00556 | GG | AG | GG | GG | TT | AA | GG |
| 359 | OCE | HGDP00655 | GG | AA | GG | GG | TT | AA | GG |
| 360 | OCE | HGDP00656 | GG | AA | GG | GG | TT | AA | CG |
| 361 | OCE | HGDP00661 | GG | AA | GA | GG | TT | AA | CC |
| 362 | OCE | HGDP00662 | GG | AG | GG | GG | TT | AA | CG |
| 363 | OCE | HGDP00663 | GG | GG | GG | GG | TT | AA | AG |
| 364 | OCE | HGDP00664 | GG | AA | GG | GG | TT | AA | CG |
| 365 | OCE | HGDP00787 | GG | AG | GG | GG | TT | AA | CC |
| 366 | OCE | HGDP00788 | GG | AA | GG | GG | TT | AA | GG |
| 367 | OCE | HGDP00979 | GG | AG | GG | GG | TT | AA | CG |
| 368 | OCE | HGDP01027 | GG | AG | GG | GG | TT | AA | CC |
| 369 | Baloach | B2 | GA | AA | AA | GG | TT | GA | GA |
| 370 | Baloach | B4 | GA | AA | GA | GG | TC | GA | GA |
| 371 | Baloach | B5 | GA | AA | AA | GG | TT | GA | GA |
| 372 | Baloach | B6 | GA | AA | AG | GG | TT | GA | GA |
| 373 | Gilgit | G9 | GG | AA | AA | GG | TT | GA | GA |
| 374 | Gilgit | Gil7 | GA | AG | AG | GG | TT | GA | GA |
| 375 | Gilgit | Gil8 | GG | AA | AA | GG | TT | GG | GA |
| 376 | Gilgit | Gil9 | GG | AA | AA | GG | TT | GA | GA |
| 377 | Gilgit | Gil11 | GA | AG | AG | GG | TT | GG | GA |
| 378 | Kashmiri | K1 | GA | AA | AG | GG | TT | GA | GC |
| 379 | Kashmiri | K3 | GA | AA | NN | GG | TT | GG | GA |
| 380 | Kashmiri | K4 | GA | AA | AG | GA | TT | GG | GA |
| 381 | Kashmiri | K7 | GA | AG | AA | GG | TT | GA | GA |
| 382 | Kashmiri | K8 | GA | AG | AG | GG | TT | GA | GA |
| 383 | Pathan | P9 | GA | AA | AG | GG | TT | GA | GA |
| 384 | Pathan | P11 | GA | AG | AG | GG | TT | GG | GA |
| 385 | Pathan | P12 | GG | AG | AA | GG | TT | GG | GA |
| 386 | Pathan | P14 | GA | AA | AA | GG | TT | GG | GG |
| 387 | Pothwari | PT32 | GA | AG | AG | GG | TT | GA | GA |
| 388 | Pothwari | PT34 | GA | AA | AG | GG | TT | GA | GA |
| 389 | Pothwari | PT39 | GG | AG | NN | GG | TT | GG | GA |
| 390 | Pothwari | PT45 | GG | AA | AA | GG | TT | GA | GA |
| 391 | Pothwari | PT50 | GA | AG | AA | GG | TT | GG | GG |
| 392 | Siraiki | R1 | GG | AA | AG | GG | TT | GA | GA |
| 393 | Siraiki | R2 | GA | AA | AG | GA | TT | GA | GA |
| 394 | Siraiki | R3 | GA | AA | AG | GG | TT | GA | GA |
| 395 | Siraiki | R5 | GG | AA | AA | GG | TT | GA | GA |
| 396 | Siraiki | R7 | GG | AA | AG | GA | TT | GA | GA |

| **rs3827760** | **rs16830500** | **rs10183022** | **rs7623065** | **rs9809818** | **rs6437783** | **rs12498138** | **rs820371** | **rs4683510** | **rs10012227** |
| --- | --- | --- | --- | --- | --- | --- | --- | --- | --- |
| AA | TT | AA | AG | AA | TT | GG | CT | CC | GG |
| AA | TT | AA | AG | AA | CT | GG | TT | CT | GG |
| AA | TT | AA | GG | AA | TT | GG | TT | TC | GG |
| AA | TT | AA | AG | AA | TT | GG | TT | CC | GG |
| AA | TT | AA | GG | AA | CT | GG | TT | CC | GG |
| AA | TT | AA | AG | AA | CT | GG | TT | CT | GG |
| AA | TT | AA | AG | AA | CT | GG | CT | CC | GG |
| AA | TT | AA | GG | AA | TT | GG | CT | CC | GG |
| AA | TT | AA | GG | AA | TT | GG | TT | CT | GG |
| AA | TT | AA | AG | AA | TT | GG | TT | TT | GG |
| AA | TT | AA | GG | AC | TT | GG | TT | CC | GG |
| AA | TT | AA | GG | AA | TT | GG | TT | CC | GG |
| AA | TT | AA | AG | AA | TT | GG | TT | CC | GG |
| AA | TT | AA | GG | AA | TT | GG | TT | CT | GG |
| AA | CT | AA | GG | AA | CT | GG | CT | CC | GG |
| AA | TT | AA | GG | AA | CT | GG | TT | CC | GG |
| AA | TT | AA | AG | AA | TT | GG | TT | CC | AG |
| AA | CT | AA | GG | AA | TT | GG | TT | CT | GG |
| AA | TT | AA | GG | AA | TT | GG | TT | CC | AG |
| AA | TT | AA | GG | AA | CT | GG | TT | TT | GG |
| AA | CT | AA | GG | AA | TT | GG | TT | CC | GG |
| AA | TT | AA | GG | AA | TT | GG | TT | CC | GG |
| AA | TT | AA | GG | AA | TT | GG | TT | TC | GG |
| AA | TT | AA | GG | AA | CT | GG | TT | TC | GG |
| AA | CT | AA | AG | AA | CT | GG | TT | CC | GG |
| AA | TT | AA | AA | AA | TT | GG | TT | CT | AG |
| AA | TT | AA | AG | AA | TT | GG | TT | CC | GG |
| AA | TT | AA | GG | AA | CT | GG | TT | CT | GG |
| AA | TT | AA | GG | AA | CT | GG | TT | TC | AG |
| AA | TT | AA | GG | AA | TT | GG | TT | CT | GG |
| AA | TT | AA | GG | AA | TT | GG | TT | TC | GG |
| AA | CT | AA | GG | AA | TT | GG | TT | TT | AA |
| AA | TT | AA | GG | AA | TT | GG | TT | CT | AG |
| AA | TT | AA | GG | AA | TT | GG | TT | TC | GG |
| AA | TT | AA | GG | AA | CT | GG | TT | CC | AG |
| AA | TT | AA | GG | AA | TT | GG | TT | CC | GG |
| AA | TT | AA | GG | AA | TT | GG | TT | TC | GG |
| AA | CT | AA | GG | AA | TT | GG | CT | TC | GG |
| AA | CT | AA | GG | AA | CT | GG | TT | CC | GG |
| AA | TT | AA | GG | AA | TT | GG | TT | CC | AG |
| AA | TT | AA | GG | AA | TT | GG | TT | CC | GG |
| AA | TT | AA | GG | AA | TT | GG | TT | CT | GG |
| AA | TT | AA | GG | AA | CT | GG | TT | CC | GG |
| AA | TT | AA | AG | AA | CT | GG | TT | CT | GG |
| AA | TT | AA | GG | AA | TT | GG | TT | CT | AG |
| AA | TT | AA | GG | AA | TT | GG | TT | CC | GG |
| AA | TT | AA | AG | AA | TT | GG | TT | CT | GG |
| AA | CT | AA | GG | AA | TT | GG | TT | CC | GG |
| AA | TT | AA | GG | AA | TT | GG | TT | CC | GG |
| AA | TT | AA | AG | AA | CT | GG | TT | CC | AG |
| AA | TT | AA | AG | AA | CT | GG | TT | CC | GG |
| AA | TT | AA | AG | AA | TT | GG | TT | CC | GG |
| AA | TT | AA | GG | AA | TT | GG | TT | CC | GG |
| AA | TT | AA | GG | AA | CT | GG | TT | CC | GG |
| AA | TT | AA | GG | AA | TT | GG | TT | CT | GG |
| AA | TT | AA | GG | AA | TT | GG | TT | TC | GG |
| AA | TT | AA | GG | AA | TT | GG | TT | CC | GG |
| AA | TT | AA | AG | AA | CT | GG | TT | CC | GG |
| AA | TT | AA | GG | AC | CT | GG | TT | CT | GG |
| AA | TT | AA | GG | AA | CT | GG | TT | TC | GG |
| AA | TT | AA | GG | AA | TT | GG | CT | TC | GG |
| AA | TT | AA | GG | AA | CT | GG | TT | CT | AG |
| AA | TT | AA | GG | AA | TT | GG | TT | CC | GG |
| AA | TT | AA | AG | AA | CT | GG | TT | CT | AG |
| AA | TT | AA | GG | AA | TT | GG | CT | CT | GG |
| AA | TT | AA | GG | AA | TT | GG | TT | TT | GG |
| AA | TT | AA | GG | AA | TT | GG | CT | CC | GG |
| AA | TT | AA | AG | AA | TT | GG | TT | TC | GG |
| AA | TT | AA | AG | AA | TT | GG | TT | TC | AG |
| AA | TT | AA | GG | AA | TT | GG | TT | CC | GG |
| AA | TT | AA | GG | AA | TT | GG | CT | CC | GG |
| AA | CT | AA | GG | AA | CT | GG | TT | CT | GG |
| AA | TT | AA | GG | AA | TT | GG | TT | CC | GG |
| AA | TT | AA | AG | AA | CT | GG | TT | CC | GG |
| AA | TT | AA | AG | AA | CT | GG | CT | CC | GG |
| AA | TT | AA | GG | AA | TT | GG | TT | CC | GG |
| AA | TT | AA | AA | AA | TT | GG | TT | CC | GG |
| AA | TT | AA | AG | AA | CT | GG | TT | CT | GG |
| AA | TT | AA | AG | AA | TT | GG | TT | CC | GG |
| AA | TT | AA | GG | AA | CC | GG | TT | CC | GG |
| AA | TT | AA | AG | AA | TT | GG | TT | CC | AG |
| AA | TT | AA | AG | AA | CC | GG | TT | CC | GG |
| AA | TT | AA | GG | AA | CC | GG | CT | CT | GG |
| AA | CT | AA | GG | AA | TT | GG | TT | TC | GG |
| AA | TT | AA | GG | AA | TT | GG | TT | CT | GG |
| AA | TT | AA | GG | AA | CC | GG | TT | CT | GG |
| AA | TT | AA | GG | AA | CC | GG | TT | TC | GG |
| AA | TT | AA | GG | AA | CT | GG | TT | CC | GG |
| AA | TT | AA | AG | AA | CT | GG | TT | TT | AG |
| AA | TT | AA | GG | AA | CT | GG | CT | CC | AG |
| AA | TT | AA | AG | AA | CT | GG | TT | CC | GG |
| AA | TT | AA | GG | AA | CT | GG | TT | CC | AG |
| AA | CT | AA | GG | AA | TT | GG | CT | CC | GG |
| AA | TT | AA | AG | AA | TT | GG | TT | CC | GG |
| AA | TT | AA | GG | AA | TT | GG | TT | CC | GG |
| AA | TT | AA | AA | AA | TT | GG | TT | CC | GG |
| AA | TT | AA | AG | AA | TT | GG | TT | CC | GG |
| AA | TT | AA | AG | AA | CT | GG | CT | CC | GG |
| AA | CT | AA | AG | AA | TT | GG | TT | CC | GG |
| GG | TT | GG | AA | CC | CC | AA | CT | TT | AA |
| AG | TT | GG | AG | CC | CC | AA | TT | TT | AA |
| GG | TT | GG | AA | CC | CC | AA | TT | TT | AA |
| GG | TT | GG | AA | CC | CT | AA | TT | CT | AA |
| AG | TT | AG | AA | CC | CC | AA | CT | TT | AA |
| AG | TT | GG | AA | CC | CC | AA | TT | TT | AA |
| GG | TT | GG | AA | CC | CC | AA | TT | CT | AA |
| AG | TT | GG | AA | CC | CC | AA | CT | CT | AA |
| AA | TT | GG | AA | CC | CT | AA | CT | CT | AA |
| AG | TT | GG | AA | CC | CC | AA | TT | CT | AA |
| GG | TT | GG | AA | CC | CC | AA | CC | CC | AA |
| GG | TT | GG | AA | CC | CC | AA | TT | CT | AA |
| GG | TT | GG | AA | CC | CC | AA | TT | TT | AA |
| GG | TT | GG | AA | CC | CC | AA | CT | CC | AA |
| GG | CT | GG | AA | CC | CC | AA | TT | CC | AA |
| GG | TT | AG | AA | CC | CC | AA | CT | TT | AA |
| GG | TT | GG | AA | CC | CC | AA | CT | TT | AA |
| GG | TT | GG | AA | CC | CT | GA | TT | CT | AA |
| GG | TT | AG | AA | CC | CC | AA | CT | CT | AA |
| GG | TT | AA | AA | CC | CC | AA | CT | CT | AG |
| GG | CT | GG | AA | CC | CC | GA | CT | CT | AA |
| GG | CT | GG | AA | CC | CT | AA | CT | TT | AA |
| AG | TT | AG | AA | AC | CC | GG | TT | TT | AA |
| GG | TT | GG | AA | AC | CC | AA | TT | CC | AG |
| AG | TT | AG | AG | AC | CC | AA | TT | CC | AA |
| GG | TT | AG | AA | CC | CC | AA | CT | TT | AA |
| GG | TT | AG | AA | AC | CC | AA | TT | CC | AA |
| GG | TT | GG | AA | AC | CT | GA | CT | TT | AA |
| AG | TT | GG | AA | CC | CC | AA | TT | CT | AA |
| AG | CT | GG | AA | CC | CC | AA | CT | CT | AG |
| AG | TT | AG | AA | CC | CC | AA | TT | TT | AA |
| GG | CT | GG | AA | AC | CC | AA | TT | TT | AA |
| GG | TT | GG | AA | CC | CT | AA | TT | TT | AA |
| GG | TT | GG | AG | AC | CT | AA | CT | CT | AA |
| GG | CC | GG | AA | AC | CT | AA | TT | CT | AA |
| GG | TT | GG | AA | CC | CT | AA | CT | CT | AA |
| GG | TT | AG | AA | CC | CC | AA | CT | TT | AA |
| AA | TT | GG | AA | CC | CC | AA | TT | TT | AA |
| GG | TT | AG | AA | CC | CC | AA | CT | CT | AA |
| GG | TT | AG | AA | CC | CC | AA | TT | TT | AA |
| GG | TT | AA | AA | CC | CC | AA | CT | TT | AA |
| GG | TT | GG | AA | CC | CC | AA | CT | CT | AA |
| GG | TT | GG | AA | CC | CC | AA | TT | CT | AA |
| GG | TT | AG | AA | CC | CC | AA | CT | TT | AA |
| GG | TT | AA | AA | CC | CC | AA | CC | TT | AA |
| GG | TT | AA | AA | CC | CC | AA | CT | TT | AA |
| GG | TT | AG | AA | AC | CC | AA | TT | CT | AA |
| GG | TT | GG | AA | AC | CC | AA | TT | TT | AA |
| GG | TT | AG | AA | CC | CC | AA | TT | CC | AA |
| GG | TT | GG | AA | AC | CC | AA | CT | TT | AA |
| GG | TT | GG | AA | AC | CC | AA | CT | CT | AA |
| GG | TT | GG | AA | CC | CC | AA | TT | CT | AA |
| GG | CT | GG | AA | AC | CT | GA | TT | TT | AA |
| GG | CT | GG | AA | AC | CT | GA | TT | TT | AA |
| GG | TT | GG | AA | CC | CT | AA | TT | CT | AA |
| GG | CC | GG | AA | AA | CC | AA | TT | CT | AA |
| GG | CT | GG | AA | CC | CC | GA | CC | CC | AG |
| GG | TT | GG | AA | CC | CT | GA | TT | CT | AA |
| GG | CT | GG | AA | AC | CC | GA | CT | CT | AG |
| GG | CT | AG | AA | AA | CC | AA | CT | CT | AA |
| GG | TT | GG | AA | AC | CC | GA | CT | TT | AA |
| GG | TT | AG | AA | AC | CC | GA | CT | CC | AA |
| GG | CT | GG | AA | AC | CT | AA | TT | TT | AA |
| GG | CT | GG | AG | AC | CC | AA | TT | CT | AG |
| AG | CC | AG | AG | CC | CC | GG | TT | TT | AA |
| AA | CT | AG | AG | CC | CC | GG | TT | CT | AG |
| AG | TT | AG | AA | CC | CC | AG | TT | CT | AG |
| AG | CT | AG | AA | CC | CC | GG | TT | CT | AG |
| GG | CT | GG | AA | CC | CC | GG | TT | TT | GG |
| GG | CT | GG | AA | CC | CC | AG | TT | TT | GG |
| AG | CT | AG | AA | CC | CC | GG | TT | TT | AG |
| AG | CT | GG | AA | CC | CC | GG | TT | TT | AG |
| AG | CC | AG | AA | CC | CC | GG | TT | TT | AG |
| GG | CT | AG | AA | AC | CC | GG | TT | TT | AA |
| GG | CT | AG | AA | CC | CC | GG | TT | TT | GG |
| GG | CC | GG | AA | CC | CC | GG | TT | TT | AA |
| GG | TT | AA | AG | CC | CC | GG | TT | TT | GG |
| GG | CC | AG | AA | CC | CC | GG | TT | TT | AA |
| GG | CC | AG | AG | AC | CC | GG | CT | TT | GG |
| AG | TT | GG | AA | CC | CC | GG | TT | TT | AA |
| GG | TT | AG | AA | CC | CC | GG | TT | CT | GG |
| GG | CC | AA | AA | CC | CC | AG | TT | CT | AA |
| AG | CC | AG | AA | CC | CC | AG | TT | TT | AG |
| GG | CT | GG | AA | CC | CC | AG | TT | TT | GG |
| GG | CC | AG | AA | CC | CC | GG | TT | TT | GG |
| GG | CT | AG | AA | CC | CC | GG | TT | TT | GG |
| AG | CT | GG | AA | CC | CC | GG | TT | CT | GG |
| GG | CT | AA | AA | CC | CC | GG | TT | CT | GG |
| GG | TT | GG | AG | CC | CC | AG | TT | TT | AG |
| AG | CT | AG | AA | CC | CC | GG | TT | TT | AG |
| AG | CC | AG | AG | CC | CC | AG | TT | CT | GG |
| AG | CT | AG | AA | CC | CC | GG | TT | TT | GG |
| GG | CT | AG | AA | CC | CC | GG | CT | TT | AG |
| AA | TT | AA | AA | CC | CC | AA | CT | CT | GG |
| GG | CC | AG | AA | CC | CC | GG | TT | TT | AG |
| GG | CC | AG | AA | CC | CC | GG | TT | TT | GG |
| AG | TT | AG | AA | AC | CC | GG | TT | TT | AG |
| GG | CC | AG | AA | CC | CC | GG | TT | TT | AG |
| GG | CT | AG | AG | CC | CC | GG | TT | CT | AG |
| GG | TT | AA | AA | CC | CC | GG | TT | TT | AA |
| GG | CT | GG | AA | CC | CC | AA | TT | CT | AG |
| GG | TT | AG | AA | CC | CC | GG | TT | TT | GG |
| AG | TT | AA | AA | CC | CC | GG | TT | TT | AG |
| GG | TT | GG | AA | CC | CC | GG | TT | TT | AG |
| GG | CT | AG | AA | CC | CC | AA | TT | TT | AG |
| GG | TT | AG | AA | CC | CC | GG | TT | CT | AG |
| AG | CC | AG | AA | CC | CC | GG | TT | TT | AG |
| GG | CT | AA | AA | CC | CC | GG | TT | TT | AG |
| GG | CT | AG | AG | AC | CC | GG | TT | TT | AG |
| GG | TT | GG | AG | CC | CC | GG | TT | CT | AA |
| AG | CC | AG | AA | CC | CC | GG | TT | TT | AG |
| GG | CT | AG | AA | AC | CC | GG | TT | TT | AG |
| AG | CC | AG | AG | CC | CC | GG | TT | TT | GG |
| AG | CT | GG | AA | CC | CC | AG | TT | TT | AA |
| GG | CC | AG | AA | CC | CC | GG | TT | TT | GG |
| GG | CT | AA | AA | CC | CC | GG | TT | TT | AA |
| GG | CC | AA | AA | CC | CC | GG | CT | TT | AG |
| AG | CT | GG | AA | CC | CC | AG | TT | TT | GG |
| GG | TT | AG | AA | CC | CC | GG | CT | TT | AG |
| GG | CC | GG | AA | CC | CC | GG | TT | TT | AA |
| GG | CT | AG | AA | CC | CC | GG | CT | TT | GG |
| GG | CT | GG | AA | CC | CC | GG | TT | TT | AG |
| AA | CC | AG | AG | CC | CC | GG | TT | TT | AG |
| GG | CC | GG | AA | AC | CC | GG | TT | TT | GG |
| GG | CT | AG | AA | CC | CC | GG | TT | CT | AG |
| AG | CT | AG | AA | CC | CC | GG | CT | CT | GG |
| GG | CT | GG | AA | CC | CC | GG | TT | TT | AA |
| GG | TT | GG | AA | CC | CC | GG | TT | CT | AG |
| AG | CC | AG | AA | CC | CC | AG | TT | TT | AA |
| GG | CT | AG | AA | AC | CC | GG | TT | TT | AG |
| GG | CT | GG | AA | AC | CC | GG | TT | TT | AG |
| GG | CT | AG | AA | CC | CC | GG | TT | TT | AG |
| AG | CT | AG | AA | CC | CC | GG | TT | TT | AG |
| GG | TT | AG | AG | CC | CC | GG | TT | TT | AG |
| GG | CT | AA | AA | CC | CC | GG | TT | TT | AG |
| GG | CT | AG | AA | CC | CC | GG | TT | TT | AA |
| GG | TT | AG | AA | CC | CC | GG | TT | TT | GG |
| AG | CC | AG | AG | CC | CC | GG | TT | TT | GG |
| GG | CT | GG | AA | CC | CC | GG | TT | TT | AG |
| GG | CT | AG | AA | CC | CC | GG | TT | CT | AA |
| GG | TT | AA | AA | CC | CC | GG | TT | TT | AG |
| GG | CC | AG | AA | CC | CC | GG | TT | TT | AG |
| GG | CC | AG | AA | CC | CC | GG | TT | TT | GG |
| GG | CC | AG | AA | CC | CC | GG | TT | TT | AA |
| GG | TT | AG | AA | CC | CC | GG | CT | TT | AG |
| GG | CC | GG | AA | CC | CC | GG | TT | TT | AA |
| AG | CT | AG | AG | CC | CC | GG | TT | TT | AA |
| AG | CT | GG | AA | CC | CC | GG | TT | TT | AG |
| AG | CT | GG | AA | CC | CC | GG | TT | TT | AA |
| GG | TT | AG | AA | CC | CC | GG | TT | TT | AG |
| GG | TT | AA | AA | AC | CC | GG | TT | TT | AA |
| AG | CC | AA | AA | CC | CC | GG | TT | CT | AG |
| AG | CT | AA | AG | AC | CC | GG | TT | TT | AG |
| AA | TT | GG | AG | AA | TT | GG | TC | CC | GG |
| AA | TT | GA | AA | AA | TT | GG | CC | TC | GG |
| AA | TT | GG | GG | AA | TT | GG | CC | CT | GG |
| AA | TT | GG | AG | AC | TT | GA | CT | TC | GG |
| AA | TT | GG | AA | AA | CT | AG | TC | CC | GG |
| AA | TT | AA | GA | AA | CT | GG | TC | CC | GG |
| AA | TT | AG | GG | AA | TT | GG | CC | CC | AG |
| AA | TT | GA | AA | AA | TC | AG | CC | CC | GG |
| AA | TT | GG | GA | AC | TT | GG | CT | CC | GG |
| AA | TC | GA | AA | AA | CT | GG | CC | CC | GG |
| AA | CT | AG | AG | AA | TT | GG | CC | CC | GG |
| AA | TT | AG | AG | AA | CT | GA | CC | CC | GG |
| AA | TT | AG | AA | CA | CT | GG | CC | CC | GG |
| AA | TT | AA | AA | AC | TT | GA | CC | CC | GG |
| AA | CT | GG | GA | AA | TT | GG | CC | CC | GG |
| AA | TT | GG | GA | AA | CT | GG | CC | CC | GA |
| AA | TT | AG | GG | AA | TC | GG | TC | CT | GG |
| AA | TT | GG | GA | AA | TT | GG | CC | CC | GG |
| AA | TT | GG | GA | AA | CT | GA | CT | CC | GG |
| AA | TT | AA | GG | AA | TT | GG | CC | CC | AG |
| AA | TT | AA | AG | CA | TT | GG | TC | CC | GG |
| AA | TT | AG | GA | AA | CT | GG | CT | CC | GG |
| AA | TT | GA | GA | AA | TC | GG | CC | CC | GG |
| AA | TT | AG | AG | AA | TC | GG | TC | CC | GG |
| AA | TT | GG | GA | AA | CT | GG | TC | CC | GG |
| AA | TT | GG | GA | AA | TT | GG | CC | CC | GG |
| AA | TT | AG | AG | AA | TC | GG | CC | CC | GG |
| AA | TT | AA | AA | AA | TT | GA | CC | CC | AG |
| AA | TT | GA | GA | AA | TT | GG | TC | CC | AA |
| AA | TT | AA | AG | AA | TT | GG | CC | CC | GG |
| AA | TT | AA | AA | AA | TT | GG | CC | CC | GA |
| AA | TT | GG | AA | AA | CT | GG | TC | TC | GA |
| AA | TT | GG | AA | AA | CC | GG | CC | CC | GA |
| AA | TT | AA | AG | AA | TC | GG | CC | CC | GG |
| AA | TT | AG | AA | AA | TT | GG | TC | CT | GA |
| AA | TT | GG | AG | CA | TT | GG | CT | CT | GG |
| AA | TT | GG | AA | AA | TT | GA | TC | CC | GG |
| AA | TT | GG | GG | AA | TT | GG | TC | CC | GA |
| AA | TT | AG | GG | AA | TC | GG | CC | CC | GG |
| AA | TT | GG | AG | AA | TT | GG | CT | CC | GG |
| AA | TT | GA | GA | AA | CT | GG | CC | CC | GG |
| AA | TT | AA | AG | AA | TT | GG | CC | CT | GG |
| AA | TT | GG | GA | AA | TT | AG | CT | CC | GG |
| AA | TT | GA | AA | AA | TT | GG | CC | CC | GA |
| AA | TT | GG | AG | AA | TT | GA | CC | TC | GA |
| AA | TT | GA | AA | AA | CT | GG | CC | CC | GG |
| AA | TT | AG | GA | AA | TT | GG | CT | CT | GG |
| AA | TT | GG | AG | AA | CT | GG | CC | CC | GA |
| AA | TT | AG | GA | AA | TT | GG | CT | CC | GA |
| AA | TT | GG | GA | AA | TT | GG | TC | CC | GG |
| AA | TT | GG | GA | CA | TT | GG | CC | CC | GA |
| AA | TT | GA | AA | AA | TT | GG | TC | CC | AG |
| AA | TT | AA | AA | AA | TT | AG | CC | CT | GG |
| AA | TT | GG | AA | CA | TT | GG | TC | CC | AG |
| AA | TT | AG | GA | AA | TT | GG | TC | CC | AG |
| AA | TT | AG | AA | AA | TT | GG | CC | CC | AG |
| AA | TT | GG | AA | AA | TC | GG | CC | CC | GG |
| AA | TT | AA | GA | AA | CT | GG | CC | CC | GG |
| AA | TT | AA | AG | AA | TC | GG | CC | CC | GG |
| AA | TT | GA | AG | AA | TT | GG | CC | CC | GG |
| AA | TT | AG | AG | AA | TT | GG | CC | CC | AG |
| AA | TT | AG | AG | AA | TT | GG | CC | CC | AG |
| AA | TT | GG | AA | AA | TT | GG | TC | TC | GG |
| AA | TT | GG | GA | AA | TT | GG | CC | CC | AA |
| AA | TT | AA | AG | AC | TT | GG | CC | CC | GG |
| AA | TT | AA | AG | CA | TT | GG | TC | CT | GG |
| AA | TT | AG | GA | AA | CT | GG | CC | CC | GG |
| AA | TT | AG | AG | AA | TC | GG | CC | CC | GG |
| AA | TT | AA | AA | AC | CC | GG | CC | TC | AG |
| AA | TC | AG | AA | AA | TT | GG | TC | CT | AG |
| AA | TT | GG | AG | AA | CC | GG | CC | CC | GG |
| AA | TT | AG | AA | AC | TT | GG | CC | CC | GG |
| AA | TT | AA | GA | AA | TT | GG | CC | TC | GG |
| AA | TT | GA | AG | AA | TC | GG | CC | CC | GG |
| AA | TT | GG | AA | AA | TT | AG | CC | CC | AG |
| AA | TT | AA | GA | AA | TT | GG | CC | CC | GG |
| AA | TT | AG | GG | AA | TT | GG | CC | CC | GG |
| AA | TT | GG | GA | AA | TT | GG | CC | CC | GG |
| AA | TT | AG | AG | CA | CT | GG | CT | CC | GG |
| AA | TT | AA | GA | AA | TT | GG | CC | CC | GG |
| AA | TT | GG | AA | AA | TT | GA | CC | CC | GG |
| AA | TT | AG | AA | AA | TT | GG | CC | CT | GG |
| AA | TT | GG | GA | AA | TC | GG | CC | CC | GA |
| AA | TT | GG | AA | AA | TT | GA | CC | CC | GG |
| AA | TT | AA | AA | AA | TT | GG | CC | CC | GG |
| AA | TT | GA | AA | AA | TT | AG | CC | CC | GG |
| AA | TT | AG | AG | AA | TC | GG | CC | CC | GG |
| AA | TT | GG | AA | AA | TC | GG | CC | TT | GA |
| AA | CC | AG | GG | CC | TT | GG | TT | CC | AG |
| AG | CC | GG | GG | CC | CT | GG | TT | CC | GG |
| AA | CC | GG | GG | CC | CT | GG | TT | CT | GG |
| AA | CC | GG | GG | CC | TT | GG | TT | CT | GG |
| AG | CC | GG | GG | CC | CT | GG | TT | CT | GG |
| AA | CC | GG | GG | CC | CC | GG | TT | CC | AG |
| AA | CC | GG | GG | CC | CT | GG | TT | CT | GG |
| AA | CC | GG | GG | CC | TT | GG | TT | CC | GG |
| AA | CC | GG | GG | CC | CC | GG | TT | CC | AG |
| AA | CC | GG | GG | CC | CC | GA | TT | CC | GG |
| AG | CC | GG | GG | CC | CT | GG | NN | CC | GG |
| AA | CC | GG | GG | CC | CC | GA | TT | CC | GG |
| AA | CC | GG | GG | CC | CT | GG | TT | CC | AA |
| AA | CC | GG | GG | CC | CT | GG | CT | CC | AG |
| AA | CC | GG | GG | CC | CC | GG | TT | CC | GG |
| AA | CC | GG | GG | CC | CT | GA | TT | CC | GG |
| AA | CC | GG | GG | CC | CT | GG | TT | CC | GG |
| AA | CC | GG | GG | CC | CC | GA | TT | CT | AG |
| AA | CC | GG | GG | CC | CC | GG | TT | CC | GG |
| AA | CC | GG | GG | CC | CT | GG | TT | TT | AG |
| AA | CC | GG | GG | CC | TT | GG | TT | TT | GG |
| AA | CC | GG | GG | CC | CT | GG | TT | CT | AG |
| AA | CC | GG | GG | CC | CT | GG | TT | CC | AG |
| AA | CC | GG | GG | CC | CT | GG | TT | CT | AA |
| AA | CC | GG | GG | CC | CT | GG | TT | CC | GG |
| AA | CC | GG | GG | CC | CT | GG | TT | CT | GG |
| AA | CC | GG | GG | CC | CC | GG | TT | CT | AG |
| AA | CC | GG | GG | AC | CC | GA | TT | CT | GG |
| AA | TC | GA | AA | CA | CC | GG | TT | TC | GG |
| AA | TT | GA | AA | CA | CC | GA | TC | TC | GG |
| AA | TT | GA | AA | CA | CT | GG | TC | TC | GG |
| AA | TT | GG | AG | CA | CT | GG | TC | TC | GG |
| AG | TC | GG | AG | CA | CT | GG | TC | TC | GG |
| AG | TC | GA | AG | CA | CT | GG | TC | TC | GA |
| AA | TT | GG | AA | CA | CT | GG | TC | TC | GG |
| AG | TC | GG | AG | CA | CT | GG | TC | TC | GG |
| AA | TT | GA | AG | CA | CT | GG | TC | TC | GG |
| AA | TC | GA | AG | CC | CT | GG | TC | TC | GA |
| AA | TT | GG | NN | CC | CT | GG | TT | TC | NN |
| AA | TC | GA | AA | CA | CT | GG | TC | TT | GG |
| AA | TC | GG | AG | CA | CT | GG | TC | TC | GG |
| AA | TT | GG | AG | CA | CT | GG | TC | TC | GA |
| AA | TC | GG | AG | CA | CC | GG | TC | TC | GG |
| AA | TT | GG | AA | CA | CT | GG | TC | TC | GA |
| AA | TC | GA | AA | CA | CC | GG | TC | TC | GA |
| AA | TC | GA | AG | CC | CT | GG | TC | TC | GG |
| AA | TT | GA | AA | CA | CT | GG | TC | TC | GG |
| AA | TC | GA | AG | CA | CT | GG | TC | TC | GA |
| AA | TT | GA | AG | CA | CT | GG | TC | TC | NN |
| AA | TC | GA | AA | CA | CC | GG | TC | TC | GG |
| AA | TT | GG | AG | CA | CC | GG | TC | TC | GG |
| AA | TC | GA | AG | CA | CT | GG | TC | TC | GG |
| AA | TC | GG | AG | CA | CT | GG | TC | TC | GA |
| AA | TC | GA | AG | CA | CT | NN | TC | TC | GG |
| AA | TT | GA | AA | CA | CT | GG | TC | TC | GA |
| AA | TC | GA | AG | CA | CT | GG | TC | TC | GA |

| **rs4540055** | **rs1229984** | **rs1509524** | **rs16891982** | **rs4704322** | **rs6886019** | **rs6875659** | **rs10455681** | **rs2080161** | **rs798949** |
| --- | --- | --- | --- | --- | --- | --- | --- | --- | --- |
| CT | GG | AA | CC | TC | CC | AA | AA | AA | TT |
| CC | GG | AA | CC | CC | CC | AA | AA | AA | TT |
| CT | GG | AA | CC | CC | CC | AA | AA | AA | TT |
| AT | GG | AA | CC | CC | CT | AA | AA | AA | CT |
| CC | GG | AA | CC | CC | CT | AA | AA | AA | TT |
| CC | GG | AA | CC | CC | CC | AA | AA | AA | TC |
| TT | GG | AG | CC | CC | CC | AA | AA | AA | TT |
| TC | GG | AA | CC | CC | CC | AG | AA | AA | TT |
| TC | GG | AA | CC | CC | CC | AA | AA | AA | TT |
| TT | GG | AG | CC | CC | CC | AA | AA | AA | TT |
| CC | GG | AG | CC | CC | CC | AA | AA | AA | TT |
| CT | GG | AG | CC | CC | CC | AA | AA | AA | CT |
| TC | GG | AA | CC | CC | CC | AA | AA | AA | TT |
| CC | GG | AA | CC | CT | CC | AA | AA | AA | TT |
| TT | GG | AA | CC | CT | CC | AA | AA | AA | TC |
| CT | GG | AA | CC | CC | CC | AA | AA | AA | TT |
| TT | GG | AA | CC | CC | CC | AA | AA | AA | TT |
| CT | GG | AA | CC | CC | CC | AA | AA | AC | CT |
| AA | GG | AA | CC | CC | CC | AA | AA | AA | TT |
| TC | GG | AA | CC | CC | CC | AA | AA | AA | CT |
| TT | GG | AA | CC | CC | CC | AA | AA | AA | CT |
| CT | GG | GG | CC | CC | CC | AA | AA | AA | TC |
| CC | GG | AG | CC | CC | CC | AA | AA | AC | TT |
| TT | GG | AG | CC | CC | CC | AA | AA | AA | TT |
| TC | GG | AA | CC | TC | CC | AA | AA | AA | TC |
| CC | GG | AA | CC | CC | CC | AA | AA | AA | TT |
| TC | GG | AA | CC | CC | CT | AA | AA | AA | CT |
| CC | GG | AG | CC | CC | CC | AA | AA | AA | CT |
| TT | GG | AA | CC | CC | CC | AA | AA | AA | TT |
| CT | GG | AA | CC | CC | CC | AA | AA | AA | TT |
| TC | GG | AA | CC | CC | CC | AA | AA | AA | CC |
| TC | GG | AG | CC | CC | CC | AA | AA | AA | TT |
| TC | GG | AG | CC | CC | CC | AA | AA | AA | CT |
| CC | GG | AA | CC | CC | CC | AA | AA | AA | CT |
| TT | GG | AG | CC | CC | CC | AA | AA | AA | CT |
| TA | GG | AG | CC | TC | CC | AG | AA | AA | TT |
| CC | GG | AA | CC | CC | CC | AA | AA | AA | TT |
| CC | GG | AG | CC | CC | CC | AA | AA | AA | TT |
| TC | GG | AA | CC | CC | CC | AA | AA | AA | TT |
| CC | GG | AG | CC | CC | CC | AA | AA | AA | CT |
| CC | GG | AG | CC | CC | CC | AA | AA | AA | TC |
| TT | GG | AA | CC | CC | CT | AA | AA | AA | TT |
| CC | GG | AA | CC | CC | CC | AA | AA | AA | TT |
| CC | GG | AA | CC | CT | CC | AA | AA | AA | CT |
| TC | GG | AG | CC | CC | CC | AA | AA | AA | TT |
| TT | GG | AA | CC | CC | CC | AA | AA | AA | TT |
| CC | GG | AA | CC | CC | CC | AA | AA | AA | TC |
| TT | GG | AA | CC | CC | CC | AA | AA | AA | TT |
| TT | GG | AA | CC | CC | CC | AA | AA | AA | CT |
| CT | GG | AA | CC | CC | CC | AA | AA | AA | CT |
| TT | GG | AA | CC | CC | CC | AA | AA | AA | TC |
| AC | GG | AG | CC | CC | CT | AA | AA | AA | CT |
| CC | GG | AA | CC | CC | CC | AA | AA | AA | TC |
| TC | GG | AA | CC | CC | CT | AA | AA | AA | CC |
| TC | GG | AA | CC | TC | CC | AA | AA | AA | TT |
| TA | GG | AA | CC | CC | CC | AA | AA | AA | TT |
| CC | GG | AG | CC | CC | CC | AA | AA | AA | TC |
| CT | GG | AG | CC | CC | CC | AA | AA | AA | TT |
| TT | GG | AA | CC | CC | CC | AA | AA | AA | CT |
| TC | GG | AG | CC | CC | CC | AA | AA | AA | TT |
| TC | GG | AG | CC | CC | CC | AA | AA | AA | TT |
| TC | GG | AG | CC | CC | CC | AA | AA | AA | TC |
| TT | GG | AG | CC | CC | CC | AA | AA | AA | CT |
| CC | GG | AA | CC | CC | CT | AA | AA | AA | CC |
| CC | GG | AG | CC | CT | CC | AA | AA | AA | CT |
| CA | GG | AA | CC | CC | CC | AA | AA | AA | TT |
| TC | GG | AA | CC | CC | CC | AA | AA | AA | CC |
| TT | GG | AG | CC | CC | CC | AA | AA | AA | TC |
| TT | GG | AG | CC | CC | CC | AA | AA | AA | TT |
| TT | GG | AA | CC | CC | CC | AA | AA | AA | TC |
| TC | GG | AA | CC | CC | CC | AA | AA | AA | TC |
| CT | GG | AA | CC | CC | CC | AA | AA | AA | TT |
| CC | GG | AA | CC | CC | CT | AA | AA | AA | TT |
| CT | GG | AA | CC | CC | CC | AA | AA | AA | TT |
| CT | GG | AA | CC | CT | CC | AA | AA | AA | TC |
| TC | GG | AG | CC | CC | CC | AA | AA | AA | TC |
| TC | GG | AA | CC | CC | CC | AA | AA | AA | TT |
| AT | GG | AG | CC | CT | CC | AA | AA | AA | TT |
| AC | GG | AA | CC | CC | CC | AA | AA | AA | TT |
| AT | GG | AA | CC | CC | CC | AA | AA | AA | TT |
| TT | GG | AG | CC | CC | CC | AA | AA | AA | TT |
| TT | GG | AA | CC | CC | CC | AA | AA | AA | CT |
| CT | GG | AA | CC | CC | CC | AA | AA | AA | TT |
| CT | GG | AA | CC | CT | CT | AA | AA | AA | TC |
| TT | GG | AA | CC | CC | CC | AA | AA | AA | TT |
| TT | GG | AG | CC | CT | CT | AA | AA | AA | TT |
| TC | GG | AA | CC | CC | CC | AA | AA | AA | CT |
| TC | GG | AA | CC | CC | CC | AA | AA | AA | TT |
| TA | GG | AA | CC | CC | CC | AA | AA | AA | TC |
| TA | GG | AG | CC | TC | CC | AA | AA | AA | CT |
| TT | GG | GG | CC | CC | CC | AA | AA | AC | CT |
| CC | GG | AG | CC | CC | CC | AA | AA | AA | TC |
| TT | GG | AA | CC | CC | CC | AA | AA | AA | TT |
| TC | GG | AG | CC | CC | CC | AA | AA | AA | TT |
| CT | GG | AA | CC | CC | CT | AA | AA | AA | TT |
| AC | GG | AG | CC | CC | CT | AA | AA | AA | CT |
| TT | GG | AA | CC | CT | CC | AA | AA | AA | TC |
| CT | GG | AG | CC | CC | CC | AA | AA | AA | TC |
| CC | GG | AG | CC | CT | CC | AA | AA | AC | TC |
| AT | CC | AA | CC | CC | CT | GG | GG | CC | CC |
| AT | CC | AA | CC | CC | CC | GG | AG | CC | CC |
| AT | CC | AA | CC | CT | CT | GG | GG | CC | CC |
| AA | CC | AA | CC | CC | CT | GG | AG | CC | CC |
| AT | CC | AA | CC | CT | CC | GG | AA | CC | CC |
| AT | CC | AA | CC | CC | CT | GG | AA | CC | CC |
| AT | CC | AA | CC | CT | CC | GG | AG | CC | CC |
| AT | CC | AA | CC | CT | CC | GG | AG | CC | CC |
| NN | CC | AA | CC | CT | CC | GG | AA | CC | CC |
| TT | CC | AA | CC | CT | CC | GG | AG | CC | CC |
| AT | CC | AA | CC | TT | CT | GG | AA | CC | CC |
| AT | CC | AA | CC | CC | CC | GG | AG | CC | CC |
| AT | CC | AA | CC | CC | CT | GG | AA | CC | CC |
| AA | CC | AA | CC | CT | CC | GG | AG | CC | CC |
| AA | CC | AA | CC | CC | CT | GG | AG | CC | CC |
| AT | CC | AA | CC | CC | CC | GG | AG | CC | CC |
| AT | CC | AG | CC | TT | CC | GG | AG | CC | CC |
| TT | CC | AA | CC | TT | CT | GG | GG | CC | CC |
| TT | CC | AG | CC | CT | CC | GG | AG | CC | CC |
| AT | CC | AA | CC | CC | CC | GG | GG | CC | CC |
| TT | CC | AA | CC | CT | CC | AG | AG | CC | CC |
| AA | CC | AA | GC | CC | CC | GG | AG | CC | CT |
| AT | CC | AA | CC | TT | CC | GG | AA | CC | CC |
| AT | CC | AG | CC | CT | CC | GG | AG | CC | CC |
| AA | CC | AG | CC | CT | CT | GG | AG | CC | CT |
| AA | CC | AA | CC | CC | CC | GG | GG | CC | CC |
| TT | CC | AG | CC | CT | CC | GG | AG | CC | CC |
| AA | CC | AA | CC | CC | CC | GG | GG | CC | CT |
| TT | CC | AA | CC | CT | CC | GG | GG | CC | CC |
| AT | CC | AA | CC | CC | CT | GG | GG | CC | CC |
| AA | CC | AA | CC | CC | CC | GG | GG | CC | CT |
| AT | CC | AA | CC | CT | CC | GG | AG | CC | CC |
| AT | CC | AA | CC | CT | CC | GG | AG | CC | TT |
| AT | CC | AA | CC | CC | CT | GG | AA | CC | CC |
| AA | CC | AA | CC | CT | CC | GG | AG | CC | CC |
| AT | CC | AA | CC | TT | CC | GG | AG | CC | CC |
| AA | CC | AA | CC | CC | CC | GG | AG | CC | CC |
| TT | CC | AA | CC | CT | CC | GG | AG | CC | CC |
| AT | CC | AA | CC | TT | CC | GG | AG | CC | CC |
| AT | CC | AA | CC | CT | CC | GG | AG | CC | CC |
| AA | CC | AA | CC | CT | CT | GG | AA | CC | CC |
| AA | CC | AA | CC | CC | CC | GG | GG | CC | CC |
| NN | CC | AG | CC | CT | CC | GG | GG | CC | CC |
| TT | CC | AG | CC | CT | CC | GG | AG | CC | CC |
| AT | CC | AA | CC | TT | CC | GG | AA | CC | CC |
| AT | CC | AA | CC | CT | CT | GG | AG | CC | CC |
| TT | CC | AA | CC | CC | CC | GG | AA | CC | CC |
| AT | CC | AA | NN | CT | CC | GG | AA | CC | CC |
| AT | CC | AA | CC | TT | CC | GG | AA | CC | CC |
| AA | CC | AG | CC | CT | CC | GG | GG | CC | CC |
| AA | CC | AA | CC | TT | CC | GG | AG | CC | CC |
| AA | CC | AG | CC | TT | CC | GG | GG | CC | CC |
| AA | CC | GG | CC | TT | CC | GG | GG | CC | CC |
| TT | CC | AG | CC | CT | CC | GG | AG | CC | CT |
| AT | CC | AG | CC | TT | CC | GG | AG | CC | CC |
| AA | CC | GG | CC | CC | CC | AG | GG | CC | CC |
| AT | CC | AA | CC | CT | CT | GG | AG | CC | CC |
| AA | CC | AA | CC | TT | CC | GG | GG | CC | CC |
| AA | CC | AG | CC | TT | CT | GG | AG | CC | CC |
| AA | CC | GG | GC | CT | CC | AG | GG | CC | CC |
| AA | CC | AG | CC | TT | CC | GG | AG | CC | CT |
| AA | CC | AG | CC | CC | CC | GG | GG | CC | CC |
| AA | CC | AA | CC | CT | CC | GG | GG | CC | CT |
| AT | CC | AA | CC | CT | CC | GG | AG | CC | CC |
| AA | TT | AG | CC | TT | CC | GG | GG | AA | CC |
| AA | TT | AG | CC | TT | CC | GG | GG | AA | CC |
| AA | CT | AG | CC | TT | CC | GG | GG | CC | CC |
| AC | TT | AA | CC | CT | CC | GG | GG | AC | CC |
| AA | TT | AA | CC | TT | CC | GG | GG | AC | CC |
| AA | CT | AG | CC | TT | CC | GG | GG | CC | CC |
| AA | TT | AA | CC | CT | CC | GG | GG | CC | CC |
| AA | TT | AA | CC | CT | CC | GG | GG | AC | CC |
| AA | CT | AA | CC | TT | CC | GG | GG | AC | CC |
| AA | CT | AA | CC | CC | CC | GG | GG | CC | CT |
| AA | CT | AG | CC | CT | CC | GG | GG | AC | CC |
| AA | CT | AA | CC | TT | CC | GG | GG | AA | CT |
| AA | TT | AA | CC | TT | CC | GG | AG | AC | CC |
| AA | CT | AG | CC | TT | CC | GG | GG | AC | CT |
| AA | TT | AG | CC | TT | CC | GG | GG | AA | CC |
| AA | TT | AA | CG | TT | CC | GG | GG | AA | CT |
| AC | TT | AA | CC | TT | CC | GG | AG | AC | CC |
| AA | CT | AG | CC | TT | CC | GG | AG | AA | CC |
| AA | TT | AA | CC | TT | CC | GG | GG | AA | CT |
| AA | CT | AA | CC | TT | CC | GG | GG | AC | CC |
| AA | TT | AG | CC | TT | CC | GG | GG | AA | CC |
| AA | TT | AA | CC | TT | CC | GG | AG | AA | CC |
| AA | TT | AA | CC | CT | CC | GG | AG | AC | CT |
| AA | TT | AA | CC | CT | CC | GG | GG | AA | CC |
| AA | TT | AG | CC | TT | CC | GG | GG | AC | CC |
| AA | CT | AA | CC | TT | CC | GG | AG | CC | CC |
| AA | CT | AA | CC | TT | CC | GG | GG | AC | CC |
| AA | TT | AA | CC | TT | CC | GG | GG | AA | CC |
| AA | TT | AA | CC | TT | CC | GG | GG | AA | CC |
| AA | TT | AA | CC | CT | CC | GG | GG | AC | CC |
| AA | TT | AA | CC | TT | CC | GG | GG | AC | CC |
| AC | CT | AG | CC | CT | CC | GG | GG | AC | CC |
| AA | TT | AA | CC | TT | CC | GG | GG | AA | CC |
| CC | TT | AA | CC | TT | CC | GG | AG | AC | CC |
| AA | TT | AA | CC | CT | CC | GG | GG | AC | CT |
| AA | TT | AA | CC | TT | CC | GG | GG | AC | CC |
| AA | TT | AA | CC | TT | CC | GG | GG | AA | CC |
| AA | CT | AG | CC | CT | CC | GG | GG | AA | CC |
| AA | TT | AA | CC | TT | CC | GG | AG | AC | CT |
| AA | TT | AA | CC | TT | CC | GG | GG | AC | CT |
| AC | CC | AG | CC | CT | CC | AG | AG | AC | CC |
| AA | CT | AG | CC | TT | CC | GG | GG | AA | CT |
| AA | CT | AA | CC | TT | CC | GG | AG | CC | CT |
| AA | TT | AG | CC | CT | CC | GG | GG | AC | CC |
| AA | CC | AA | CC | CC | CC | GG | GG | AA | CC |
| AA | TT | AA | CC | TT | CC | GG | GG | AC | CC |
| AA | CT | AA | CC | TT | CC | GG | GG | AA | CC |
| AC | TT | AA | CC | TT | CC | GG | AG | AA | CC |
| AA | CT | AG | CC | TT | CC | GG | GG | AC | CC |
| AC | TT | AA | CC | TT | CC | GG | AG | AA | CC |
| AA | TT | AA | CC | TT | CC | GG | GG | CC | CC |
| AC | CC | AG | CC | TT | CC | GG | GG | AC | CC |
| AA | CT | AG | CC | CT | CC | GG | GG | AC | CC |
| AC | TT | AA | CC | TT | CC | GG | AG | AA | CC |
| AA | CT | AG | CC | TT | CC | GG | GG | AA | CC |
| AA | CT | AG | CC | TT | CC | GG | GG | AC | CC |
| AA | TT | AA | CC | TT | CC | GG | GG | AC | CT |
| CC | TT | AA | CC | TT | CC | GG | GG | AA | CC |
| AA | TT | AG | CC | TT | CC | GG | AG | AA | TT |
| AA | CT | AA | CC | TT | CC | GG | GG | AC | CC |
| AA | CT | AA | CC | TT | CC | GG | AG | AC | TT |
| AA | TT | AG | CC | TT | CC | GG | GG | CC | CC |
| AA | TT | AA | CC | CT | CC | GG | AG | AA | CC |
| AA | CT | AA | CC | TT | CC | GG | GG | AA | CC |
| AA | CT | AA | CC | TT | CC | GG | AG | AC | CC |
| AA | TT | AA | CC | TT | CC | GG | GG | AA | CC |
| AA | CT | AA | CC | TT | CC | AG | GG | AA | CC |
| AA | TT | AA | CC | TT | CC | GG | GG | AC | CC |
| AA | TT | AA | CC | TT | CC | GG | GG | AC | CT |
| AA | CT | AA | CC | CT | CC | GG | AG | AA | CC |
| AC | CT | AG | CC | TT | CC | GG | GG | AA | CC |
| AA | TT | AA | CC | TT | CC | GG | GG | AA | CT |
| AC | TT | AA | CC | TT | CC | GG | GG | AA | CC |
| AA | CT | AA | CC | TT | CC | GG | GG | AC | CC |
| AA | CT | AA | CC | TT | CC | GG | GG | AA | CC |
| AA | CT | AA | CC | CC | CC | GG | GG | AC | CC |
| AA | TT | AG | CC | CT | CC | GG | GG | AA | CT |
| AA | TT | AA | CC | TT | CC | GG | GG | AC | CC |
| AA | CT | AG | CC | TT | CC | GG | GG | AA | CC |
| AA | TT | AG | CC | TT | CC | GG | AG | AC | CT |
| AA | TT | AA | CC | CT | CC | GG | GG | CC | CC |
| AA | TT | AA | CC | CT | CC | GG | GG | AC | CC |
| AA | TT | AA | CC | CT | CC | GG | GG | AA | CC |
| CC | CT | AG | CC | TT | CC | GG | GG | AC | CC |
| AA | CT | AG | CC | TT | CC | GG | GG | AA | CC |
| AA | CC | AA | CC | TT | CC | GG | AA | AA | CT |
| AA | CT | AG | CC | TT | CC | GG | AG | CC | CC |
| AA | CT | AA | CC | CT | CC | GG | AG | AC | CC |
| AC | CT | AA | CC | TT | CC | GG | GG | AC | CT |
| AA | CC | GG | GG | CT | CC | GG | AA | AA | CC |
| AA | CC | AA | GG | TC | CC | GG | AA | AA | TC |
| AA | CC | AA | GC | CC | CC | GG | AA | AA | CT |
| AA | CC | AA | GG | CC | CC | GG | AA | CA | CT |
| AA | TC | AA | GG | CC | CC | GG | GA | AA | TT |
| AA | CC | AA | GG | CT | CC | GG | AA | AA | TT |
| AA | CC | AA | GG | CC | CC | GG | AA | AA | CT |
| AA | CC | AA | GG | CC | CC | GG | AA | AA | CT |
| AA | CC | AA | GG | TC | CC | GG | AA | AA | CC |
| AA | CC | AG | GG | CC | CC | GG | AA | AA | TC |
| AA | CC | AA | GG | CC | CC | GG | GA | AC | TT |
| AA | CC | AA | GG | CC | CC | GG | GA | AA | TT |
| AA | CC | AA | GG | CC | TC | GG | AA | AA | TT |
| AA | CC | AA | GG | CC | CC | GG | AA | CA | TC |
| AA | CC | AA | GG | TC | CC | GG | AG | AA | CC |
| AA | CC | AA | GG | CC | CC | GG | AA | AC | TT |
| AA | CC | AA | GG | CC | CC | GG | AG | AA | CC |
| AA | CC | AG | GC | CC | CC | GG | AA | AA | TC |
| AA | CC | GA | GG | CC | CC | GG | AA | CA | TC |
| AA | CC | AA | GG | CC | CC | GG | AG | AA | CT |
| AA | CC | AA | GG | CC | CC | GG | AG | CA | CT |
| AC | CC | GG | GG | TC | CC | GG | AA | AA | TT |
| AA | CC | AA | GG | TC | CC | GG | AA | AA | CC |
| AA | CC | AA | GG | TT | CC | GG | AG | AC | CC |
| AA | CC | AA | GG | TC | CC | GG | GA | AC | TT |
| AA | CC | AG | GG | CC | CC | GG | AG | AC | TT |
| AA | CC | AA | GG | CC | CC | GG | AA | AA | CT |
| AA | CC | GG | GG | CC | CC | GG | AG | CA | CC |
| AA | CC | AA | CG | TC | TC | GG | AA | AC | TC |
| AA | CC | AA | GG | CC | CC | GG | GG | AA | CC |
| AA | CC | AA | GG | TC | CC | GG | GA | AA | CC |
| AA | CC | AA | GG | CC | CC | GG | AA | AA | TT |
| AA | CC | AA | GG | CC | CC | GG | AG | CA | CT |
| AA | CC | GA | GG | TC | CC | AG | AA | AA | TC |
| AA | CC | AA | GG | TC | CC | GG | AG | CA | CC |
| AA | CC | AG | GG | CC | CT | AG | AA | AA | CC |
| AA | CC | AA | GG | TC | CC | GG | AA | AA | TT |
| AA | CC | AA | GG | TC | CC | GG | AA | AA | TC |
| AA | CC | AA | GG | CT | CT | GG | AA | AA | CC |
| AA | CC | AG | GG | CC | CC | GG | AA | AA | TT |
| AA | CC | AA | GG | CC | CC | AG | AA | AC | CT |
| AA | CC | AG | GG | CC | CC | GG | AA | AA | CC |
| AA | CC | AA | GG | TC | CC | GG | AA | AA | TT |
| AA | CC | AA | GG | CC | CC | GA | AA | AA | TT |
| AA | CC | AA | GG | CC | TT | GG | AA | AA | TC |
| AA | CC | AA | GG | CC | CC | GG | GA | CA | CC |
| AA | CC | AG | GG | CC | CC | GA | AG | AA | TT |
| AA | CC | AA | GG | CT | TC | GG | AG | CC | TC |
| AA | CC | AG | GG | CC | CC | AG | AA | AA | CC |
| AA | CC | GA | GG | CC | CC | GG | AA | AC | TT |
| AA | CC | AA | GG | CC | CC | GA | AA | CC | CT |
| AA | CC | AA | GG | CC | TC | GG | AA | AC | CC |
| AA | CC | AA | GG | CC | CC | GG | AA | AC | CT |
| AA | CC | AG | GG | TC | CC | GG | AG | AA | TT |
| AA | CC | AA | GG | TC | CC | GG | AA | AA | CT |
| AA | CC | GA | GG | CC | CC | GG | AA | AA | CT |
| AA | CC | AA | GG | CT | TC | GG | AA | AA | TT |
| AA | CC | GA | GG | CC | CC | GG | AG | CA | TT |
| AA | CT | GA | GG | CC | CC | GG | AG | AA | TC |
| AA | CC | AA | GG | CC | CC | GG | AA | CA | TT |
| AA | CC | AA | GG | CC | CC | GA | AA | AC | CT |
| AA | CC | AA | GG | CC | CC | GG | AA | AA | TC |
| AA | CC | AA | GG | TC | CC | GG | AA | AA | CT |
| AA | CC | AG | GG | CT | CC | GA | AA | AA | TT |
| AA | CC | AA | GG | TT | CC | GG | AA | AA | CT |
| AA | CC | AA | GG | CC | CC | GG | AA | AA | CC |
| AA | CC | AA | GG | CT | CC | GG | AA | CC | CT |
| AA | CC | AA | GG | CT | CC | GG | AA | AA | CT |
| AA | CC | AA | GG | TC | CC | GG | AA | AA | CT |
| AA | CC | AA | GG | CT | CC | AG | AG | AC | CC |
| AA | CC | GA | GG | TC | CC | GG | AA | AC | TT |
| AA | CC | AA | GG | TC | CC | GG | AA | CA | CT |
| AA | CC | GA | GG | CC | CC | GG | AA | CA | CC |
| AA | CC | GA | GG | CC | CC | GG | AA | AA | CC |
| AA | CC | AA | GG | CC | CC | GG | AA | AA | TT |
| AA | CC | AA | GG | CC | CC | GG | AA | AA | TT |
| AA | CC | AA | GG | TC | TC | AG | AA | AA | CT |
| AA | CC | AA | GC | CC | CC | GG | GA | AA | CT |
| AA | CC | AA | GG | CC | CC | GG | AA | AA | CT |
| AA | CC | AA | GG | CT | CC | GG | AA | AA | CT |
| AA | CC | AA | GG | CC | CC | GG | AA | AA | TT |
| AA | CC | AG | GG | CC | TC | GG | AA | CA | CT |
| AA | CC | AA | GG | CT | TC | GG | AA | AA | TT |
| AA | CC | AA | GG | CC | CC | GG | AA | AA | CT |
| AA | CC | AA | GG | CC | CT | GG | AA | AA | CC |
| AA | CC | AA | GG | TC | CC | GG | GA | AC | TC |
| AA | CC | AA | GG | CT | CC | GG | AG | CA | TT |
| AA | CC | AA | GG | CT | CC | GG | AA | AA | CC |
| AA | CC | GG | CC | TT | TT | GG | GG | AA | TT |
| AT | CC | GG | CC | TT | CC | GG | GG | AA | TT |
| CT | CC | GG | CC | CC | TT | GG | GG | AA | TT |
| AT | CC | GG | CC | CC | CT | GG | GG | AA | TT |
| AT | CC | GG | CC | CT | TT | GG | GG | NN | TT |
| AT | CC | AG | CC | CT | CT | GG | AG | AA | CT |
| AT | CC | GG | CC | CT | CT | GG | GG | AC | TT |
| AC | CC | GG | CC | CC | TT | GG | GG | AA | TT |
| CC | CC | AG | CC | CC | TT | GG | GG | AC | TT |
| AC | CC | GG | CC | CC | TT | GG | GG | AA | TT |
| AA | CC | GG | CC | CT | TT | GG | GG | AA | CT |
| CC | CC | GG | CC | CT | TT | GG | GG | AC | TT |
| AC | CC | GG | CC | CT | TT | GG | GG | AC | TT |
| AA | CC | GG | CC | CT | TT | GG | GG | AA | TT |
| CT | CC | GG | CC | CC | TT | GG | GG | AA | TT |
| AC | CC | GG | CC | TT | TT | GG | GG | AC | TT |
| AC | CC | GG | CC | CC | TT | GG | GG | NN | TT |
| CC | CC | GG | CC | CC | CT | GG | GG | AA | TT |
| AA | CT | AG | CC | CC | TT | GG | GG | NN | TT |
| AA | CC | GG | NN | TT | TT | GG | GG | AA | TT |
| AT | CC | AG | CC | CT | CT | GG | GG | GG | TT |
| AT | CT | GG | CC | TT | TT | GG | GG | AA | TT |
| AT | CT | GG | CC | TT | TT | GG | GG | AA | TT |
| AA | CC | GG | NN | CT | CC | GG | GG | AA | CT |
| AA | CC | AG | CC | CT | TT | GG | GG | AC | TT |
| AT | CC | GG | CC | TT | TT | GG | GG | AA | TT |
| AA | CC | GG | CC | CT | TT | GG | GG | AC | CT |
| CT | CT | GG | CC | CC | TT | GG | GG | AA | TT |
| AA | TC | AA | CC | CC | CC | GG | AG | AC | TC |
| AA | TC | AG | CC | CC | CC | GG | AA | AA | TC |
| AT | TC | AG | CG | CC | CC | GG | AG | AA | TT |
| AT | TC | AG | CG | CT | CC | GG | AA | AA | TT |
| AT | TC | AA | CC | CT | CC | GG | AG | AC | TC |
| AA | TC | AA | CC | CC | CC | GG | AA | AA | TC |
| AC | TC | AG | CG | CT | CC | GG | AA | AC | TC |
| AT | TC | AA | CC | CT | CC | GG | AG | AC | TC |
| AA | TC | AA | CG | CT | CC | GA | AG | AA | TT |
| AA | TC | AG | CC | CC | CC | GG | AG | AA | TT |
| AA | NN | NN | CC | CC | NN | GG | NN | AA | NN |
| AA | TC | AG | CC | CT | CC | GA | AG | AC | TC |
| AT | TC | AA | CC | CC | CC | GG | AG | AC | TC |
| AC | TC | AA | CC | CT | CC | GA | AA | AA | TC |
| AC | TC | AA | CG | CC | CC | GG | AG | AC | TC |
| AA | TC | AG | CG | CT | CC | GG | AA | AC | TC |
| AC | TC | AG | CC | CC | CC | GG | AA | AC | TC |
| AT | TC | AG | CG | CT | CT | GG | AG | AA | TC |
| AC | TC | AA | CC | CC | CC | GG | AG | AA | TT |
| AT | TC | AG | CC | CC | CC | GG | AA | AA | TT |
| AA | NN | AG | CC | CT | CC | GG | NN | AC | TC |
| AA | TC | AA | CC | CC | CC | GG | AG | AC | TC |
| AT | TC | AG | CC | CC | CC | GG | AA | AC | TC |
| AT | TC | AG | CC | CC | CC | GG | AG | AA | TC |
| AT | TT | AG | CC | CT | CC | GG | AG | AC | TC |
| AT | TC | AG | CC | CC | CC | GG | AG | AA | TC |
| AC | TC | AG | CC | CT | CC | GG | AA | AA | TT |
| AT | TC | AG | CC | CT | CC | GG | AA | AA | TC |

| **rs2409722** | **rs7832008** | **rs1871534** | **rs10811102** | **rs16913918** | **rs10970986** | **rs2789823** | **rs2274636** | **rs4749305** | **rs7084970** |
| --- | --- | --- | --- | --- | --- | --- | --- | --- | --- |
| GT | AG | CC | GG | GG | TT | GG | AA | AG | CC |
| GG | GG | CC | GG | GG | TT | GG | AA | GG | CC |
| GG | AG | CC | GG | AG | TT | GG | AA | AG | CC |
| GG | GG | CC | GG | GA | TT | GG | AA | AG | CC |
| GG | AG | CC | GG | GG | TT | GG | AA | AG | CT |
| GG | GG | CC | GG | GG | TT | GG | AA | AG | CC |
| GG | AG | CC | GG | GG | TT | GG | AA | AG | CC |
| GG | GG | CC | GG | AG | TT | GG | AA | GG | CC |
| GG | AG | CC | GG | AG | TT | GG | AA | AG | CC |
| GG | GG | CC | GG | GA | TT | GG | AA | AA | CC |
| GG | AG | CC | GG | AA | TT | GG | AA | GG | CT |
| GG | AA | CC | GG | GG | TT | GG | AA | GG | CC |
| TG | AG | CC | GG | AG | TT | GG | AA | AA | CC |
| GG | GG | CC | GG | AG | TT | GG | AA | AA | CC |
| GG | AG | CC | GG | GG | TT | GG | AA | AA | CC |
| GG | GG | CC | GG | AG | TT | GG | AA | AG | CT |
| GG | AG | CC | GG | GG | TT | GG | AA | GG | CT |
| GG | AG | CC | GG | GG | TT | GG | AA | AA | CC |
| GG | GG | CC | GG | GA | TT | GG | AA | AG | CC |
| GG | GG | CC | GG | GG | TT | GG | AA | GG | CC |
| GG | AG | CC | GG | GA | TT | GG | AA | GG | TT |
| GG | GG | CC | GG | GG | TT | GG | AA | AG | CC |
| GG | AG | CC | GG | GA | TT | GG | AA | AA | CT |
| GG | AG | CC | GG | AG | TT | GG | AA | GG | CC |
| GG | AA | CC | GG | GA | TT | GG | AA | AG | CC |
| GG | AG | CC | GG | GG | TT | GG | AA | AA | CC |
| GG | GG | CC | GG | GG | TT | AG | AA | AA | CC |
| GG | AG | CC | GG | GG | TT | GG | AA | AG | CC |
| GG | GG | CC | GG | GG | TT | GG | AA | GG | CC |
| GG | AG | CC | GG | GG | TT | GG | AA | GG | CC |
| GG | AG | CC | GG | GG | TT | GG | AA | GG | CC |
| TG | GG | CC | GG | GA | TT | GG | AA | AG | CC |
| GG | AG | CC | GG | AG | TT | GG | AA | GG | CC |
| GG | AG | CC | GG | GA | TT | GG | AA | GG | CT |
| GG | AG | CC | GG | AA | TT | AG | AA | GG | CC |
| GG | AA | CC | GG | GG | TT | AG | AA | AG | CC |
| GG | AG | CC | GG | AA | TT | GG | AA | AG | CC |
| GG | AA | CC | GG | AG | TT | GG | AA | GG | CC |
| GG | GG | CC | GG | GG | TT | GG | AA | AG | CC |
| GG | AA | CC | GG | AG | TT | GG | AA | GG | CT |
| GG | AA | CC | GG | GG | TT | GG | AA | AG | CT |
| GG | GG | CC | GG | AA | TT | GG | AA | AG | CC |
| GG | AG | CC | GG | GG | TT | AG | AA | AG | CC |
| GG | AG | CC | GG | GG | TT | GG | AA | AA | CC |
| GG | GG | CC | GG | GA | TT | GG | AA | GG | CC |
| GG | AA | CC | GG | GG | TT | GG | AA | AG | CC |
| GG | AG | CC | GG | GA | TT | GG | AA | GG | CC |
| GG | AG | CC | GG | GG | TT | GG | AA | AG | CC |
| GG | AG | CC | GG | GA | TT | GG | AA | GG | CC |
| GG | AG | CC | GG | GG | TT | GG | AA | AG | CC |
| GG | GG | CC | GT | AG | TT | GG | AA | AA | CC |
| GG | AG | CC | GG | GG | TT | GG | AA | AG | CC |
| GG | AG | CC | GG | GG | TT | GG | AA | GG | CT |
| GG | AG | CC | GG | GA | TT | GG | AA | AA | CC |
| GG | GG | CC | GG | GG | TT | GG | AA | AG | CT |
| GG | AA | CC | GG | AA | TT | GG | AA | AG | CC |
| GG | AA | CC | GG | GG | TT | GG | AA | AA | CC |
| GG | GG | CC | GG | GG | TT | GG | AA | AA | CC |
| GG | GG | CC | GG | AG | TT | GG | AA | AG | CT |
| GG | AA | CG | GG | GG | TT | GG | AA | AG | CC |
| GG | AG | CC | GG | AG | TT | GG | AA | AG | CC |
| GG | GG | CC | GG | AG | TT | GG | AA | AG | CT |
| GG | AG | CC | GG | GG | TT | GG | AA | AG | CC |
| GG | AG | CC | GG | GG | TT | GG | AA | AG | CC |
| GT | GG | CC | GG | GG | TT | GG | AA | AA | CC |
| GG | AG | CC | GG | GG | TT | GG | AA | AA | CC |
| TG | AG | CC | GG | AA | TT | AG | AA | AG | CC |
| GT | AG | CC | GG | GG | TT | GG | AA | GG | TT |
| GG | AG | CC | GG | GA | TT | GG | AA | AG | CC |
| GG | GG | CC | GG | GG | TT | GG | AA | GG | CC |
| GG | AG | CC | GG | GG | TT | GG | AA | AA | CC |
| GG | AG | CC | GG | GA | TT | GG | AA | GG | CT |
| GG | AA | CC | GG | GA | TT | GG | AA | GG | CC |
| GG | AA | CC | GG | GG | TT | AG | AA | GG | CC |
| GG | AA | CG | GG | GG | TT | GG | AA | AG | CC |
| GG | AG | CG | GG | GG | TT | GG | AA | GG | CC |
| GG | AG | CC | GG | GG | TT | GG | AA | AG | CC |
| GG | AA | CC | GG | GG | TT | GG | AA | AG | CC |
| GG | AG | CG | GG | AG | TT | GG | AA | AG | CC |
| GG | GG | CC | GG | GA | TT | GG | AA | AG | CT |
| GG | AG | CC | GG | GG | TT | GG | AA | GG | CT |
| GG | AG | CC | GG | GA | TT | GG | AA | AG | CT |
| GG | AA | CC | GT | GG | TT | GG | AA | GG | CC |
| GG | AA | CC | GG | GA | TT | AG | AA | AG | CT |
| GG | GG | CC | GG | GG | TT | GG | AA | GG | CT |
| GG | AG | CC | GG | AG | TT | GG | AA | GG | CT |
| GG | AA | CC | GG | GG | TT | AG | AA | AA | CC |
| GG | AG | CC | GG | GA | TT | GG | AA | AG | CC |
| GG | GG | CC | GG | GA | TT | AG | AA | AA | CC |
| TG | AA | CC | GG | GA | TT | GG | AA | AG | CC |
| GG | AG | CC | GG | GG | TT | GG | AA | GG | CC |
| GG | AG | CC | GG | AG | TT | GG | AA | GG | TT |
| GG | GG | CC | GG | GG | TT | GG | AA | AA | CC |
| GG | GG | CC | GG | GG | TT | GG | AA | GG | CT |
| GG | GG | CC | GG | GA | TT | GG | AA | GG | CC |
| GG | AG | CC | GG | GG | TT | GG | AA | GG | CC |
| GG | AG | CC | GG | AG | TT | GG | AA | AG | CC |
| GG | AG | CC | GG | GG | TT | GG | AA | AG | CC |
| GG | AG | CC | GG | AG | TT | GG | AA | AG | CC |
| TT | AA | GG | TT | GG | TT | AA | AA | GG | CC |
| TT | AA | GG | GT | AA | CC | AA | AA | GG | CC |
| TT | AA | GG | TT | AA | TT | AA | AA | GG | CT |
| TT | AA | GG | TT | AG | TT | AA | AA | GG | CC |
| TT | AA | GG | TT | AA | TT | AA | AA | GG | CC |
| TT | AA | GG | GT | AA | TT | AA | AA | GG | CC |
| TT | AA | GG | TT | AG | TT | AA | AA | GG | CT |
| TT | AA | GG | TT | AG | TT | AA | AA | GG | CC |
| TT | AG | GG | GG | AA | TT | AA | AA | GG | CT |
| TT | AA | GG | GT | AG | TT | AA | AA | GG | CT |
| TT | AA | GG | GT | AA | CT | AA | AA | GG | CC |
| GT | AA | GG | TT | AG | TT | AA | AA | GG | CT |
| GT | GG | GG | GT | AG | TT | AA | AA | GG | CC |
| GT | AA | GG | TT | AG | TT | AA | AA | GG | CC |
| TT | AA | GG | GT | AA | CT | AA | AA | GG | CC |
| TT | AA | GG | TT | AA | TT | AA | AG | GG | CC |
| GT | AA | GG | GT | GG | TT | AA | AA | GG | CC |
| TT | AG | GG | TT | AA | CC | AA | AG | GG | CC |
| TT | AA | GG | GT | AG | CT | AA | AA | GG | CC |
| TT | AA | GG | TT | AG | CT | AA | AA | GG | CC |
| TT | AA | GG | GG | AA | CT | AA | AA | GG | CC |
| TT | AG | GG | GT | AG | TT | AA | AA | GG | CT |
| TT | GG | GG | TT | AA | TT | AA | AA | GG | CT |
| TT | AG | GG | GT | AG | CT | AA | AA | GG | CT |
| TT | AA | GG | GG | AA | CC | AA | AA | GG | CC |
| TT | AA | GG | GT | AA | CT | AA | AA | GG | CC |
| TT | AA | GG | GT | AG | TT | AA | AG | GG | CC |
| TT | AG | GG | TT | AG | CT | AA | AA | GG | CC |
| TT | AA | GG | GT | AA | TT | AA | AA | GG | CT |
| TT | AG | GG | GT | AA | TT | AA | AA | GG | CC |
| TT | AG | GG | GT | AA | TT | AA | AA | GG | CT |
| TT | AA | GG | GT | AA | TT | AA | AG | GG | CC |
| GT | AA | GG | GG | AA | CT | AA | AA | GG | NN |
| TT | AA | GG | TT | AA | CC | AA | GG | GG | CT |
| TT | AG | GG | TT | AA | CC | AA | AA | GG | CT |
| TT | AA | GG | GT | AA | TT | AA | AA | GG | CT |
| TT | AA | GG | TT | AG | CT | AA | AA | GG | CT |
| TT | AA | GG | TT | AG | CT | AA | AA | GG | CC |
| TT | AA | GG | TT | AG | CT | AA | AA | GG | CC |
| TT | AA | GG | TT | AG | CT | AA | AA | GG | CC |
| TT | AA | GG | TT | AG | CT | AA | AA | GG | CC |
| TT | AA | GG | TT | AA | TT | AA | AA | GG | CT |
| TT | AA | GG | TT | AA | TT | AA | AA | GG | CC |
| TT | AA | GG | TT | AG | CT | AA | AA | AG | CC |
| TT | AA | GG | TT | AG | CC | AA | AA | GG | CT |
| TT | AG | GG | TT | AA | TT | AA | AA | GG | CT |
| TT | AA | GG | TT | AA | CT | AA | AA | GG | CC |
| TT | AA | GG | TT | AA | TT | AA | AA | GG | CC |
| TT | AA | GG | TT | AG | CC | AA | AA | GG | CC |
| TT | AA | GG | TT | AA | CT | AA | AA | GG | CC |
| TT | AG | GG | GT | AA | TT | AA | AG | GG | CT |
| TT | AG | GG | GG | AG | TT | AA | AA | GG | CC |
| TT | AA | GG | GT | AG | TT | AA | AA | GG | CT |
| TT | AA | GG | TT | AA | TT | AA | AG | GG | CC |
| TT | AA | GG | GT | AG | CT | AA | AA | GG | CC |
| TT | AG | GG | GT | AA | TT | AA | AG | GG | CT |
| TT | AA | GG | GG | AG | CT | AA | AA | GG | NN |
| TT | AA | GG | GG | AG | CT | AA | AA | GG | CC |
| TT | AA | GG | GG | AG | CT | AA | AA | GG | CT |
| GT | GG | GG | TT | AG | TT | AA | GG | GG | CT |
| TT | AG | GG | GG | AG | TT | AA | AA | GG | CT |
| TT | AA | GG | GT | AG | CT | AA | AA | GG | CC |
| TT | AG | GG | GG | AA | CT | AA | AG | GG | CC |
| TT | AA | GG | GT | AA | TT | AA | AA | GG | CC |
| TT | AA | GG | GT | AA | TT | AA | AA | GG | CC |
| TT | AG | GG | TT | AA | TT | AA | AG | GG | CC |
| TT | GG | GG | GT | AG | TT | AA | AG | AG | CC |
| TT | AG | GG | GT | AG | TT | AA | AA | GG | CC |
| GT | AG | GG | TT | AA | TT | AA | AA | GG | CC |
| GT | GG | GG | GG | AG | CT | AA | AG | GG | CC |
| TT | AG | GG | TT | AA | CT | AA | AA | GG | CT |
| TT | AG | GG | GT | AG | TT | AA | AG | GG | CC |
| TT | GG | GG | GT | AA | CC | AA | AA | GG | CC |
| TT | AG | GG | TT | AG | CT | AA | AG | GG | CC |
| GT | AG | GG | GT | AA | TT | AA | AA | GG | CT |
| TT | GG | GG | TT | AG | TT | AA | AA | GG | CT |
| GT | GG | GG | GT | AA | TT | AA | AA | GG | CT |
| TT | GG | GG | TT | AA | CT | AA | GG | GG | CC |
| GT | AA | GG | GT | AG | TT | AA | AA | AG | CT |
| GT | GG | GG | GT | AA | TT | AA | GG | GG | CC |
| TT | AA | GG | TT | AA | TT | AA | AG | GG | CC |
| TT | AA | GG | TT | AA | CT | AA | AG | GG | CT |
| GT | AG | GG | GT | AG | CT | AA | AA | GG | CC |
| TT | AG | GG | TT | AA | TT | AA | AG | GG | CC |
| TT | AG | GG | TT | AG | TT | AA | AG | GG | CT |
| TT | GG | GG | GT | AA | TT | AA | AA | GG | CC |
| TT | AA | GG | TT | AA | TT | AA | AA | GG | TT |
| TT | AG | GG | TT | AA | TT | AA | AG | GG | CT |
| TT | AG | GG | TT | AA | CC | AA | AG | GG | CC |
| TT | GG | GG | TT | AG | TT | AA | AA | GG | CC |
| GT | GG | GG | GT | AG | TT | AA | AA | GG | CC |
| TT | GG | GG | TT | AA | CT | AA | AG | GG | CC |
| TT | AG | GG | GT | AG | TT | AA | AA | GG | CC |
| GG | GG | GG | GT | AA | CT | AA | AG | GG | CC |
| TT | AA | GG | GT | AG | TT | AA | AA | GG | CT |
| TT | AA | GG | GT | AA | CT | AA | AG | GG | CC |
| TT | GG | GG | GT | AA | TT | AA | AA | GG | CC |
| TT | AG | GG | GG | AA | TT | AA | AG | GG | CT |
| TT | AG | GG | GT | AA | TT | AA | AA | GG | CT |
| TT | AG | GG | GG | AG | TT | AA | GG | GG | CC |
| TT | AA | GG | GT | AG | CT | AA | AG | GG | CT |
| TT | AG | GG | GG | AA | TT | AA | AA | GG | CT |
| TT | GG | GG | GT | AG | TT | AA | AA | GG | CT |
| GT | AA | GG | TT | AA | CT | AA | AG | GG | CC |
| GT | GG | GG | TT | AA | CT | AA | AA | GG | CT |
| GT | AG | GG | GT | AG | CT | AA | AG | GG | CT |
| TT | GG | GG | TT | AA | TT | AA | AA | GG | CT |
| TT | AG | GG | TT | AA | CT | AA | AA | GG | CT |
| GT | AG | GG | TT | AA | CT | AA | AG | GG | CC |
| GT | AG | GG | GG | AA | CT | AA | AA | GG | CT |
| GT | GG | GG | TT | AG | CT | AA | AG | GG | CT |
| GT | AA | GG | GT | AA | CT | AA | AA | GG | CC |
| TT | AA | GG | GT | AA | TT | AA | AA | GG | CT |
| TT | AG | GG | GT | AA | CT | AA | AA | GG | CC |
| TT | GG | GG | GT | AA | TT | AA | AG | GG | CT |
| TT | AG | GG | GT | AG | CT | AA | GG | GG | CT |
| TT | AG | GG | GT | AA | TT | AA | AA | GG | CC |
| TT | AA | GG | TT | AG | TT | AA | AG | GG | CT |
| TT | AG | GG | TT | AA | CT | AA | AG | GG | CC |
| GT | AG | GG | GT | AA | CT | AA | AA | GG | CC |
| TT | AG | GG | TT | AA | CT | AA | AA | GG | CC |
| TT | GG | GG | TT | AG | TT | AA | AA | GG | CC |
| TT | GG | GG | TT | AA | TT | AA | AA | GG | CC |
| TT | GG | GG | GT | AA | TT | AA | AA | AG | CC |
| TT | GG | GG | TT | AA | TT | AA | AA | GG | CC |
| TT | AG | GG | TT | AA | TT | AA | AG | GG | CT |
| TT | AG | GG | TT | AA | TT | AA | AA | GG | CT |
| TT | GG | GG | TT | AG | CT | AA | AA | GG | CT |
| TT | AA | GG | GT | AA | TT | AA | AG | GG | CT |
| TT | AG | GG | GG | AA | TT | AA | AG | GG | CC |
| GT | AG | GG | GT | AG | TT | AA | AG | GG | CC |
| TT | AG | GG | TT | AA | TT | AA | AA | GG | CT |
| TT | GG | GG | TT | AA | TT | AA | AA | GG | CC |
| TT | AG | GG | TT | AA | TT | AA | AG | GG | TT |
| GT | GG | GG | TT | AG | TT | AA | AA | GG | CT |
| TT | GG | GG | GG | GG | TT | AA | AG | GG | TT |
| GT | AG | GG | GT | AG | CT | AA | AA | GG | CT |
| TT | AG | GG | GT | AA | CT | AA | GG | GG | CC |
| TT | GG | GG | GT | AA | TT | AA | AA | AG | CT |
| TT | AA | GG | TT | AA | CT | AA | AA | GG | CC |
| GT | AG | GG | GT | AG | TT | AA | AG | GG | CC |
| TT | GG | GG | GG | AA | CT | AA | AA | AG | CT |
| TT | AG | GG | TT | AA | TT | AA | AA | GG | CC |
| GT | AG | GG | TT | AA | TT | AA | AG | GG | CC |
| TT | GG | GG | GT | AA | TT | AA | AG | GG | CT |
| TT | AG | GG | TT | GG | TT | AA | GG | GG | CC |
| TT | GG | GG | TT | AA | TT | AA | AG | GG | CT |
| TT | GG | GG | TT | AA | TT | AA | AA | GG | TT |
| TT | AA | GG | GT | AA | TT | AA | AA | GG | CC |
| TT | GG | GG | GT | AA | CT | AA | AG | GG | CC |
| TT | AG | GG | TT | AA | CT | AA | AA | GG | CT |
| GT | AG | GG | TT | AA | TT | AA | AA | GG | CC |
| TT | AA | GG | GT | AA | CT | AA | AA | AG | CT |
| TG | AA | GG | GT | AA | TC | AA | AA | AA | TT |
| GT | AA | GG | GG | AA | TT | AA | AA | AA | TT |
| GG | AA | GG | GG | AA | CC | AA | AA | AG | TT |
| TG | AA | GG | TG | AA | TT | AA | AA | AA | TT |
| GG | AA | GG | GT | AA | TC | AA | AA | AG | TT |
| TG | AA | GG | GT | AA | TT | AA | AA | AA | TT |
| TG | GA | GG | TT | AA | TT | AA | AA | AA | TT |
| GT | AA | GG | GG | AA | CC | AA | AA | AG | TT |
| GG | AA | GG | GG | AA | CC | AA | AA | GA | TT |
| GG | AG | GG | GT | AA | TT | AA | AA | AA | TT |
| GT | AA | GG | GG | AA | TT | AA | AG | AA | TT |
| TT | AG | GG | GG | AA | TT | AA | AA | AA | TT |
| TT | AA | GG | GT | AA | TC | AA | AA | AA | TT |
| TT | AA | GG | GG | AA | TT | AA | AA | AA | CT |
| GT | AA | GG | GT | AA | CT | AA | AA | AA | TC |
| GG | AA | GG | GT | AA | CT | AA | AA | AA | TT |
| TT | AA | GG | GT | AA | TT | AA | AA | AA | TT |
| GT | AA | GG | GG | AA | TC | AA | AA | AA | TT |
| GT | AA | GG | GG | AA | TT | AA | AA | AA | TT |
| GT | GA | GG | GG | AA | TT | AA | AG | GG | TT |
| TT | AG | GG | GG | AA | TT | AA | AA | AG | TT |
| TT | AA | GG | GG | AA | TC | AA | AA | AA | TT |
| TG | GA | GG | GG | AA | TT | AA | AA | AA | TT |
| GG | AA | GG | GG | AA | CC | AA | AA | AG | TT |
| TT | AA | GG | GG | AA | TT | AA | AG | AG | TT |
| GT | AA | GG | GG | AA | TC | AA | AG | AA | TT |
| TG | AA | GG | GG | AA | TT | AA | AA | AA | TT |
| GG | AA | GG | TG | AA | TT | AA | AA | AA | TT |
| GG | GG | GG | GG | AA | TC | AA | AA | AA | CT |
| TG | AA | GG | GT | AA | TT | AA | AA | AA | TT |
| GG | AA | GG | GG | AA | TT | AA | AA | AA | TT |
| TG | AA | GG | TG | AA | TT | AA | AA | AA | TT |
| GG | GA | GG | TG | AA | CC | AA | AG | AG | TT |
| GG | AA | GG | GT | AA | TT | AA | AA | AA | TT |
| GG | AA | GG | GG | AA | TT | AA | AA | AG | TC |
| TT | GA | GG | GG | AA | TT | AA | AA | AG | TT |
| TG | GA | GG | GG | AA | CT | AA | AG | AA | TT |
| TG | AA | GG | GT | AA | CC | AA | AA | AA | TT |
| TG | AA | GG | TT | AA | TT | GA | AA | AA | TT |
| TT | AA | GG | GG | AA | CC | AA | AA | GA | TT |
| GT | AA | GG | GT | AA | CC | AA | AA | AA | TT |
| TG | AA | GG | TG | AA | TT | AA | AG | AA | TT |
| TT | AA | GG | GG | AA | TT | AA | AA | AA | TT |
| TG | AG | GG | TG | AA | CT | AA | AA | AA | TT |
| TG | AA | GG | TG | AA | TC | AA | AA | AA | TT |
| GT | AA | GG | GT | AA | TC | AA | AA | AA | TT |
| GG | AA | GG | GG | AA | CT | AA | AA | AA | TT |
| TT | AA | GG | GG | AA | TT | AA | GA | AA | CT |
| GG | AA | GG | GG | AA | TT | AA | AA | AA | TT |
| GT | AA | GG | GG | AA | TT | AA | AA | AA | TT |
| TG | GA | GG | GG | AA | CC | AA | AA | AA | TT |
| TT | AA | GG | GG | AA | CT | AA | AA | GA | TT |
| GT | AA | GG | GG | AA | TT | AA | AA | AA | TT |
| GT | GA | GG | GG | AA | TT | AA | AG | AA | TT |
| GG | AA | GG | GG | AA | TT | AA | AA | GA | TT |
| GG | AA | GG | TT | AA | TT | AA | AA | GA | TT |
| TG | GA | GG | TG | AA | TT | AA | AA | AA | TT |
| GG | AA | GG | GG | AA | CT | AA | AA | AA | TT |
| GG | AA | GG | GT | AA | TT | AA | AA | GA | TT |
| GT | AG | GG | GG | AA | TT | AA | AA | AA | TT |
| TG | AG | GG | GG | AA | TT | AA | AA | AA | TC |
| GT | AA | GG | GG | AA | TT | AA | AA | AA | TT |
| GG | AA | GG | GT | AA | TC | AA | AA | AA | TT |
| GT | AG | GG | GG | AA | TC | AA | AA | AG | TT |
| TT | AA | GG | GT | AA | TT | AA | AG | AA | TT |
| GT | AA | GG | TG | AA | TT | AA | GA | AG | TT |
| GG | AG | GG | GG | AA | TT | AA | AA | AA | TT |
| TG | AA | GG | TT | AA | TC | AA | GA | AA | TT |
| TG | AA | GG | GT | AA | TC | AA | GA | AG | TT |
| TT | AA | GG | GG | AA | CT | AA | AA | AA | TT |
| TT | AA | GG | GG | AA | CC | AA | AA | AA | TT |
| TT | GA | GG | GG | AA | TC | AA | GA | AA | TT |
| GT | GA | GG | GT | AA | TC | AA | AA | AA | TT |
| TG | AA | GG | TG | AA | TT | AA | AA | AA | TT |
| GG | AG | GG | TG | AA | TC | AA | AA | AA | TT |
| TG | GG | GG | GG | AA | TT | AA | AG | GA | TT |
| GT | AG | GG | GG | AA | TT | AA | GA | AA | TT |
| TG | AA | GG | TT | AA | TC | AA | AA | GG | TT |
| GT | AA | GG | TG | AA | CT | AA | AG | GA | TT |
| GG | AA | GG | GG | AA | TT | AA | AG | AA | CT |
| GT | AA | GG | GG | AA | TC | AA | AA | AA | TT |
| TT | AA | GG | TT | AA | CT | AA | AA | GA | TT |
| GG | AG | GG | GG | AA | TT | AA | AA | GA | TT |
| GG | AA | GG | GG | AA | CT | AA | AA | AG | TT |
| GT | GG | GG | GG | AA | CC | AA | GA | AA | TC |
| TT | AA | GG | GG | AA | CT | AA | AA | GG | TT |
| GG | AA | GG | GG | AA | TT | AA | AA | AA | TT |
| TT | AA | GG | GT | AA | TT | AA | AA | AA | TT |
| TT | GG | GG | TT | AA | CC | AA | AG | GG | TT |
| TT | GG | GG | TT | AA | CC | AA | GG | GG | TT |
| TT | GG | GG | TT | AA | CC | AA | GG | GG | TT |
| TT | GG | GG | TT | AA | CC | AA | GG | GG | TT |
| TT | GG | GG | TT | AA | CC | AA | GG | GG | CT |
| TT | GG | GG | TT | AA | CC | AA | AG | GG | TT |
| TT | GG | GG | TT | AA | CC | AA | GG | GG | TT |
| TT | GG | GG | TT | AA | CC | AA | GG | GG | TT |
| TT | GG | GG | TT | AA | CC | AA | GG | GG | TT |
| TT | GG | GG | TT | AA | CC | AA | GG | GG | TT |
| TT | GG | GG | TT | AA | CC | AA | GG | GG | TT |
| TT | GG | GG | TT | AA | CC | AA | AG | AG | TT |
| TT | GG | GG | TT | AA | CC | AA | AG | GG | TT |
| TT | GG | GG | TT | AA | CC | AA | GG | GG | TT |
| TT | GG | GG | TT | AA | CC | AA | GG | GG | TT |
| TT | GG | GG | TT | AA | CC | AA | GG | AG | CT |
| TT | GG | GG | TT | AA | CC | AA | AG | GG | TT |
| TT | GG | GG | TT | AA | CC | AA | GG | GG | TT |
| TT | GG | GG | TT | AA | CC | AA | GG | GG | CT |
| TT | GG | GG | TT | AA | CC | AA | GG | GG | TT |
| TT | GG | GG | TT | AA | CC | AA | GG | GG | CT |
| TT | GG | GG | TT | AA | CC | AA | GG | GG | TT |
| TT | GG | GG | TT | AA | CC | AA | GG | GG | CC |
| TT | GG | GG | GT | AA | CT | AA | GG | GG | CC |
| TT | GG | GG | TT | AA | CC | AA | GG | GG | CT |
| TT | GG | GG | TT | AA | CC | AA | AG | GG | CT |
| TT | GG | GG | TT | AA | CC | AA | GG | GG | CT |
| TT | GG | GG | TT | AA | CC | AA | GG | GG | CT |
| TG | GA | GG | GT | AA | TT | GA | AA | AG | CT |
| TT | GA | GG | GG | AA | TT | GA | AA | AG | CT |
| TG | GA | GC | GT | AA | TT | GA | AA | AA | CT |
| TT | GA | GG | GT | AA | TC | GA | AG | AA | CT |
| TT | GA | GG | GT | AA | TT | GA | AA | AG | CT |
| TT | GA | GG | GT | AA | TC | GA | AA | AA | CT |
| TG | GA | GG | GG | AA | TT | GA | AG | AG | CT |
| TT | GA | GG | GT | AA | TT | GA | AA | AG | CT |
| TG | GA | GG | GG | AA | TT | GA | AA | AA | CT |
| TG | GA | GG | GG | AA | TT | GA | AG | AG | CT |
| TT | GG | GG | GG | AA | NN | GA | NN | NN | CC |
| TT | GA | GG | GG | AA | TT | GA | AA | AA | CT |
| TG | GA | GG | GG | AA | TT | GA | AA | AA | CT |
| TG | GA | GG | GT | AA | TC | GA | AA | AA | CT |
| TG | GA | GG | GT | AA | TT | GA | AG | AG | CT |
| TG | GA | GG | GG | AA | TT | GA | AA | AA | CT |
| TG | GA | GG | GT | AG | TT | GA | AA | AG | CT |
| TG | GA | GG | GG | AA | TT | GA | AA | AA | CT |
| TG | GA | GG | GT | AA | TC | GA | AA | AA | CT |
| TG | GA | GG | GG | AA | TT | GA | AA | AG | CT |
| TG | GA | NN | GT | AA | TT | GA | AA | AA | CT |
| TG | GG | GG | GG | AA | TC | GA | AA | AA | CT |
| TG | GA | GG | GT | AA | TT | GA | AG | AG | CT |
| TT | GA | GG | GT | AA | TC | GA | AG | AA | CT |
| TT | GA | GG | GG | AA | TT | GA | AA | AA | CT |
| TG | GA | GG | GT | AA | TC | GA | AA | AA | CT |
| TG | GA | GG | GT | AA | TC | GA | AA | AG | CT |
| TG | GA | GG | GG | AA | TC | GA | AA | AG | CT |

| **rs3751050** | **rs5030240** | **rs174570** | **rs1924381** | **rs721367** | **rs9522149** | **rs10483251** | **rs7151991** | **rs12434466** | **rs730570** |
| --- | --- | --- | --- | --- | --- | --- | --- | --- | --- |
| TT | CC | CC | TT | CC | TT | GG | GG | AA | GG |
| TT | CA | CC | TT | CC | TT | GG | GG | AA | AG |
| TT | CC | CC | TT | CC | TT | GT | AG | AA | GG |
| TT | GA | CC | TT | CC | TT | GG | GG | AA | GG |
| TT | CA | CC | TT | CC | TT | GG | GG | GG | GG |
| TT | AG | CC | TT | CC | TT | GG | AG | AA | GG |
| TT | AC | CC | TT | CC | TT | GG | GG | AA | GG |
| TT | AC | CC | TT | CC | TT | GG | GG | AA | GG |
| TT | CA | CC | TT | CC | TT | GG | GG | AA | AG |
| TT | GA | CC | TT | CC | TT | GG | GG | AA | GG |
| TT | CC | CC | TT | CC | TT | GT | AG | AA | GG |
| TT | CA | CC | TT | CC | TT | GG | GG | AA | GG |
| TT | GC | CC | TT | CC | TT | GG | GG | AA | AA |
| CT | AC | CC | TT | CC | TT | GG | GG | AA | GG |
| TT | GC | CC | TT | CC | TT | GG | GG | AA | GG |
| TT | AG | CC | TT | CC | TT | GG | GG | AA | GG |
| TT | GG | CC | TT | CC | TT | GG | AG | AA | GG |
| TT | GA | CC | TT | CC | TT | GG | AG | GA | GG |
| TT | AC | CC | TT | CC | TT | GT | GG | AA | AG |
| CT | AC | CC | TT | CC | TT | GG | GG | AA | GG |
| TT | AG | CC | CT | CC | TT | GG | AA | AA | AG |
| TT | AA | CC | TT | CC | TT | GG | GG | AA | GG |
| TT | GG | CC | CT | CC | TT | GG | GG | AA | GG |
| TT | CC | CC | TT | CC | TT | GG | GG | AA | AG |
| TT | AC | CC | TT | CC | TT | GG | GG | AA | GG |
| CT | GA | CC | TT | CC | TT | GG | AA | AG | AA |
| TT | AG | CC | TT | CC | TT | GG | GG | AA | AG |
| TT | AC | CC | TT | CC | TT | GG | GG | AA | GG |
| CT | AG | CC | TT | TC | TT | GG | AG | AA | GG |
| TT | AG | CC | TT | CC | TT | GG | AG | AA | GG |
| CT | GA | CC | TT | CC | TT | GG | GG | GA | GG |
| TT | GA | CC | TT | CC | TT | GG | AG | AG | GG |
| TT | GC | CC | TT | CC | TT | GG | GG | AA | GG |
| TT | GA | CC | TT | CC | TT | GG | GG | AA | GG |
| TT | GA | CC | CT | CC | TT | GG | GG | AA | GG |
| CT | CC | CC | TT | CC | TT | GG | GG | AA | GG |
| TT | AA | CC | CT | CT | TT | GG | GG | AA | GG |
| TT | GC | CC | TT | CC | TT | GG | GG | AG | GG |
| TT | AA | CC | TT | CC | TT | GG | AG | AA | GG |
| TT | CA | CC | TT | CC | TT | GG | GG | AA | GG |
| TT | CC | CC | TT | CC | TT | GT | GG | AA | AG |
| TT | GA | CC | TT | CC | TT | GG | AG | AA | GG |
| TT | AG | CC | TT | CC | TT | GG | GG | AA | AG |
| TT | CG | CC | TT | CC | TT | GG | GG | AA | GG |
| TT | CC | CC | TT | CC | TT | GG | AG | AA | GG |
| TT | GG | CC | TT | CC | TT | GG | GG | AG | AG |
| TT | CA | CC | TT | CC | TT | GG | GG | AA | AG |
| TT | AG | CC | TT | CC | TT | GG | GG | AA | AG |
| TT | AG | CC | CT | CC | TT | GG | GG | AA | GG |
| TT | AC | CC | CT | CC | TT | GG | GG | AA | AG |
| TT | AG | CC | TT | CC | TT | GG | AG | AA | AG |
| CT | GA | CC | TT | CC | TT | GG | GG | AA | AG |
| TT | CC | CC | TT | CT | TT | GG | GG | AA | AG |
| TT | CG | CC | TT | CC | TT | GG | GG | AA | GG |
| TT | CC | CC | CT | CC | TT | GG | AG | AA | AG |
| TT | CA | CC | TT | CT | TT | GG | GG | AA | GG |
| TT | CA | CC | TT | CC | TT | GT | AG | AA | AG |
| TT | CC | CC | TT | CC | TT | GG | GG | AG | GG |
| TT | GC | CC | TT | CC | TT | GG | GG | AA | AG |
| TT | AG | CC | TT | CT | TT | GG | GG | AA | GG |
| TT | AC | CC | TT | CC | TT | GG | GG | AA | GG |
| TT | GC | CC | TT | CC | TT | GG | GG | AA | GG |
| TT | AA | CC | TT | CC | TT | GT | GG | GA | AA |
| TT | CG | CC | TT | TC | TT | GT | AG | AA | AA |
| TT | AG | CC | TT | TC | TT | GG | GG | AA | GG |
| CT | AC | CC | TT | CT | TT | GG | AG | AA | GG |
| TT | CG | CC | TT | CC | TT | GT | GG | AA | GG |
| CT | GC | CC | TT | CC | TT | GG | GG | AA | GG |
| TT | AG | CC | TT | CC | TT | GG | AG | AA | AG |
| TT | CC | CC | TT | CC | TT | GG | GG | AA | AG |
| TT | CG | CC | TT | CC | TT | GG | GG | AA | AG |
| TT | GG | CC | TT | CC | TT | GG | AA | AG | AG |
| TT | GG | CC | TT | CC | TT | GT | AG | GA | AG |
| TT | AA | CC | TT | CC | TT | GG | GG | AA | AG |
| TT | CG | CC | TT | CC | TT | GG | AG | AA | AG |
| TT | GA | CC | TT | CC | TT | GG | AG | GA | GG |
| TT | GC | CC | TT | CC | TT | GG | GG | GA | GG |
| TT | GA | CC | TT | CC | TT | GG | GG | GA | GG |
| TT | CG | CC | TT | CC | TT | GG | GG | AA | AG |
| CT | GA | CC | TT | TC | TT | GG | GG | AA | AA |
| TT | GC | CC | TT | CT | TT | GG | GG | AA | GG |
| CC | AG | CC | TT | CC | TT | GG | GG | AA | GG |
| TT | AA | CC | TT | CC | TT | GT | GG | AA | AG |
| TT | AA | CC | TT | CC | TT | GG | GG | AA | AG |
| CT | CG | CC | TT | CC | TT | GG | AG | AA | GG |
| TT | GA | CC | TT | CC | TT | GG | GG | AG | GG |
| CT | CC | CC | TT | TC | TT | GG | GG | AA | AG |
| TT | CA | CC | TT | CC | TT | GG | GG | GA | AG |
| TT | GG | CC | TT | CT | TT | GG | GG | AA | AG |
| TT | AC | CC | CT | CC | TT | GG | GG | AA | AG |
| TT | AA | CC | TT | CC | TT | GG | GG | AA | AG |
| TT | CA | CC | TT | CC | TT | GT | AG | AA | AG |
| TT | GG | CC | TT | CC | TT | GG | GG | AA | GG |
| TT | GG | CC | TT | CC | TT | GG | GG | AA | GG |
| TT | CC | CC | TT | CC | TT | GG | AG | AA | AG |
| TT | AA | CC | TT | CC | TT | GG | AG | AA | AG |
| TT | CG | CC | TT | CC | TT | GG | GG | AA | AG |
| TT | AA | CC | TT | CC | TT | GG | GG | AG | GG |
| TT | CC | CC | TT | CC | TT | GG | GG | AG | GG |
| TT | GG | TT | CT | TT | TT | GT | AA | NN | AA |
| TT | GG | CT | CC | CT | TT | TT | AA | NN | AA |
| TT | CG | TT | TT | TT | TT | TT | AA | NN | AA |
| TT | CG | TT | TT | CT | TT | TT | AA | NN | AA |
| TT | GG | TT | TT | CT | TT | TT | AA | NN | AG |
| TT | GG | TT | CT | CT | TT | TT | AA | NN | AG |
| TT | GG | TT | CT | CT | TT | TT | AA | NN | AA |
| TT | GG | TT | CT | TT | TT | NN | AA | NN | AA |
| TT | GG | TT | TT | CT | TT | TT | AA | NN | AA |
| TT | GG | TT | CT | CC | TT | TT | AA | NN | AA |
| TT | CG | TT | CT | CC | TT | TT | AA | NN | AA |
| TT | GG | TT | TT | CT | TT | TT | AA | NN | AA |
| TT | GG | TT | CT | TT | TT | GT | AA | NN | AA |
| TT | GG | TT | CC | CT | TT | TT | AA | NN | AG |
| TT | CG | TT | TT | CT | TT | TT | AA | NN | AG |
| TT | AC | TT | CT | CC | TT | TT | AA | NN | AA |
| TT | CC | TT | CT | CT | TT | TT | AA | NN | AG |
| TT | GG | CT | TT | CT | TT | TT | AA | NN | GG |
| TT | CC | TT | CT | CT | TT | TT | AA | NN | GG |
| TT | CG | TT | CT | CC | TT | TT | AA | NN | GG |
| TT | CG | CT | CT | CC | TT | TT | AG | NN | GG |
| TT | CG | TT | CT | CC | TT | TT | AG | NN | GG |
| TT | CG | CT | TT | CT | TT | TT | AA | NN | AG |
| TT | AC | TT | CT | CC | TT | TT | AG | NN | AA |
| TT | CG | TT | CT | CT | TT | TT | AA | NN | AA |
| TT | GG | TT | CT | CT | TT | TT | AA | NN | AA |
| TC | CG | CT | CT | CT | TT | TT | AA | NN | GG |
| TT | GG | TT | TT | CC | TT | TT | AA | NN | GG |
| TT | AC | TT | TT | CC | TT | TT | AG | NN | AG |
| TT | CG | CT | CT | CC | TT | TT | GG | NN | GG |
| TT | CG | TT | TT | CT | TT | TT | AA | NN | GG |
| TC | GG | TT | CC | CC | TT | TT | AA | NN | GG |
| TC | CG | TT | CT | CT | TT | TT | AA | NN | AA |
| TT | CG | TT | TT | CC | TT | TT | GG | NN | AA |
| TT | CG | TT | TT | CT | TT | TT | AA | NN | AG |
| TT | GG | TT | CT | CT | TT | TT | AA | NN | AA |
| TT | GG | TT | TT | CT | TT | TT | AA | NN | GG |
| TT | GG | TT | TT | CT | TT | TT | AA | NN | AG |
| TT | GG | TT | TT | CC | TT | TT | AA | NN | GG |
| TT | GG | TT | CT | TT | TT | TT | AA | NN | GG |
| TT | GG | TT | TT | TT | TT | TT | AA | NN | GG |
| TT | GG | TT | CT | CT | TT | TT | AA | NN | GG |
| TT | GG | TT | CT | CC | TT | TT | AA | NN | AA |
| TT | GG | TT | CT | CT | TT | TT | AA | NN | GG |
| TT | GG | TT | CT | CT | TT | TT | AA | NN | GG |
| TT | GG | TT | CT | CT | TT | TT | AA | NN | GG |
| TT | GG | TT | TT | TT | TT | TT | AA | NN | AG |
| TT | GG | TT | TT | CT | TT | TT | AA | NN | GG |
| TT | GG | TT | CT | CC | TT | TT | AA | NN | GG |
| TT | GG | TT | TT | TT | TT | TT | AA | NN | AA |
| TT | CC | TT | CT | TT | TT | TT | AG | NN | AA |
| TT | CG | TT | CT | CT | TT | TT | AA | NN | GG |
| TT | GG | TT | TT | TT | TT | TT | AA | NN | GG |
| TT | CG | TT | CT | CT | TT | TT | AA | NN | GG |
| TT | CC | TT | CT | CT | TT | TT | GG | NN | AG |
| TT | CG | TT | CT | CT | TT | TT | AA | NN | GG |
| TT | CG | TT | TT | CT | TT | TT | AG | NN | AG |
| TT | GG | TT | CT | CC | TT | TT | AG | NN | GG |
| TT | GG | TT | TT | TT | TT | TT | AA | NN | GG |
| TT | CG | TT | CT | CC | CT | TT | AA | NN | GG |
| TT | CG | TT | TT | CC | CT | GT | AA | NN | GG |
| TT | CG | TT | TT | CT | CT | TT | GG | NN | AA |
| TC | GG | TT | TT | CC | TT | TT | AA | NN | AG |
| TC | GG | TT | TT | CT | TT | TT | AG | NN | GG |
| TT | CC | CC | TT | CT | TT | GG | AG | GG | GG |
| TT | CC | CT | TT | TT | TT | GG | GG | AG | GG |
| TT | AC | CC | TT | CT | TT | GG | GG | AG | AG |
| CT | CC | CT | CT | TT | TT | GT | GG | GG | GG |
| CT | AC | CC | TT | TT | TT | GG | GG | AG | GG |
| TT | CC | CT | CT | TT | TT | GG | GG | AG | GG |
| TT | CC | CT | TT | CC | TT | GG | GG | AG | GG |
| TT | CC | CT | TT | CT | TT | GT | GG | AG | GG |
| TT | CC | CC | TT | CT | TT | GG | GG | GG | GG |
| TT | CC | CC | TT | CC | TT | GG | GG | GG | GG |
| TT | CC | CC | TT | CT | TT | GG | GG | GG | GG |
| TT | CC | CT | CT | CT | TT | GG | GG | GG | AG |
| CT | CC | CC | CT | CC | TT | GG | GG | GG | GG |
| TT | CC | CT | TT | CT | TT | GG | AG | GG | GG |
| TT | CC | TT | TT | CT | TT | GG | AG | GG | GG |
| TT | CC | CC | TT | CC | TT | GG | GG | AA | GG |
| TT | CC | CT | TT | CT | TT | GG | GG | GG | AG |
| TT | CC | CC | CT | CT | TT | GG | AG | GG | GG |
| TT | AC | CT | TT | CT | TT | GG | GG | GG | GG |
| TT | CC | CT | CT | TT | TT | GG | GG | AG | GG |
| TT | CC | CC | CT | TT | TT | GG | GG | GG | AG |
| TT | CC | CT | CT | TT | TT | GG | GG | AG | GG |
| TT | CC | CT | TT | TT | TT | GG | GG | GG | GG |
| TT | CC | CT | CT | TT | TT | GG | AG | AG | GG |
| TT | CC | TT | CT | CC | TT | GG | GG | GG | GG |
| TT | CC | CC | CT | CT | TT | GG | GG | GG | GG |
| TT | CC | CC | TT | CC | TT | GG | GG | GG | GG |
| TT | CC | CT | TT | CT | TT | GG | AG | GG | GG |
| TT | AC | CC | TT | TT | TT | GG | GG | GG | AG |
| TT | AC | CT | TT | CT | TT | GG | AG | GG | AG |
| TT | CC | CC | CT | CC | TT | GG | GG | GG | GG |
| TT | CC | CT | TT | TT | TT | GG | AG | AG | GG |
| TT | CC | CC | TT | CT | TT | GG | GG | GG | GG |
| TT | CC | CT | CT | CT | TT | GG | AG | AG | GG |
| TT | CC | CC | CT | CT | TT | GG | AG | AA | AG |
| TT | CC | CT | CT | TT | TT | GG | GG | GG | GG |
| TT | CC | TT | TT | CT | TT | GG | GG | AG | GG |
| TT | AC | TT | TT | CT | TT | GG | GG | GG | GG |
| TT | CC | TT | CT | TT | TT | GG | AG | AG | GG |
| TT | AC | CC | CT | TT | TT | GT | GG | GG | GG |
| TT | CC | CT | TT | TT | TT | GG | AG | GG | GG |
| TT | CC | CC | TT | TT | TT | GG | GG | AG | GG |
| TT | CC | CC | TT | CT | TT | GG | GG | GG | GG |
| TT | CC | CT | CC | CT | TT | GG | AG | GG | GG |
| TT | CC | CC | TT | TT | TT | GG | GG | GG | GG |
| TT | CC | CT | CT | TT | TT | GG | AG | GG | AG |
| TT | CC | CC | TT | CT | TT | GG | GG | GG | AG |
| TT | CC | CC | CT | TT | TT | GG | AG | GG | AG |
| TT | CC | CT | CT | CT | TT | GG | GG | AG | GG |
| TT | CC | TT | TT | CT | TT | GG | GG | GG | GG |
| TT | CC | CT | CT | TT | TT | GG | AG | AA | GG |
| CT | CC | CC | TT | CT | TT | GG | GG | GG | GG |
| TT | CC | CC | TT | TT | TT | GG | GG | GG | GG |
| TT | CC | CT | CT | TT | TT | GG | GG | GG | GG |
| CT | CC | CC | TT | TT | TT | GG | GG | GG | GG |
| TT | CC | TT | TT | TT | TT | GG | GG | AG | GG |
| TT | CC | CC | TT | TT | TT | GG | AG | GG | GG |
| TT | CC | CC | TT | CT | TT | GG | GG | GG | GG |
| TT | CC | CC | TT | CT | TT | GG | GG | GG | GG |
| TT | CC | CT | TT | TT | TT | GG | GG | GG | GG |
| TT | CC | TT | CT | TT | TT | GG | GG | AG | GG |
| TT | AC | TT | TT | TT | TT | GG | GG | GG | AG |
| TT | CC | CC | CT | TT | TT | GG | GG | GG | GG |
| TT | CC | CC | CT | TT | TT | GG | GG | GG | GG |
| TT | CC | CC | TT | CC | TT | GT | GG | GG | GG |
| TT | CC | CC | CT | CT | TT | GT | AG | GG | GG |
| TT | CC | CT | TT | TT | TT | GG | GG | AG | GG |
| TT | CC | CT | TT | CT | TT | GG | AG | GG | GG |
| TT | CC | CC | CC | TT | TT | GG | GG | GG | GG |
| CT | CC | CC | TT | CT | TT | GG | GG | GG | GG |
| TT | CC | TT | TT | CT | TT | GG | GG | AG | GG |
| TT | CC | CT | CT | CT | TT | GG | AG | GG | GG |
| TT | CC | CT | TT | TT | TT | GG | AG | GG | GG |
| TT | CC | CT | TT | CC | TT | GT | AG | GG | GG |
| TT | CC | CT | TT | TT | TT | GG | GG | GG | GG |
| TT | CC | CC | CT | CT | TT | GG | GG | AG | GG |
| TT | CC | CC | TT | TT | TT | GG | GG | AG | GG |
| TT | AC | CC | TT | TT | TT | GG | AG | GG | GG |
| TT | CC | CT | TT | CT | TT | GG | GG | AG | GG |
| TT | AC | CC | CT | TT | TT | GG | GG | GG | GG |
| TT | CC | CC | TT | TT | TT | GG | GG | GG | AG |
| TT | CC | CC | TT | CT | TT | GT | GG | AA | AG |
| TT | CC | CC | TT | TT | TT | GG | AG | GG | GG |
| TT | CC | CT | TT | CT | TT | GT | GG | GG | GG |
| TT | AC | CT | TT | CT | TT | GG | AG | GG | GG |
| CT | CC | CT | CT | CT | TT | GT | AG | GG | GG |
| TT | CC | CT | TT | TT | TT | GG | GG | GG | AG |
| TT | CC | CC | CT | CT | TT | GG | GG | AA | GG |
| TT | CC | CT | TT | TT | TT | GG | AG | GG | GG |
| TT | CA | CC | CC | CC | CT | TG | AG | AA | AA |
| TT | CC | CC | CC | CC | TC | GG | GA | AA | GA |
| TT | CC | CC | CC | CC | CT | GT | GG | AA | AG |
| TT | AC | CT | CT | CC | CC | GG | GG | AG | AA |
| TT | CC | CC | CC | CC | CC | GG | GG | AA | AG |
| TC | CC | CC | CC | CC | CC | GG | GA | AA | AA |
| TT | CC | CC | CC | CC | TC | GT | GG | AA | AG |
| TT | CC | CC | CC | CC | CT | GG | GG | AA | AA |
| TT | AA | CC | CC | CC | CT | TG | GG | AA | AA |
| TT | CC | CT | CC | CC | CC | GG | AG | AA | AA |
| CT | CC | CT | CC | CC | CC | GG | GG | AA | AA |
| TT | CC | CC | CC | CC | TC | GG | GG | AA | GA |
| TT | CC | CC | CC | CC | CC | GG | AA | AA | AA |
| CT | CC | TC | CC | CC | CT | GG | GG | AA | AG |
| TT | CC | CT | TC | CC | CC | GG | AG | AA | AA |
| TT | CC | CT | CC | CC | CC | GT | GG | AA | AA |
| TT | CC | TC | CC | CC | CC | GG | GG | AA | AA |
| TT | CC | CC | CC | CC | CC | GG | GG | AA | AA |
| TT | CC | TC | TC | CC | CC | GG | AA | AA | AA |
| TT | CC | CC | CC | CC | CC | GT | GG | GA | AA |
| TT | CC | CC | CC | CC | CC | GG | GG | AA | AA |
| TT | CC | CC | CC | CC | CC | GT | GA | AA | GA |
| TT | CC | CC | CC | CC | CC | GT | GG | AA | AA |
| TC | CC | CC | TC | CC | CC | GG | GG | AA | AA |
| TT | CC | CC | CC | CC | TT | GT | GG | AA | AA |
| TT | CC | CC | CT | CC | TC | GG | GG | AA | GA |
| TT | CC | CT | CC | CC | TC | GG | GG | AA | AA |
| TC | CC | CC | CT | CC | CC | TG | GG | AA | AA |
| TC | CC | CC | CC | CC | CC | GG | GG | AA | AA |
| TC | CC | TC | CC | CC | CC | GG | GG | AA | AA |
| TT | AC | CC | CC | CC | TC | GG | GA | AA | AA |
| TT | CC | CC | CT | CC | CC | GG | GA | AA | AA |
| TT | CC | CC | TC | CC | TC | GT | GG | AA | AA |
| CT | CC | CC | CC | CC | TC | GG | GA | AA | AG |
| TT | CA | CC | CC | CC | TC | GT | GG | AA | AA |
| TT | CC | CC | CC | CC | TT | TG | AG | AA | GG |
| TT | AC | CC | CC | CC | TC | GG | GG | AA | GA |
| TT | CC | CC | CC | CC | CT | GT | GG | AA | GA |
| TT | CA | CC | CT | CC | CT | TG | GA | AA | AA |
| TT | CC | CC | CC | CC | CT | GG | GG | AA | AA |
| TT | CC | CT | CC | CC | CC | TG | GG | AA | AG |
| TT | CC | TC | CC | CC | TC | GG | GG | AA | AG |
| TT | AC | CC | TC | CC | TT | GG | GG | AA | AA |
| TT | CC | CC | CC | CC | CC | TG | AG | AA | AA |
| TT | CC | TC | CC | CC | CC | GT | AA | AA | AA |
| TT | CC | CC | CC | CC | TC | TG | GG | AA | GA |
| TT | CA | TC | CC | CC | CC | GT | GA | AA | AG |
| CT | CC | CC | CC | CC | CC | GG | GG | AA | AA |
| TT | CC | CC | CC | CC | CC | GG | GG | AA | AA |
| TT | AC | CC | CC | CC | TT | GG | GG | AA | AA |
| TT | CC | CC | CC | CC | CC | GG | GG | AA | AA |
| TT | CC | TC | CC | CC | CC | GG | GA | AA | GA |
| TC | CC | CC | CC | CC | CC | GG | GG | AA | AA |
| TC | CA | CT | CC | CC | CC | GG | GG | AA | AA |
| TT | CC | CC | CT | CC | CC | TG | GA | AA | GA |
| TT | CC | CC | CT | CC | CT | GG | GG | AA | AA |
| TT | CC | CC | CC | CC | CC | TG | GG | AA | AG |
| TT | CC | CC | TC | CT | CC | TG | AG | AA | AA |
| TT | CC | TT | TT | CC | TC | GT | GG | AA | AA |
| TT | CC | CT | CC | CC | CC | TT | AG | AA | AA |
| TT | CC | CT | CT | CC | CC | GG | GG | AA | GA |
| TT | CA | CT | CT | CC | CC | GG | AG | AA | AA |
| TT | CC | CC | CC | CC | CC | TG | GG | AA | AA |
| TT | CC | CC | CC | CC | TC | GG | GA | AA | AA |
| TT | CC | CT | CC | TC | CC | TG | GG | AA | AA |
| TT | CA | CC | CC | CC | CC | GG | GG | AA | AA |
| TC | CC | CC | CT | CC | CC | GT | GG | AA | AA |
| TT | CC | CC | CC | CC | CC | GG | GG | AA | AA |
| TT | CC | TT | CC | CC | TC | GG | GG | AA | AA |
| TT | CC | CC | CC | CC | CC | GG | GG | AA | AA |
| TT | CC | CC | CC | CC | CT | GG | AG | AA | AA |
| TT | CC | CC | CT | CC | CT | GG | AG | AA | AA |
| TT | CC | CC | CC | CC | CC | GG | AG | AA | AG |
| TT | CC | CC | CT | CC | TC | GG | AG | AA | AA |
| CT | CC | TC | CT | CC | CC | GG | GA | AA | AA |
| CT | CC | CC | CT | CC | CC | GG | GG | AA | AA |
| TT | CC | CC | TC | CC | CC | TT | GG | AA | AG |
| TT | CC | TC | CT | CC | TT | GG | GG | AA | GA |
| TT | CC | TC | CC | CC | CC | TG | GG | AA | AA |
| TT | CC | CC | CC | CC | TC | GG | GG | AA | AA |
| TT | CA | CT | CC | CC | CT | GT | GG | AA | GA |
| TT | CC | CC | CC | CC | TC | TT | GG | AA | AA |
| TT | CA | CC | CC | CC | CC | TT | GG | AA | AA |
| TT | CC | CT | CC | CC | TC | TG | GG | AA | AA |
| TT | CA | CC | CC | CC | CC | GG | GA | AA | AG |
| TT | CC | CT | CC | CC | CT | GG | AG | AA | AA |
| TT | CC | CC | CT | CC | CC | GT | GA | AA | GA |
| TT | CC | CC | TC | CC | CT | TG | AG | AG | AA |
| TC | CG | CT | TT | CC | TT | GG | GG | NN | GG |
| CC | GG | CT | CT | CC | TT | TT | GG | NN | GG |
| CC | AG | CT | CT | CC | TT | GT | GG | NN | GG |
| TC | AA | TT | TT | CC | TT | GT | GG | NN | GG |
| CC | GG | CT | CT | CC | TT | NN | GG | NN | GG |
| CC | AG | CT | CC | CC | TT | GG | GG | NN | AG |
| TC | AG | CT | TT | CC | TT | GG | GG | NN | GG |
| CC | GG | CT | TT | CC | TT | GT | GG | NN | GG |
| CC | GG | CT | CT | CC | TT | GT | GG | NN | GG |
| TC | GG | CT | CT | CC | TT | GT | GG | NN | GG |
| CC | GG | CT | TT | CC | TT | GT | GG | NN | GG |
| CC | GG | CC | CT | CC | TT | GG | GG | NN | GG |
| CC | AG | CC | CT | CT | TT | GT | GG | NN | GG |
| CC | NN | CC | TT | CC | TT | TT | GG | NN | GG |
| CC | GG | CT | TT | CC | TT | GT | GG | NN | GG |
| CC | GG | CC | TT | CC | TT | GT | GG | NN | GG |
| CC | CG | CC | TT | CC | TT | GG | GG | NN | GG |
| CC | GG | CT | CT | CC | TT | GG | GG | NN | GG |
| CC | CG | CT | TT | CC | TT | NN | GG | NN | GG |
| CC | CG | TT | TT | CC | TT | GT | GG | NN | GG |
| CC | AG | CT | CT | CC | TT | GG | GG | NN | GG |
| CC | AG | TT | TT | CC | TT | GG | GG | NN | GG |
| CC | AG | TT | TT | CC | TT | GG | GG | NN | GG |
| CC | GG | CT | TT | CT | TT | GG | GG | NN | GG |
| CC | AG | TT | TT | CC | TT | GG | GG | NN | GG |
| CC | AC | CT | TT | CT | TT | GG | GG | NN | GG |
| TC | AA | CT | CT | CC | TT | GG | GG | NN | GG |
| CC | AA | TT | TT | CT | TT | GT | GG | NN | GG |
| TT | CG | CC | TC | CC | TC | GG | AG | AA | GA |
| TT | CC | CC | TT | CC | TC | GG | AG | AA | GA |
| TT | CA | CC | TT | CC | TC | GT | AG | AA | GA |
| TT | CG | CC | TC | CC | TC | GT | AG | AA | GA |
| TT | CG | CC | TC | CC | TC | GG | AG | AA | GA |
| TC | CG | CT | TC | CC | TC | GG | AG | AG | GA |
| TT | CG | CC | TC | CC | TT | GT | AG | AA | GA |
| TT | CG | CC | TT | CC | TC | GG | AG | AA | GA |
| TT | CG | CC | TT | CC | TT | GG | AG | AA | GA |
| TT | CG | CC | TC | CC | TT | GG | AG | AA | GA |
| NN | NN | CC | TC | NN | TT | GT | NN | AA | GA |
| TC | CG | CC | TC | CT | TT | GG | AG | AA | GA |
| TC | CC | CT | TC | CC | TC | GT | AG | AG | GA |
| TC | CG | CC | TC | CC | TT | GG | AG | AG | GA |
| TC | CG | CC | TC | CT | TC | GT | AG | AA | GA |
| TC | CG | CC | TC | CC | TC | GT | AG | AA | GA |
| TT | CG | CT | TC | CT | TT | GT | AG | AA | GA |
| TC | CG | CT | TC | CT | TC | GT | AG | AA | GA |
| TT | CG | CC | TC | CC | TC | GT | AG | AA | GA |
| TC | CA | CC | TC | CC | TC | GT | AG | AA | GA |
| TT | CC | NN | TC | CC | TC | GG | AA | AA | GA |
| TC | CC | CT | TT | CC | TC | GG | AG | AG | GA |
| TC | CC | CC | TC | CC | TT | GG | AG | AG | GA |
| TT | CG | CC | TC | CC | TT | GG | AG | AG | GA |
| TC | CC | CC | TC | CT | TC | GT | AG | AA | GA |
| TT | CG | CC | TC | CC | TT | GG | AG | AG | GA |
| TC | CC | CC | TC | CC | TT | GG | AG | AA | GA |
| TT | CG | CC | TT | CC | TC | GG | AG | AA | GA |

| **rs1834640** | **rs1426654** | **rs6494411** | **rs12594144** | **rs3784651** | **rs881929** | **rs17822931** | **rs16946159** | **rs8072587** | **rs4792928** |
| --- | --- | --- | --- | --- | --- | --- | --- | --- | --- |
| GG | GG | TT | CC | AG | GG | CC | GG | CC | TT |
| GG | GG | TT | CC | AA | GG | CC | GA | CC | TT |
| GG | GG | TT | CC | AG | GG | CC | AG | CC | TT |
| GG | GG | TT | CC | GG | GG | CC | GG | CC | TT |
| GG | GG | TT | CC | AG | GG | CC | GG | CC | TT |
| GG | GG | TT | CC | AG | GG | CC | GG | CC | TT |
| GG | GG | TT | CC | AG | GG | CC | GG | CC | TT |
| GG | GG | CT | CC | AA | GG | CC | GG | CC | TT |
| GG | GG | TT | CC | AA | GG | CC | GA | CG | TT |
| GG | GG | CT | CC | GG | GG | CC | GG | CC | TT |
| GG | GG | CT | CC | GG | GG | CC | GG | CC | TT |
| GG | GG | TT | CC | AG | GG | CC | GG | CC | TT |
| GG | GG | TT | CC | AG | GG | CC | GA | CC | TT |
| GG | GG | TT | CC | AA | GG | CC | GG | CC | TT |
| GG | GG | TT | CC | AG | GG | CC | GG | CC | TT |
| AG | AG | TT | CC | GG | GG | CC | GG | CC | TT |
| GG | GG | TT | CC | GG | GG | CC | GG | CC | TT |
| GG | GG | TT | CC | AG | GG | CC | AG | CC | TT |
| GA | AG | TT | CC | GG | GG | CC | GG | CC | TT |
| GG | GG | TT | CC | AA | GG | CC | GG | CG | TT |
| GG | GG | TT | CC | AG | GG | CC | GG | CC | TT |
| GG | GG | TT | CC | AG | GG | CC | GG | CC | TT |
| GG | GG | TT | CC | GG | GG | CC | GG | CC | TT |
| GG | GG | TT | CC | AG | GG | CC | AG | CC | TT |
| GG | GG | TT | CC | GG | GG | CC | GG | CG | TT |
| GG | GG | TT | CC | AG | GG | CC | GG | CC | TT |
| GA | AG | CC | CC | AA | GG | CC | GG | CC | TT |
| GG | GG | TT | CC | AG | GG | CC | AG | CC | TT |
| GG | GG | TT | CC | GG | GT | CC | GG | CC | TT |
| GG | GG | TT | CC | AG | GG | CC | GG | CC | TT |
| GG | GG | TT | CC | AA | GG | CC | GG | CC | TT |
| GG | GG | TT | CC | GG | GG | CC | GG | CC | TT |
| GG | GG | TT | CC | GG | GG | CC | GG | CC | TT |
| GG | GG | CT | CC | AG | GG | CC | GG | CC | TT |
| GG | GG | TT | CC | AG | GG | CC | GG | CC | TT |
| GG | GG | TT | CC | GG | GG | CC | GG | CC | TT |
| GG | GG | TT | CC | AG | GG | CC | GG | CC | TT |
| GG | GG | TT | CC | AG | GG | CC | GG | CC | TT |
| GG | GG | TT | CC | AG | GG | CC | GG | CC | TT |
| GG | GG | TT | CC | AG | GG | CC | AG | CC | TT |
| GG | GG | TT | CC | AG | GG | CC | GG | CG | TT |
| GG | GG | TT | CC | GG | GT | CC | GG | CC | TT |
| GG | GG | TT | CC | AG | GG | CC | GG | CC | TT |
| GG | GG | TT | CC | AG | GG | CC | GG | CC | TT |
| AG | AG | TT | CC | AG | GG | CC | GG | CC | TT |
| GG | GG | TT | CC | AG | GG | CC | GG | CC | TT |
| GG | GG | CT | CC | GG | GG | CC | AG | CC | TT |
| GG | GG | TT | CC | AG | GG | CC | GG | CC | TT |
| GG | GG | TT | CC | AA | GG | CC | GG | CC | TT |
| GG | GG | TC | CC | AA | GG | CC | GG | CC | TT |
| GG | GG | CT | CC | AA | GG | CC | GA | CC | TT |
| GG | GG | TT | CC | AG | GG | CC | GG | CC | TT |
| GG | GG | TT | CC | AG | GT | CC | GG | CG | TT |
| GG | GG | CT | CC | AG | GG | CC | AG | CG | TT |
| GG | GG | TT | CC | AA | GG | CC | GG | CC | TT |
| GG | GG | TT | CC | GG | GG | CC | GA | CC | TT |
| GG | GG | TT | CC | GG | GG | CC | AG | CC | TT |
| GG | GG | TT | CC | GG | GG | CC | GA | CC | TT |
| GG | GG | TT | CC | AG | GT | CC | GG | CC | TT |
| GG | GG | TT | CC | AG | GG | CC | GG | CC | TT |
| GG | GG | TT | CC | AA | GG | CC | AG | CC | TT |
| GG | GG | TT | CC | GG | GG | CC | GG | CC | TT |
| GG | GG | CT | CC | GG | GG | CC | AG | CC | TT |
| GG | GG | TT | CC | AG | GG | CC | GG | CC | TT |
| GG | GG | TT | CC | AG | GG | CC | GG | CC | TT |
| GG | GG | TT | CC | GG | GG | CC | AG | CC | TT |
| GG | GG | TC | CC | AA | GG | CC | GG | CC | TT |
| GG | GG | TT | CC | GG | GG | CC | GG | CC | TT |
| GG | GG | TT | CC | AG | GG | CC | GG | CC | TT |
| GG | GG | TT | CC | AG | GG | CC | AG | CC | TT |
| GG | GG | TT | CC | AA | GT | CC | GG | CG | TT |
| GG | GG | TT | CC | AG | GG | CC | GG | CC | TT |
| GG | GG | TC | CC | GG | GG | CC | GG | CC | TT |
| GG | GG | TT | CC | AA | GG | CC | GG | CC | TT |
| GG | GG | TC | CC | AG | GG | CC | AG | CC | TT |
| GG | GG | TT | CC | GG | GG | CC | GG | CC | TT |
| GG | GG | TT | CC | AA | GG | CC | GG | CC | TT |
| GG | GG | TT | CC | GG | GG | CC | GG | CC | TT |
| GG | GG | TC | CC | AA | GG | CC | GG | CC | TT |
| GG | GG | TT | CC | GG | GG | CC | GG | CG | TT |
| GG | GG | TT | CC | GG | GG | CC | GG | CC | TT |
| GG | GG | TC | CC | AG | GG | CC | GG | CC | TT |
| GG | GG | TT | CC | AG | GG | CC | GG | CC | TT |
| GG | GG | TT | CC | GG | GG | CC | GG | CC | TT |
| GG | GG | CT | CC | GG | GG | CC | GG | CG | TT |
| GG | GG | TT | CC | GG | GG | CC | GG | CC | TT |
| GG | GG | TT | CC | AG | GG | CC | GA | CC | TT |
| GG | GG | TT | CC | AG | GG | CC | AG | CC | TT |
| GG | GG | TT | CC | GG | GG | CC | GG | CC | TT |
| GG | GG | TT | CC | AG | GG | CC | GG | CC | TT |
| GG | GG | TT | CC | AA | GG | CC | GG | CG | TT |
| GG | GG | TC | CC | AG | GG | CC | GG | CC | TT |
| GG | GG | TT | CC | GG | GG | CC | GA | CC | TT |
| GA | AG | TT | CC | AA | GG | CC | AG | CC | TT |
| GG | GG | TT | CC | GG | GG | CC | GA | CC | TT |
| GG | GG | TT | CC | AG | GG | CC | GG | CC | TT |
| GG | GG | TT | CC | AA | GG | CC | GG | CC | TT |
| GG | GG | TT | CC | AA | GG | CC | GG | CC | TT |
| GG | GG | TT | CC | GG | GG | CC | GA | CG | TT |
| GG | GG | CT | AA | AA | GT | CC | GG | CC | CC |
| AG | GG | CC | AA | AA | GG | CC | GG | CG | TC |
| GG | GG | CT | AA | AA | GG | TC | GG | CG | CC |
| AA | GG | CC | CA | AA | GG | CC | GG | CC | CC |
| AG | GG | CT | AA | AA | GT | TC | GG | CC | CC |
| AG | GG | CC | CA | GG | TT | CC | GG | CG | CC |
| AG | GG | CC | AA | AA | GT | TC | GG | CC | CC |
| GG | GG | CT | AA | AA | GG | CC | GG | CC | CC |
| AG | GG | CT | AA | AA | GG | TC | GG | CC | CC |
| AG | GG | CC | AA | AA | GT | CC | GG | CG | CC |
| AG | GG | CT | AA | AG | GT | TC | GG | CC | CC |
| GG | GG | CC | AA | AG | GT | CC | GG | CC | CC |
| AG | GG | CT | AA | AA | GT | CC | GG | CC | CC |
| GG | GG | CC | AA | AA | GT | TT | GG | CC | CC |
| AG | GG | CT | CA | AA | GT | CC | GG | CC | CC |
| GG | GG | CT | AA | AA | GG | TC | GG | CG | TT |
| GG | GG | CT | AA | AA | GG | TC | GG | CC | TC |
| GG | GA | CT | AA | AA | GG | TT | GG | CC | CC |
| GG | GG | CT | AA | AA | GT | CC | GG | CG | TC |
| AG | GA | CT | AA | AA | GG | TT | GG | CG | TC |
| AA | GA | CC | AA | AA | GT | CC | GG | CC | TC |
| AA | GG | CT | CA | AG | GT | TT | GG | CC | CC |
| AA | GG | CC | CA | AG | GG | CC | GG | CC | TC |
| AG | GA | CT | CA | AG | GT | NN | GG | CC | CC |
| AA | GG | CC | AA | AA | GT | CC | GG | CG | TC |
| GG | GG | CC | AA | AA | GG | TC | GG | CC | TC |
| GG | GG | CT | NN | AG | GG | CC | GG | CG | TC |
| GG | GG | CT | AA | AA | GT | TC | AG | CC | TC |
| AG | GG | CC | AA | AA | GT | CC | GG | CC | TT |
| GG | GG | CT | AA | AG | GT | CC | GG | CG | TT |
| AG | GA | CT | AA | AA | GT | CC | GG | CG | TC |
| AA | GG | CC | AA | AA | TT | NN | GG | CC | CC |
| AG | GG | CT | CA | AG | GG | CC | GG | CC | TC |
| AG | GG | CT | CC | AA | GG | TT | GG | CC | TC |
| GG | GG | CT | CA | AG | GT | TC | GG | CC | TC |
| GG | GG | CT | AA | AG | GT | TT | GG | CG | CC |
| GG | GG | CT | CA | AA | GG | CC | GG | CG | TC |
| AG | GG | CC | CA | AA | GG | CC | GG | CC | CC |
| GG | GG | CT | CA | AA | GG | CC | GG | CG | CC |
| GG | GG | CC | CC | AA | GT | CC | GG | CG | CC |
| AG | GG | CC | CA | AG | GG | TT | GG | CG | CC |
| AA | GG | CT | CA | AG | GG | TC | GG | CG | CC |
| AA | GG | CT | NN | AA | GG | NN | GG | CC | CC |
| AA | GG | CC | CA | AA | GG | TC | GG | CC | CC |
| AG | GG | CC | CA | AA | GT | TT | GG | CC | CC |
| AG | GG | CC | AA | AA | GG | TC | GG | CC | CC |
| AG | GG | CT | CA | AA | GG | CC | GG | CC | CC |
| AG | GG | CT | AA | AA | GT | CC | GG | CG | CC |
| AG | GG | CC | CA | AA | GT | CC | GG | CC | CC |
| AA | GG | CC | AA | AA | GG | TT | GG | CC | CC |
| AG | GG | CC | CA | AA | GG | CC | GG | CC | CC |
| AG | GG | CC | AA | AA | GT | TC | GG | CC | CC |
| GG | GG | CC | AA | AA | GT | TC | GG | CC | CC |
| GG | GG | CC | AA | AG | GT | TC | GG | CG | CC |
| AA | GG | CC | AA | AG | GT | TC | GG | CC | CC |
| GG | GG | CC | AA | AG | GG | CC | GG | GG | CC |
| GG | GG | CC | AA | AA | GG | TT | GG | CC | TC |
| GG | GG | CC | AA | AA | GG | TC | GG | CC | CC |
| GG | GG | CC | AA | AG | GG | TC | GG | CC | CC |
| AA | GG | CT | AA | AA | GG | TC | GG | CG | CC |
| AG | GG | CC | AA | AG | GT | TC | GG | CG | TT |
| AA | GG | CC | AA | AG | GG | NN | GG | NN | CC |
| AG | GG | CC | AA | AA | GT | TC | GG | CC | CC |
| GG | GG | CC | AA | AG | GT | CC | GG | CC | TC |
| GG | GG | CC | AA | AG | GT | CT | GG | CC | CC |
| GG | GG | CC | AA | AG | TT | TT | GG | CC | CC |
| GG | GG | CC | AA | GG | TT | CT | GG | CC | CT |
| GG | GG | CC | AA | AG | TT | CT | GG | CC | CC |
| GG | GG | CC | AC | GG | TT | TT | GG | CC | TT |
| GG | GG | CT | AC | AG | TT | TT | GG | CC | CT |
| GG | GG | CC | AA | GG | GT | CT | AG | CC | CT |
| GG | GG | CC | AA | GG | TT | TT | GG | CC | CT |
| GG | GG | CC | AA | GG | TT | TT | GG | CC | CT |
| AG | GG | CC | AA | GG | TT | TT | GG | CC | CC |
| GG | GG | CC | AA | AG | TT | TT | GG | CC | CT |
| GG | GG | CC | AA | AG | TT | TT | GG | CC | CT |
| GG | GG | CC | AA | GG | TT | TT | GG | CC | CT |
| GG | GG | CC | AA | AG | GT | TT | GG | CC | TT |
| GG | GG | CC | AA | GG | TT | TT | AG | CC | CT |
| GG | GG | CT | AC | GG | TT | TT | GG | CC | CC |
| GG | GG | CC | AA | AG | TT | TT | GG | CC | CT |
| GG | GG | CC | AA | AA | TT | TT | GG | CC | CT |
| GG | GG | CC | AA | GG | TT | TT | GG | CC | CT |
| GG | GG | CC | AA | AA | TT | TT | GG | CC | CC |
| GG | GG | CT | AC | AG | TT | TT | GG | CC | CT |
| GG | GG | CC | AA | AA | TT | TT | GG | CC | CT |
| GG | GG | CC | AA | GG | TT | CT | AG | CC | CC |
| GG | GG | CC | AA | AG | TT | TT | GG | CC | CC |
| GG | GG | CC | AA | GG | GT | TT | GG | CC | CC |
| GG | GG | CC | AA | AG | TT | TT | GG | CC | CT |
| GG | GG | CC | AC | GG | GT | CT | GG | CC | CT |
| GG | GG | CC | AA | GG | TT | TT | AG | CC | CT |
| GG | GG | CC | AA | AG | TT | TT | GG | CC | CC |
| GG | GG | CC | AA | GG | GT | TT | GG | CC | CT |
| GG | GG | CC | AA | AG | TT | TT | GG | CC | CC |
| GG | GG | CT | AC | GG | TT | TT | GG | CC | CT |
| GG | GG | CC | AA | GG | TT | TT | GG | CC | CC |
| GG | GG | CC | AA | AG | TT | TT | GG | CC | CC |
| GG | GG | CT | AC | AA | GT | TT | GG | CC | CT |
| GG | GG | CT | AC | GG | GT | TT | GG | CC | CT |
| GG | GG | CC | AA | GG | TT | TT | GG | CC | TT |
| GG | GG | CC | AA | GG | TT | CT | GG | CC | CT |
| GG | GG | CC | AA | GG | TT | CT | GG | CC | CT |
| GG | GG | CT | AA | GG | TT | CT | GG | CC | CC |
| GG | GG | CC | AA | GG | GT | CT | GG | CC | CC |
| AG | GG | CC | AA | AA | GT | CT | GG | CC | CC |
| GG | GG | CC | AA | GG | TT | TT | GG | CC | CT |
| GG | GG | CC | AA | GG | TT | TT | GG | CC | CT |
| AG | GG | CC | AA | GG | TT | TT | GG | CC | CC |
| GG | GG | CC | AA | GG | TT | TT | GG | CC | CT |
| GG | GG | CC | AA | GG | TT | CT | GG | CC | CC |
| GG | GG | CC | AA | AG | GT | CC | GG | CC | CC |
| GG | GG | CC | AA | AG | TT | TT | GG | CC | CC |
| GG | GG | CC | AA | GG | TT | TT | AG | CC | CC |
| GG | GG | CT | AA | AG | TT | TT | AG | CC | TT |
| GG | GG | CC | AA | AG | GT | TT | GG | CC | CT |
| GG | GG | CT | AC | AG | TT | TT | AA | CC | CT |
| GG | GG | CC | AA | GG | TT | TT | GG | CC | CC |
| GG | GG | CC | AA | GG | TT | TT | GG | CC | CC |
| GG | GG | CC | AA | GG | TT | CT | GG | CC | CC |
| GG | GG | CC | AA | GG | TT | TT | GG | CC | CT |
| GG | GG | CC | AC | AG | TT | TT | GG | CC | CT |
| GG | GG | CC | AA | GG | GT | TT | GG | CC | TT |
| AG | GG | CC | AA | AG | TT | TT | GG | CC | CT |
| GG | GG | CC | AA | GG | TT | TT | GG | CC | CC |
| GG | GG | CC | AA | AG | TT | CC | GG | CC | TT |
| GG | GG | CC | AA | AG | GT | TT | AG | CC | CC |
| GG | GG | CC | AA | GG | TT | TT | GG | CC | TT |
| AG | GG | CC | AA | GG | TT | TT | GG | CC | CC |
| GG | GG | CC | AA | AG | TT | TT | AG | CC | TT |
| GG | GG | CC | AA | GG | TT | TT | GG | CC | TT |
| GG | GG | CC | AA | AG | GT | TT | GG | CC | CC |
| GG | GG | CC | AA | AG | TT | TT | GG | CC | CT |
| AG | GG | CC | AA | AG | TT | TT | GG | CC | CC |
| GG | GG | CC | AA | GG | TT | CT | GG | CC | CT |
| GG | GG | CT | AC | GG | TT | TT | GG | CC | CT |
| GG | GG | CC | AA | AG | TT | CT | GG | CC | TT |
| GG | GG | CT | AA | GG | TT | CT | GG | CC | CC |
| GG | GG | CC | AA | AG | TT | TT | GG | CC | CT |
| GG | GG | CC | AA | GG | TT | TT | GG | CC | TT |
| GG | GG | CC | AA | AG | TT | TT | GG | CC | CT |
| GG | GG | CC | AA | GG | TT | TT | GG | CC | TT |
| GG | GG | CC | AA | AG | TT | TT | GG | CC | CC |
| GG | GG | CC | AA | GG | GT | TT | GG | CC | CC |
| GG | GG | CC | AA | AG | TT | TT | GG | CC | CT |
| GG | GG | CC | AA | GG | TT | TT | GG | CC | CT |
| GG | GG | CC | AA | GG | GT | TT | GG | CC | CT |
| GG | GG | CC | AA | AA | TT | TT | GG | CC | CC |
| GG | GG | CT | AA | AG | TT | TT | GG | CC | CC |
| GG | GG | CC | AA | AG | TT | TT | GG | CC | CC |
| GG | GG | CC | AA | AG | GT | TT | GG | CC | TT |
| GG | GG | CC | AA | GG | TT | TT | GG | CC | CT |
| GG | GG | CC | AA | AG | GT | CT | GG | CC | CT |
| AA | AA | TC | CA | GA | GG | CT | GG | GC | TT |
| AA | AA | TT | CC | AA | GT | TC | GG | GC | TT |
| AA | AA | TT | CC | AA | GG | CC | GG | GG | TT |
| AA | AA | CT | CC | AA | GT | CC | GG | GG | TT |
| AA | AA | CT | AC | AA | GG | CC | GG | GG | TT |
| AA | AA | TT | CC | AA | GG | CC | GG | GC | TT |
| AA | AA | TC | CA | AA | TT | CC | GG | CG | TT |
| AA | AA | TT | CC | AA | GG | CC | GG | CC | TT |
| AA | AA | TT | AC | AA | GT | CC | GG | GG | TT |
| AA | AA | TT | CC | GA | TG | CC | GG | GG | TT |
| AA | AA | CC | AA | AA | TG | CC | GG | GG | TT |
| AA | AA | TT | CC | AA | GG | CC | GG | CG | TT |
| AA | AA | TT | CC | AA | GG | CC | GG | GC | TT |
| AA | AA | TT | CC | AA | TG | CC | GG | GG | TT |
| AA | AA | TT | CC | AA | GT | CC | GG | GG | TT |
| AA | AA | TT | CC | AA | GG | CC | GG | GG | TT |
| AA | AA | TT | AC | AA | GG | CC | GG | CG | TT |
| AA | AA | TT | CC | AA | GG | CT | GG | GG | TT |
| AA | AA | CT | AC | AA | TG | CC | GG | GG | TT |
| AA | AA | TC | CA | AG | TG | CC | GG | GG | TT |
| AA | AA | TT | CC | AA | GT | CC | GG | GG | TT |
| AA | AA | CC | CC | AA | TG | CC | GG | GG | TT |
| AA | AA | TT | AC | GG | GG | CT | GG | CG | TT |
| AA | AA | CT | AC | AG | GG | CC | GG | GC | TT |
| AA | AA | TC | CC | AA | GT | CC | GG | GG | TT |
| AA | AA | TT | CC | AA | TG | CC | GG | GG | TT |
| AA | AA | TT | CC | AA | GT | CC | GG | GG | TT |
| AA | AA | TT | CC | AA | TT | CC | GG | CG | TT |
| AA | AA | TT | CC | AA | TG | CC | GG | GG | TT |
| AA | AA | TT | CC | AA | GG | CC | GG | GG | TT |
| AA | AA | TC | CA | GA | GT | CC | GG | CC | TT |
| AA | AA | TT | CC | AA | GG | TT | GG | GG | TT |
| AA | AA | TT | CC | AA | TG | CT | GG | GG | TT |
| AA | AA | TT | CC | AA | TG | CC | GG | GG | TT |
| AA | AA | TT | CA | GA | GT | CC | GG | GG | TT |
| AA | AA | TC | CA | AA | TG | CC | GG | GG | TT |
| AA | AA | TT | CC | AA | GT | TC | GG | GG | TT |
| AA | AA | TT | CC | GG | GG | CC | GG | GG | TT |
| AA | AA | TT | CC | AA | TG | CC | GG | GG | TT |
| AA | AA | TT | CC | AA | GG | CC | GG | CG | TT |
| AA | AA | CT | AC | AA | GG | CC | GG | GG | TT |
| AA | AA | TC | CA | AA | GG | CC | GG | GG | TT |
| AA | AA | TT | CC | AA | GT | CC | GG | GG | TT |
| AA | AA | TT | CC | AA | GG | CC | GG | GG | TT |
| AA | AA | TC | CA | AA | TG | CC | GG | CG | TT |
| AA | AA | CT | CC | GG | GG | CC | GG | GG | TT |
| AA | AA | TT | CC | AA | TG | CC | GG | CG | TT |
| AA | AA | TT | CC | AA | GG | CC | GG | CG | TT |
| AA | AA | TT | CC | AA | GT | CT | GG | GG | TT |
| AA | AA | TT | AC | AA | TG | CC | GG | GG | TT |
| AA | AA | TT | CC | AG | GG | CC | GG | CG | TT |
| AA | AA | TT | CC | AA | TG | TC | GG | GG | TT |
| AA | AA | TT | CC | AA | GT | TC | GG | GG | TT |
| AA | AA | TT | CC | AA | GG | CC | GG | GG | TT |
| AA | AA | TC | CA | AA | GT | TC | GG | GG | TT |
| AA | AA | TT | CC | AA | GT | CC | GG | GG | TT |
| AA | AA | TT | CC | AA | GG | CC | GG | GG | TT |
| AA | AA | TT | CC | AA | GG | CC | GG | GG | TT |
| AA | AA | TT | CC | AA | TG | CC | GG | CG | TC |
| AA | AA | TT | CA | AA | TG | CC | GG | CC | TT |
| AA | AA | TT | CC | AA | GT | TC | GG | GG | TT |
| AA | AA | TC | CC | AA | GG | CC | GG | GG | TT |
| AA | AA | TT | CC | AA | GG | CC | GG | GG | TT |
| AA | AA | TT | CC | AA | GT | CC | GG | GC | TT |
| AA | AA | TT | CA | AA | GG | CC | GG | GG | TT |
| AA | AA | TT | CC | AA | TT | CC | GG | GG | TT |
| AA | AA | TT | CC | AA | TT | CC | GG | GC | TT |
| AA | AA | TT | CC | AA | GG | CT | GG | GC | TT |
| AA | AA | TT | CC | AA | GG | CC | GG | CC | TT |
| AA | AA | TT | AC | AA | TG | CC | GG | GG | TT |
| AA | AA | TT | CC | AA | GT | CC | GG | CC | TT |
| AA | AA | TT | CC | AA | GT | CC | GG | GG | TT |
| AA | AA | TT | AC | AA | GT | TC | GG | CG | TT |
| AA | AA | TT | CC | AA | TG | CC | GG | CC | TT |
| AA | AA | TT | AC | AA | TG | CC | GG | GG | TT |
| AA | AA | TT | CC | AA | GG | CC | GG | GC | TT |
| AA | AA | TT | CC | AA | GT | CC | GG | GG | TT |
| AA | AA | TT | CC | GA | GG | CC | GG | GG | TT |
| AA | AA | TT | CC | GA | GT | CC | GG | GG | TT |
| AA | AA | TT | AC | AG | GT | CC | GG | GG | TT |
| AA | AA | TT | CC | AA | GT | CC | GG | GG | TT |
| AA | AA | TT | CC | AA | TT | CT | GG | GG | TT |
| AA | AA | TT | CC | AA | TT | TC | GG | GG | TT |
| AA | AA | TC | CC | AA | GT | CC | GG | GG | TT |
| AA | AA | TT | CC | AA | GT | CC | GG | GC | TT |
| AA | AA | TT | CC | AA | GG | CC | GG | GC | TT |
| AA | AA | TT | CC | AA | TG | CC | GG | GG | TT |
| AA | AA | TT | CC | AA | TT | CC | GG | CC | TT |
| GG | GG | CT | CA | GG | GG | TC | AG | CC | TT |
| GG | GG | TT | CC | GG | GT | CC | AA | CC | TC |
| AG | GG | CT | CC | GG | GG | CC | AG | CC | CC |
| GG | GG | CT | CA | GG | GG | CC | AA | CC | TT |
| GG | GG | CC | AA | GG | GG | CC | AA | CC | TT |
| GG | GG | CT | CA | GG | GG | CC | AG | CC | TC |
| GG | GG | TT | CC | GG | GG | CC | AG | CC | TT |
| GG | GG | CT | CA | GG | GG | CC | AA | CC | TT |
| GG | GG | NN | CA | GG | GG | CC | AA | CC | TC |
| GG | GG | CT | AA | GG | TT | TC | AG | CC | TC |
| GG | GG | TT | CA | GG | TT | CC | AG | CC | TT |
| GG | GG | CC | AA | GG | GG | CC | AA | CC | TT |
| GG | GG | TT | CC | GG | GT | CC | AA | CC | TC |
| GG | GG | CT | CA | GG | GG | TC | AA | CC | TC |
| AG | GG | CT | AA | GG | GG | TC | AA | CC | TT |
| GG | GG | TT | CA | GG | GT | TC | AA | CC | TT |
| GG | GG | TT | AA | GG | GG | CC | AA | CC | TT |
| GG | GG | CT | CA | GG | TT | CC | AA | CC | TC |
| GG | GG | TT | CC | GG | TT | CC | AA | CC | TT |
| GG | GG | TT | CC | GG | GT | CC | AG | CC | TT |
| GG | GG | CT | CC | GG | GG | CC | AA | CC | TC |
| GG | GG | CT | CA | GG | GG | CC | AG | CC | TT |
| GG | GG | CT | CA | GG | GT | CC | AA | CC | TT |
| GG | GG | CT | CA | GG | GG | CC | AA | CC | TT |
| GG | GG | TT | CC | GG | GT | CC | AG | CC | TT |
| GG | GG | TT | CC | GG | GG | TC | AA | CC | TC |
| GG | GG | TT | CC | GG | GG | CC | AG | CC | TT |
| GG | GG | CT | CC | GG | GT | TC | AG | CC | TT |
| AA | AA | CT | CA | AA | GT | CC | GG | CC | TC |
| AG | AG | CT | CC | AA | GT | CT | GG | CG | TT |
| AA | AA | CT | CA | AG | GT | CC | GG | CG | TT |
| AA | AA | CT | CA | AG | GT | CT | GA | CG | TT |
| AA | AA | CT | CA | AA | GG | CC | GG | CG | TC |
| AA | AA | CC | CA | AG | GG | CC | GG | CG | TC |
| AA | AA | CT | CC | AA | GT | CT | GG | CG | TT |
| AA | AA | CT | CA | AA | GG | CC | GG | CG | TC |
| AA | AA | CT | CC | AA | GG | CT | GG | CG | TT |
| AA | AA | CT | CC | AG | GG | CC | GG | CG | TT |
| AA | NN | NN | NN | AA | NN | CT | NN | CC | TT |
| AA | AA | CT | CC | AG | GG | CT | GG | CG | TT |
| AA | AA | CT | CC | AA | GT | CC | GG | CC | TT |
| AA | AA | CT | CA | AG | GG | CT | GG | CC | TC |
| AA | AA | CT | CC | AA | GT | CC | GG | CC | TT |
| AA | AA | CT | CC | AA | GG | CT | GG | CC | TT |
| AA | AA | CT | CC | AG | GG | CT | GG | CC | TC |
| AA | AA | CT | CC | AG | GG | CT | GG | CG | TT |
| AA | AA | CT | CA | AA | GG | CT | GG | CC | TT |
| AA | AA | CT | CA | AG | GG | CC | GG | CC | TT |
| AG | AA | CC | CA | AA | GT | CT | GG | CG | TT |
| AA | AA | CT | CC | AA | GT | CC | GG | CC | TT |
| AA | AA | CT | CC | AG | GT | CT | GG | CG | TT |
| AG | AG | CT | CA | AA | GG | CT | GG | CG | TT |
| AG | AG | CT | CA | AG | GG | CC | GG | CG | TT |
| AA | AG | CT | CA | AA | GG | CT | GG | CG | TT |
| AA | AA | CT | CC | AG | GT | CC | GG | CG | TT |
| AG | AA | CT | CA | AG | GT | CT | GA | CG | TC |

| **rs9908046** | **rs1369290** | **rs6054465** | **rs2069945** | **rs310644** | **rs715605** | **rs8137373** | **rs1557553** | **rs4892491** | **rs11156577** |  |
| --- | --- | --- | --- | --- | --- | --- | --- | --- | --- | --- |
| CC | AA | TT | GG | CC | AA | AA | CC | GG | CT | 1 |
| CC | AA | TT | CG | CC | AA | AA | CC | GG | TT | 1 |
| CC | AA | TT | GC | CC | AA | AA | CC | GG | CC | 1 |
| CC | AA | TT | AG | CC | AA | AA | CC | AA | CC | 1 |
| CC | AA | TT | GG | CC | AA | AA | CC | AA | TT | 1 |
| CT | AA | TT | CC | CC | AA | AG | CC | GG | TT | 1 |
| CC | AC | TT | GA | CC | AG | AA | CC | AA | CT | 1 |
| CC | AC | TT | GG | CT | AG | AA | CC | AA | TT | 1 |
| CC | AA | TT | GG | CC | AA | AA | CC | AG | TT | 1 |
| CC | AA | TT | CG | CT | AA | AA | CC | AA | CC | 1 |
| CC | AA | TT | CG | CC | AA | AA | CC | AA | CC | 1 |
| CC | AA | TT | GG | CC | AA | AA | CC | AG | CC | 1 |
| CC | AA | TT | GG | CC | AG | AA | CC | GG | TT | 1 |
| CC | AC | TT | GG | CC | AA | AG | CT | GG | TT | 1 |
| CC | AA | TT | GA | CC | AA | AA | CT | AG | CC | 1 |
| CC | AA | TT | GG | CC | AA | AG | CC | AG | CC | 1 |
| CC | AA | TT | GG | CC | AA | AA | CC | GG | CC | 1 |
| CC | AA | TT | AA | CC | AA | AG | CC | GG | CC | 1 |
| CC | AA | TT | GG | CT | AA | AA | CC | GG | CC | 1 |
| CC | AA | TT | GG | CT | AA | AA | CC | GG | CC | 1 |
| CC | AC | TT | GG | CC | AA | AG | CC | GG | CC | 1 |
| CC | CC | TT | CG | CC | AA | AG | CC | GG | TT | 1 |
| CC | AA | CT | GC | CC | AA | AA | CC | GG | TT | 1 |
| CC | AA | TT | GG | CC | AA | AA | CC | GG | CC | 1 |
| CT | AA | TT | GG | CC | AA | AA | CC | GG | CC | 1 |
| CC | AA | TT | GG | CC | AA | AA | CC | GG | CC | 1 |
| CC | AA | TT | GG | CC | AA | AA | CC | GG | CC | 1 |
| CC | AC | TT | GG | CC | AA | AA | CC | GG | TT | 1 |
| CC | AA | TT | CC | CC | AA | AA | CC | AA | CC | 1 |
| CC | AA | TT | GG | CC | AA | AA | CC | AA | CC | 1 |
| CC | AA | TT | CA | CC | AA | AA | CC | AA | TT | 1 |
| CC | CC | TT | AC | CC | AA | AA | CC | AA | CC | 1 |
| CC | AA | TT | GG | CC | AA | AA | CC | GG | CC | 1 |
| CC | AA | TT | GG | CC | AA | AG | CC | AG | CC | 1 |
| CC | AA | TT | GG | CC | AA | AA | CC | GG | TT | 1 |
| CC | AA | TT | AG | CC | AA | AA | CC | AG | CT | 1 |
| CC | AA | TT | GG | CC | AA | AG | CC | GG | TT | 1 |
| CC | AA | TT | CG | CC | AA | AA | CC | AG | CC | 1 |
| CC | AA | TT | GG | CC | AA | AA | CC | GG | TT | 1 |
| CC | AC | CT | GC | CC | AA | AA | CC | AA | TT | 1 |
| CC | AA | TT | GG | CC | AG | AG | CC | GG | TT | 1 |
| CC | AA | TT | GG | CT | AA | AA | CC | AA | CC | 1 |
| CC | AA | CT | CG | CC | AA | AA | CT | GG | CC | 1 |
| CC | AA | TT | GG | CC | AA | AA | CC | AA | TT | 1 |
| CC | AA | CT | GG | CT | AG | AA | CC | GG | TT | 1 |
| CC | AA | TT | GG | CT | AA | AA | CC | AG | CC | 1 |
| CC | AA | TT | GC | CC | AA | AA | CC | GG | CC | 1 |
| CC | AA | TT | GG | CT | AA | AA | CC | GG | CT | 1 |
| CC | AC | TT | GG | CC | AG | AA | CT | GG | CC | 1 |
| CC | AA | TT | GG | CC | AA | AA | CC | GG | CC | 1 |
| CC | AC | TT | GG | CC | AG | AA | CC | GG | CC | 1 |
| CC | AA | TT | GG | CC | AA | AA | CT | GG | CC | 1 |
| CC | AC | TT | GG | CC | AA | AA | CT | AA | CC | 1 |
| CT | AC | TT | GC | CC | AA | AA | CC | AA | TT | 1 |
| CC | AA | TT | GG | CC | AA | AA | CC | AG | CT | 1 |
| CC | AC | TT | GG | CT | AG | AA | CC | GG | CC | 1 |
| CC | AA | TT | CG | CC | AA | AG | CC | AA | CC | 1 |
| CC | AC | TT | GG | CC | AA | AA | CC | AG | TT | 1 |
| CC | AC | TT | GG | CC | AA | AG | CC | GG | TT | 1 |
| CT | AA | TT | AG | CC | AA | AG | CC | AG | TT | 1 |
| CC | AA | TT | GG | CT | AA | AG | CT | AA | CC | 1 |
| CC | AA | TT | GG | CC | AA | AA | CT | GG | CC | 1 |
| CC | AA | TT | GG | CC | AA | AA | CC | AA | CC | 1 |
| CC | AA | TT | GG | CC | AG | AA | CT | AA | CT | 1 |
| CC | AA | TT | CC | CC | AA | AA | CC | AA | CC | 1 |
| CC | AA | TT | GG | CC | AA | AA | CC | AA | CC | 1 |
| CC | AA | TT | GG | CC | AA | AA | CC | AA | CC | 1 |
| CT | AA | TT | GG | CC | AA | AA | CC | GG | CC | 1 |
| CC | AA | TT | GG | CC | AA | AA | CC | GG | CC | 1 |
| CC | AA | TT | GG | CC | AA | AG | CT | AA | CT | 1 |
| CC | AC | TT | GG | CT | AA | AA | CT | GG | CC | 1 |
| CC | AA | TT | GG | CC | AG | AA | CC | GG | TT | 1 |
| CC | AA | TT | CC | CC | AA | AA | CT | GG | CT | 1 |
| CT | AC | TT | CA | CC | AA | AG | CC | GG | TT | 1 |
| CC | AC | TT | GG | CC | AG | AA | CC | AG | TT | 1 |
| CC | AA | CT | GG | CC | AA | AG | CC | AA | TT | 1 |
| CC | AC | TT | GC | CC | AA | AA | CC | AA | CT | 1 |
| CC | AC | TT | CC | CC | AG | AA | CC | AA | CC | 1 |
| CC | AC | TT | GG | CC | AA | AG | CC | GG | TT | 1 |
| CC | AA | TT | GC | CC | AA | AA | CC | GG | TT | 1 |
| CC | AC | TT | GG | CC | AA | AA | CC | GG | CT | 1 |
| CC | AA | TT | GG | CC | AA | AA | CC | GG | CC | 1 |
| CC | AA | CT | GG | CC | AA | AA | CC | GG | TT | 1 |
| CC | AA | TT | GG | CC | AA | AA | CC | AA | CC | 1 |
| CC | AA | TT | GG | CC | AA | AA | CC | GG | CC | 1 |
| CC | AA | TT | GC | CC | AA | AA | CC | GG | CT | 1 |
| CC | AA | TT | GG | CC | AA | AA | CC | GG | CC | 1 |
| CC | AA | TT | GG | CC | AA | AA | CC | GG | CC | 1 |
| CC | AA | CT | GC | CC | AA | AA | CT | GG | CT | 1 |
| CC | AA | TT | CG | CT | AG | AA | CC | GG | CC | 1 |
| CC | AA | TT | GG | CT | AG | AA | CC | AG | CC | 1 |
| CC | AA | TT | GC | CC | AG | AG | CC | AG | CC | 1 |
| CC | AA | TT | GG | CC | AA | AA | CC | GG | TT | 1 |
| CC | AA | TT | CC | CC | AA | AG | CC | AG | CT | 1 |
| CC | AA | TT | AG | CC | AG | AG | CC | AA | CC | 1 |
| CC | AA | TT | CG | CC | AA | AG | CC | AG | CT | 1 |
| CC | AA | TT | GG | CC | AA | AA | CT | AA | TT | 1 |
| CC | AA | TT | GG | CC | AA | AA | CT | GG | CT | 1 |
| CC | AA | TT | CG | CC | AA | AA | CC | GG | CC | 1 |
| CC | NN | TT | CC | TT | AA | AA | TT | GG | TT | 1 |
| CC | NN | TT | CC | TT | AA | AA | TT | GG | TT | 1 |
| CC | NN | TT | CC | TT | AA | AA | CT | AA | TT | 1 |
| CC | NN | TT | GC | TT | AA | GA | TT | AG | TT | 1 |
| CC | NN | TT | CC | TT | AA | AA | TT | GG | TT | 1 |
| CC | NN | TT | CC | TT | AA | AA | TT | AA | TT | 1 |
| CC | NN | TT | CC | TT | AA | AA | TT | GG | TT | 1 |
| CC | NN | CT | CC | TT | AA | AA | TT | GG | TT | 1 |
| CC | NN | TT | CC | TT | AA | AA | TT | GG | TT | 1 |
| CC | NN | TT | CC | TT | AA | AA | TT | AA | TT | 1 |
| CC | NN | TT | CC | TT | AA | AA | TT | GG | TT | 1 |
| CC | NN | TT | CC | TT | AA | AA | TT | AA | TT | 1 |
| CC | NN | TT | CC | TT | AA | AA | TT | GG | TT | 1 |
| CC | NN | TT | CC | TT | AA | AA | TT | GG | TT | 1 |
| CC | NN | TT | CC | CT | AA | AA | TT | GG | TT | 1 |
| CC | NN | TT | GC | TT | AA | AA | CT | AG | TT | 1 |
| CC | NN | TT | GC | TT | AA | AA | TT | GG | TT | 1 |
| CC | NN | TT | GC | TT | AA | AA | TT | GG | TT | 1 |
| CC | NN | TT | CC | TT | AA | AA | CT | GG | TT | 1 |
| CC | NN | TT | CC | TT | AA | AA | TT | AA | TT | 1 |
| CC | NN | TT | GG | TT | AA | AA | CT | GG | TT | 1 |
| CC | NN | TT | CC | TT | AA | AA | CT | GG | TT | 1 |
| CC | NN | CT | CC | CT | AA | AA | TT | GG | TT | 1 |
| CC | NN | TT | CC | TT | AA | AA | TT | GG | TT | 1 |
| CC | NN | CT | CC | TT | AA | AA | TT | GG | TT | 1 |
| CC | NN | TT | GC | TT | AA | AA | TT | GG | TT | 1 |
| CC | NN | TT | GC | TT | AA | AA | TT | GG | TT | 1 |
| CC | NN | TT | CC | TT | AA | AA | TT | AA | TT | 1 |
| CC | NN | TT | CC | TT | AA | AA | TT | AG | CT | 1 |
| CC | NN | TT | GC | CT | AA | GG | CC | AG | TT | 1 |
| CC | NN | CT | GC | TT | AA | AA | TT | AG | TT | 1 |
| CC | NN | TT | GC | TT | AA | AA | TT | GG | TT | 1 |
| CC | NN | TT | CC | CT | AA | AA | TT | GG | TT | 1 |
| CC | NN | TT | CC | TT | AA | AA | TT | GG | TT | 1 |
| CC | NN | TT | GC | TT | AA | AA | TT | AA | TT | 1 |
| CC | NN | TT | CC | TT | AA | AA | TT | GG | TT | 1 |
| CC | NN | CT | CC | CT | AA | AA | TT | AG | CT | 1 |
| CC | NN | CT | CC | CT | AA | AA | TT | GG | TT | 1 |
| CC | NN | TT | GC | TT | AA | AA | TT | GG | TT | 1 |
| CC | NN | CC | GC | TT | AA | AA | CT | GG | TT | 1 |
| CC | NN | CC | CC | TT | AA | AA | CT | GG | TT | 1 |
| CC | NN | CT | GG | TT | AA | AA | TT | AG | TT | 1 |
| CC | NN | TT | GC | TT | AA | AA | TT | GG | TT | 1 |
| CC | NN | CT | CC | TT | AA | AA | TT | GG | TT | 1 |
| CC | NN | CT | CC | TT | AA | AA | TT | GG | CC | 1 |
| CC | NN | CT | CC | TT | AA | AA | CT | GG | CC | 1 |
| CC | NN | TT | CC | TT | AA | AA | TT | GG | TT | 1 |
| CC | NN | CT | CC | TT | AA | AA | CT | GG | TT | 1 |
| CC | NN | CT | CC | TT | AA | AA | TT | GG | CT | 1 |
| CC | NN | CT | GG | TT | AA | AA | CT | GG | TT | 1 |
| CC | NN | TT | GC | TT | AA | AA | TT | GG | TT | 1 |
| CC | NN | TT | GC | TT | AA | AA | TT | GG | TT | 1 |
| CC | NN | TT | GC | TT | AA | AA | TT | GG | TT | 1 |
| CC | NN | TT | CC | TT | AA | AA | TT | GG | TT | 1 |
| CC | NN | TT | CC | TT | AA | AA | TT | GG | TT | 1 |
| CC | NN | TT | GG | TT | AA | AA | TT | GG | TT | 1 |
| CC | NN | TT | CC | TT | AA | AA | TT | AG | TT | 1 |
| CC | NN | TT | CC | TT | AA | AA | TT | GG | CT | 1 |
| CC | NN | TT | GG | TT | AA | AA | TT | GG | TT | 1 |
| TC | NN | TT | CC | TT | AA | AA | TT | GG | TT | 1 |
| CC | NN | TT | GC | TT | AA | AA | TT | GG | TT | 1 |
| CC | NN | TT | CC | TT | AA | AA | TT | GG | TT | 1 |
| CC | NN | CT | GG | TT | AA | AA | TT | AA | TT | 1 |
| CC | NN | TT | GC | TT | AA | AA | TT | GG | TT | 1 |
| CC | CC | TT | CC | TT | AA | GG | CC | AA | TT | 1 |
| CC | CC | TT | GG | TT | AA | GG | TT | AA | TT | 1 |
| CC | CC | TT | CG | TT | AA | GG | CC | AA | TT | 1 |
| CC | CC | CT | CC | TT | AA | GG | TT | AA | TT | 1 |
| CC | CC | CC | CG | TT | AA | GG | CC | AA | TT | 1 |
| CC | CC | CT | CC | TT | AA | GG | CT | AA | TT | 1 |
| CC | CC | TT | CC | TT | AA | GG | CC | AA | TT | 1 |
| CC | CC | TT | CG | TT | AA | GG | CC | AA | TT | 1 |
| CC | CC | CC | CC | TT | AA | GG | CC | AA | TT | 1 |
| CC | CC | TT | CC | TT | AA | GG | CT | AA | TT | 1 |
| CC | CC | TT | CG | TT | AA | GG | CC | AA | TT | 1 |
| CC | CC | CT | CG | CT | AA | GG | TT | AA | TT | 1 |
| CC | CC | CT | GG | TT | AA | GG | CC | AA | TT | 1 |
| CC | CC | TT | CG | TT | AA | GG | CT | AA | TT | 1 |
| CC | CC | TT | CG | TT | AA | GG | TT | AA | TT | 1 |
| CC | CC | TT | CC | TT | AA | GG | TT | AA | TT | 1 |
| CT | CC | CT | GG | TT | AA | GG | CC | AA | TT | 1 |
| CC | CC | CT | CC | TT | AA | GG | CT | AA | TT | 1 |
| CC | CC | CT | CC | TT | AA | GG | CC | AA | TT | 1 |
| CC | CC | TT | CC | TT | AA | GG | CC | AA | TT | 1 |
| CC | CC | TT | CC | TT | AA | GG | CT | AA | TT | 1 |
| CC | CC | TT | CC | TT | AA | GG | CC | AA | TT | 1 |
| CC | CC | TT | CC | TT | AA | GG | CC | AA | TT | 1 |
| CT | CC | TT | CC | TT | AA | GG | CT | AA | TT | 1 |
| CC | CC | TT | CG | TT | AA | GG | CT | AA | TT | 1 |
| TT | CC | TT | CG | TT | AA | GG | CC | AA | TT | 1 |
| CT | CC | TT | CC | TT | AA | GG | CC | AA | TT | 1 |
| CT | CC | TT | CC | TT | AA | GG | CC | AA | TT | 1 |
| CT | CC | CT | CC | TT | AA | GG | CC | AA | TT | 1 |
| CC | CC | TT | CC | TT | AA | GG | CC | AA | TT | 1 |
| CT | CC | TT | CC | TT | AA | AA | CC | AA | TT | 1 |
| CC | CC | CT | CG | TT | AA | GG | CT | AA | TT | 1 |
| CT | CC | CT | CC | TT | AA | GG | CT | AA | TT | 1 |
| CC | CC | TT | CC | TT | AA | GG | CT | AA | TT | 1 |
| CC | CC | CC | CG | TT | AA | GG | CC | AA | TT | 1 |
| CC | CC | CT | CC | TT | AA | GG | CT | AA | TT | 1 |
| CC | CC | CT | GG | TT | AA | AG | CC | AA | TT | 1 |
| CC | CC | CT | CC | TT | AA | GG | CC | AA | TT | 1 |
| CC | CC | CT | CG | TT | AA | AG | CC | AA | TT | 1 |
| CT | CC | CT | CC | TT | AA | GG | CC | AA | TT | 1 |
| CC | CC | TT | CC | TT | AA | GG | CC | AA | TT | 1 |
| CC | CC | TT | CC | TT | AA | GG | CT | AA | TT | 1 |
| CC | CC | TT | CG | TT | AA | GG | TT | AA | TT | 1 |
| CT | CC | TT | CC | TT | AA | GG | CC | AA | TT | 1 |
| CC | CC | TT | CC | TT | AA | GG | CT | AA | TT | 1 |
| CC | CC | TT | CC | TT | AA | AG | CC | AA | TT | 1 |
| CC | CC | CT | CG | TT | AA | GG | CT | AA | TT | 1 |
| CC | CC | CC | CC | TT | AA | GG | CT | AA | TT | 1 |
| CC | CC | TT | CC | TT | AA | GG | CC | AA | TT | 1 |
| CT | CC | TT | CC | TT | AA | GG | TT | AA | TT | 1 |
| CC | CC | TT | CC | TT | AA | AA | CC | AA | TT | 1 |
| CC | CC | TT | CC | TT | AA | GG | CT | AA | TT | 1 |
| CC | CC | CT | CG | TT | AA | GG | TT | AG | TT | 1 |
| CC | CC | TT | CC | TT | AA | GG | CC | AA | TT | 1 |
| CC | CC | TT | CC | TT | AA | GG | CC | AA | TT | 1 |
| CC | CC | TT | CG | TT | AA | GG | CT | AA | TT | 1 |
| CC | CC | CT | CG | TT | AA | GG | CC | AA | TT | 1 |
| CC | CC | TT | CG | TT | AA | GG | CC | AA | TT | 1 |
| CC | CC | TT | CC | TT | AA | GG | CC | AA | TT | 1 |
| CC | CC | CC | CG | TT | AA | GG | CT | AA | TT | 1 |
| CC | CC | CT | CC | TT | AA | GG | CC | AA | TT | 1 |
| CC | CC | TT | CC | TT | AA | GG | CC | AA | TT | 1 |
| CC | CC | TT | CC | TT | AA | GG | CT | AA | TT | 1 |
| CC | CC | CT | CC | TT | AA | GG | CT | GG | TT | 1 |
| CC | CC | TT | CG | TT | AA | GG | CC | AA | TT | 1 |
| CC | CC | TT | CC | TT | AA | GG | CT | AA | TT | 1 |
| CC | CC | TT | CG | TT | AA | GG | CT | AA | TT | 1 |
| CC | CC | TT | CG | TT | AA | GG | CT | AA | TT | 1 |
| CC | CC | CT | CC | TT | AA | GG | CT | AA | TT | 1 |
| CC | CC | CT | CC | TT | AA | GG | CC | AA | TT | 1 |
| CC | CC | TT | CC | TT | AA | GG | CT | AA | TT | 1 |
| CT | CC | CT | CC | TT | AA | GG | CT | AA | TT | 1 |
| CC | CC | TT | CC | TT | AA | GG | CC | AA | TT | 1 |
| CC | CC | TT | CC | CT | AA | GG | CC | AA | TT | 1 |
| CT | CC | CT | CG | TT | AA | GG | CT | AA | TT | 1 |
| CC | CC | TT | CC | TT | AA | AG | CC | AA | TT | 1 |
| CC | CC | TT | CC | TT | AA | GG | TT | AA | TT | 1 |
| CC | CC | TT | CG | TT | AA | GG | TT | AA | TT | 1 |
| CC | CC | TT | CC | TT | AA | GG | CC | AA | TT | 1 |
| CC | CC | CT | CC | TT | AA | GG | CT | AA | TT | 1 |
| CT | CC | TT | CG | TT | AA | GG | CC | AA | TT | 1 |
| CC | CC | CT | CC | TT | AA | GG | CC | AA | TT | 1 |
| CT | CC | CT | CG | TT | AA | GG | CC | AA | TT | 1 |
| CC | CC | TT | CC | TT | AA | GG | CC | AA | TT | 1 |
| CC | CC | CC | CC | TT | AA | AG | CT | AA | TT | 1 |
| CT | CC | TT | CC | TT | AA | GG | CC | AA | TT | 1 |
| CC | CC | TT | CC | TT | AA | GG | CC | AA | TT | 1 |
| CC | CC | TT | CC | TT | AA | AG | CC | AA | TT | 1 |
| CT | CC | TT | GG | TT | AA | AG | TT | AA | TT | 1 |
| CC | CC | TT | GC | CT | AA | GG | CC | GG | CC | 1 |
| CC | CC | TT | CG | TT | AA | AG | CC | GA | TT | 1 |
| CC | AC | TT | GC | TT | AA | GA | CC | GG | TT | 1 |
| CC | CC | CT | CG | TT | GA | GG | CC | AG | TT | 1 |
| CC | CC | TC | GG | TT | AA | AG | CC | GG | TT | 1 |
| CC | CC | TT | CG | TT | AG | GA | CC | GG | TT | 1 |
| TC | CC | TT | CG | CT | AA | GA | CC | GG | TT | 1 |
| CC | CC | TT | GC | TT | AA | GG | CC | GG | NN | 1 |
| CC | CA | TT | GC | TT | AA | GG | CC | GG | TT | 1 |
| CC | CC | TT | CC | TT | AA | AA | CC | GG | NN | 1 |
| TC | CC | TC | CG | TT | AA | AA | CC | GG | CC | 1 |
| CC | CC | TT | GG | TT | AA | GG | CC | GG | TT | 1 |
| CC | CC | TC | GC | TT | GG | GA | CT | GG | CT | 1 |
| CC | CC | TT | GG | TT | AA | GG | CC | AA | TT | 1 |
| CC | CC | TT | CG | TT | AA | GG | CC | GG | TT | 1 |
| CC | CC | TT | CC | CT | AA | GG | TC | GG | TT | 1 |
| CC | CC | TT | GG | TT | AA | GG | CC | AA | TT | 1 |
| CC | CC | TT | GG | TT | AA | GG | CC | GG | TT | 1 |
| CC | CC | CT | CC | CT | AA | AG | CC | GG | TT | 1 |
| CC | CC | TT | CC | TT | AA | GG | TC | GG | TT | 1 |
| CC | CC | TT | CG | TT | AA | GG | CC | AG | TT | 1 |
| CC | CC | TT | CG | TC | GA | GG | CC | GG | CT | 1 |
| CC | CC | CC | CG | TT | AG | GG | CC | GG | CT | 1 |
| CC | CC | TT | CG | TT | AA | GG | CC | AG | TT | 1 |
| CT | CC | CT | CG | TT | AA | GG | CC | GG | TT | 1 |
| CC | CC | TT | GC | TT | AA | AG | CC | AG | TT | 1 |
| CT | CC | TT | GG | CT | AA | GG | TC | GG | CC | 1 |
| CT | CC | TC | GG | TT | AA | AA | CC | GG | TT | 1 |
| CC | CC | CT | GC | TT | AA | AG | CC | AG | TT | 1 |
| CC | CC | TC | CC | CC | GA | AA | CC | GG | CC | 1 |
| CC | CC | TT | CG | TT | AA | GA | CC | GG | CT | 1 |
| CC | CC | TC | CG | CT | AA | GG | CC | GG | TT | 1 |
| CC | CC | CT | GC | TT | AA | GG | CC | AG | CT | 1 |
| CC | CC | TT | GC | TT | AA | GG | CC | AG | NN | 1 |
| CC | CC | TT | CC | TT | AA | AG | CC | NN | NN | 1 |
| CC | CC | TT | GG | TT | GA | AA | CC | GG | TT | 1 |
| CC | CC | CT | CG | CT | AA | GA | CC | GG | TT | 1 |
| CC | CC | TT | CC | TT | AA | AA | CC | GG | CC | 1 |
| CC | CC | TT | GG | TT | AA | GG | CC | GG | TT | 1 |
| CC | CC | TT | CC | TT | AA | GG | CC | GG | CC | 1 |
| CC | AC | TT | CC | TT | AG | GG | CC | AA | TT | 1 |
| CT | CC | TT | GC | TT | AA | GG | CC | AA | TT | 1 |
| CC | CC | TT | GG | TT | AA | GG | CC | AA | TT | 1 |
| CC | CC | TT | CC | TC | AG | GG | CC | GG | TT | 1 |
| TC | CC | TT | GC | TT | GA | AA | CT | GG | TT | 1 |
| CC | CA | TT | CC | TT | AA | GA | CC | GG | TT | 1 |
| TC | CC | TT | CC | TT | AA | GG | CC | GG | CT | 1 |
| CC | CC | TT | GC | TT | AA | GG | TC | GG | TT | 1 |
| CC | CC | TT | GG | TT | AA | AG | CT | NN | NN | 1 |
| CC | CC | TT | GG | TT | AA | AG | CC | GG | TT | 1 |
| CC | CC | TT | GC | TT | AA | GG | CC | NN | NN | 1 |
| CC | CC | TT | GG | TT | AA | GA | CC | AG | TT | 1 |
| TC | CC | TT | CG | CT | AA | AG | CC | GG | TT | 1 |
| CC | CC | TT | GC | TT | AA | AA | CC | GG | TT | 1 |
| CC | CC | CT | GC | TT | AA | GA | CT | GG | CT | 1 |
| CC | CC | TT | CC | TT | AA | GG | CC | GG | TT | 1 |
| CT | CA | CT | CC | TT | AA | GG | CC | AG | TT | 1 |
| CC | CC | TT | CG | TT | AA | GA | CC | GG | TT | 1 |
| CC | CC | TT | CC | TT | AA | GG | CC | GG | TT | 1 |
| CC | CC | TC | CG | TT | AA | GG | CC | AG | TT | 1 |
| CC | CC | TT | GG | TT | AA | GG | TC | GG | TT | 1 |
| CC | CC | TT | CC | CT | AA | GG | CC | GG | TT | 1 |
| CC | CC | TT | CG | TT | AA | GG | CC | AG | TT | 1 |
| CC | CC | TT | GC | TT | AA | GG | CC | GG | CT | 1 |
| CC | CC | CT | CG | TT | AA | AA | CC | GG | TT | 1 |
| CC | AC | TT | GC | TT | AA | GA | CC | GG | TT | 1 |
| CC | CA | TC | CG | TT | AA | AG | CC | GG | TT | 1 |
| CC | CC | TT | GC | TT | AA | GG | CC | GG | TT | 1 |
| CC | CC | TC | GC | TT | AA | GA | CC | GG | TT | 1 |
| CC | CC | TC | GG | TT | AA | GG | CC | NN | NN | 1 |
| CC | CC | TT | GC | TT | AA | GG | TC | NN | NN | 1 |
| CC | CC | TT | CC | TT | AA | GG | CC | AG | CT | 1 |
| CC | CC | TT | GG | TT | AA | GG | CC | GG | CC | 1 |
| CC | CC | TT | CG | TT | AA | GG | TC | GG | TT | 1 |
| CC | CC | TT | CC | TT | AA | AG | CC | AG | TT | 1 |
| CC | CC | TT | CG | TT | AA | GA | CC | GG | TT | 1 |
| CC | CC | TT | GG | TC | AA | AG | CC | AA | TT | 1 |
| CC | CC | TT | GC | CC | AA | GA | TC | GG | TT | 1 |
| CC | CC | TT | CG | TT | AA | AG | TC | GG | TT | 1 |
| CC | CC | TT | GC | TT | AA | AA | TC | AG | TT | 1 |
| CC | CC | TT | GC | TT | AA | GG | CC | GG | TT | 1 |
| CC | CC | TT | CC | TT | AA | GG | CC | GG | TT | 1 |
| CC | CC | CT | CG | TT | AA | AA | CC | AG | TT | 1 |
| CC | CC | TT | GG | TT | AA | GG | CC | GG | TT | 1 |
| CC | CC | TT | GC | TT | AA | GG | TT | AG | TT | 1 |
| CC | CC | TT | GG | TT | AA | GG | CC | AA | TT | 1 |
| CC | CC | CT | CC | TT | AA | AG | CC | GG | TT | 1 |
| CC | AC | TT | GG | TT | AA | GA | CC | GG | TT | 1 |
| TT | CC | CC | GG | CC | GG | GG | CT | AA | CC | 1 |
| TT | CC | CC | AA | CC | AG | GG | CC | GG | CC | 1 |
| TT | CC | CC | GG | CC | GG | GG | CC | GG | CC | 1 |
| TT | CC | CC | GG | CC | GG | GG | CC | GG | CC | 1 |
| TT | CC | CC | AA | CC | GG | GG | CC | GG | CC | 1 |
| TT | CC | CC | GA | CT | GG | GG | CC | AG | TT | 1 |
| TT | CC | CC | GA | CC | GG | GG | CT | AA | CC | 1 |
| TT | CC | CC | AA | CC | GG | GG | CT | GG | CC | 1 |
| TT | CC | CC | GA | CC | AG | GG | CT | GG | CC | 1 |
| TT | CC | CC | AA | CC | GG | GG | CT | GG | CC | 1 |
| TT | CC | CC | GG | CC | GG | GG | TT | AA | CC | 1 |
| TT | CC | CC | GG | CC | GG | GG | CC | GG | CC | 1 |
| TT | CC | CC | GA | CC | GG | GG | CT | GG | CC | 1 |
| TT | CC | CC | GA | CC | GG | GG | CT | GG | CC | 1 |
| TC | CC | CC | AA | CC | GG | GG | CC | GG | CC | 1 |
| TT | CC | CC | GA | CC | GG | GG | CC | GG | CC | 1 |
| TT | CC | CC | AA | CC | GG | GA | CC | GG | CC | 1 |
| TT | CC | CC | GG | CC | GG | GG | CC | GG | CC | 1 |
| TT | CC | TT | GC | TT | GG | GG | CC | GG | CC | 1 |
| TT | CC | CC | GC | CC | AG | GG | CC | AG | CT | 1 |
| TT | CC | CC | GG | CC | AG | GG | CT | AG | CC | 1 |
| TT | CC | CC | GG | CC | GG | GG | CT | GG | TT | 1 |
| TT | CC | CC | GA | CC | GG | GG | CC | AG | CC | 1 |
| TT | CC | CC | GA | CC | GG | GG | CC | AG | CC | 1 |
| TT | CC | CC | GA | CT | AG | GG | CC | AG | CC | 1 |
| TT | CC | CC | GA | CT | GG | GG | CC | AA | CC | 1 |
| TT | CC | CC | GG | CC | GG | GG | CC | GG | CC | 1 |
| TT | CC | CC | AA | CT | GG | GG | CT | AG | CC | 1 |
| CC | AC | TT | CG | TT | AA | GG | CC | GG | CT | 0 |
| CC | AC | TT | CG | TT | AG | GG | CT | GA | CT | 0 |
| CC | AC | TT | CG | TT | AG | GG | CC | GG | CT | 0 |
| CC | AC | TT | CA | TT | AA | GG | CC | GA | CC | 0 |
| CC | AC | TT | CC | TT | AA | GG | CC | GG | CT | 0 |
| CC | AC | TT | CG | TT | AA | GA | CT | GG | CT | 0 |
| CC | AC | TC | CC | TT | AA | GA | CC | GG | CT | 0 |
| CC | AC | TT | CC | TT | AA | GG | CC | GG | CT | 0 |
| CC | AC | TT | CA | TT | AA | GA | CC | GA | CT | 0 |
| CC | AC | TT | CG | TT | AA | GG | CC | GA | CT | 0 |
| NN | AC | TC | CG | TT | AA | GG | CT | NN | NN | 0 |
| CC | AC | TT | CA | TC | AA | GG | CC | GA | CT | 0 |
| CC | AC | TT | CA | TT | AG | GG | CC | GA | CT | 0 |
| CC | AC | TC | CA | TT | AA | GA | CT | GA | CT | 0 |
| CC | AC | TT | CG | TT | AA | GA | CT | GA | CT | 0 |
| CC | AC | TC | CG | TC | AA | GG | CC | GG | CT | 0 |
| CC | AC | TT | CA | TT | AA | GA | CT | GG | CT | 0 |
| CC | AC | TT | CG | TT | AA | GA | CT | GA | CT | 0 |
| CC | AC | TT | CG | TT | AA | GA | CT | GA | CT | 0 |
| CC | AC | TC | CG | TT | AA | GG | CC | GG | CT | 0 |
| CC | AA | TT | CG | TT | AA | GG | CC | GG | NN | 0 |
| CC | AC | TC | CG | TT | AA | GA | CC | GG | CT | 0 |
| CC | AC | TT | CG | TT | AA | GA | CT | GA | CC | 0 |
| CC | AC | TT | CG | TC | AA | GG | CC | GG | CT | 0 |
| CC | AC | TC | CG | TC | AA | GG | CC | GG | CC | 0 |
| CC | AC | TT | CG | TC | AA | GG | CC | GG | CT | 0 |
| CC | AC | TT | CG | TT | AA | GG | CT | GA | CT | 0 |
| CC | AC | TT | CG | TT | AA | GG | CT | GG | CT | 0 |
